# Paradoxical antidepressant effects of alcohol are related to acid sphingomyelinase and its control of sphingolipid homeostasis

**DOI:** 10.1007/s00401-016-1658-6

**Published:** 2016-12-20

**Authors:** Christian P. Müller, Liubov S. Kalinichenko, Jens Tiesel, Matthias Witt, Thomas Stöckl, Eva Sprenger, Jens Fuchser, Janine Beckmann, Marc Praetner, Sabine E. Huber, Davide Amato, Christiane Mühle, Christian Büttner, Arif B. Ekici, Irena Smaga, Lucyna Pomierny-Chamiolo, Bartosz Pomierny, Malgorzata Filip, Volker Eulenburg, Erich Gulbins, Anbarasu Lourdusamy, Martin Reichel, Johannes Kornhuber

**Affiliations:** 1 Department of Psychiatry and Psychotherapy, University Clinic, Friedrich-Alexander-University of Erlangen-Nuremberg, Schwabachanlage 6, 91054 Erlangen, Germany; 2 Bruker Daltonik GmbH, Bremen, Germany; 3 Institute of Human Genetics, Friedrich-Alexander-Universität Erlangen-Nürnberg, Erlangen, Germany; 4 Chair of Toxicology, Faculty of Pharmacy, Jagiellonian University Medical College, Krakow, Poland; 5 Institute of Pharmacology, Polish Academy of Sciences, Laboratory of Drug Addiction Pharmacology, 12 Smetna, Krakow, 31-343 Poland; 6 Institute of Biochemistry, Friedrich-Alexander-University of Erlangen-Nuremberg, 91054 Erlangen, Germany; 7 Department of Molecular Biology, University of Duisburg-Essen, Essen, Germany; 8 Department of Surgery, College of Medicine, University of Cincinnati, 231 Albert Sabin Way, Cincinnati, OH 45267-0558 USA; 9 Division of Child Health, Obstetrics and Gynaecology, School of Medicine, University of Nottingham, Nottingham, NG7 2UH UK; 10 Department of Nephrology and Hypertension, Friedrich-Alexander-University Erlangen-Nuremberg, Schwabachanlage 12, 91054 Erlangen, Germany

**Keywords:** Alcohol, Drug instrumentalization, Depression, Acid sphingomyelinase, Sphingomyelin, Nucleus accumbens

## Abstract

Alcohol is a widely consumed drug that can lead to addiction and severe brain damage. However, alcohol is also used as self-medication for psychiatric problems, such as depression, frequently resulting in depression-alcoholism comorbidity. Here, we identify the first molecular mechanism for alcohol use with the goal to self-medicate and ameliorate the behavioral symptoms of a genetically induced innate depression. An induced over-expression of acid sphingomyelinase (ASM), as was observed in depressed patients, enhanced the consumption of alcohol in a mouse model of depression. ASM hyperactivity facilitates the establishment of the conditioned behavioral effects of alcohol, and thus drug memories. Opposite effects on drinking and alcohol reward learning were observed in animals with reduced ASM function. Importantly, free-choice alcohol drinking—but not forced alcohol exposure—reduces depression-like behavior selectively in depressed animals through the normalization of brain ASM activity. No such effects were observed in normal mice. ASM hyperactivity caused sphingolipid and subsequent monoamine transmitter hypo-activity in the brain. Free-choice alcohol drinking restores nucleus accumbens sphingolipid- and monoamine homeostasis selectively in depressed mice. A gene expression analysis suggested strong control of ASM on the expression of genes related to the regulation of pH, ion transmembrane transport, behavioral fear response, neuroprotection and neuropeptide signaling pathways. These findings suggest that the paradoxical antidepressant effects of alcohol in depressed organisms are mediated by ASM and its control of sphingolipid homeostasis. Both emerge as a new treatment target specifically for depression-induced alcoholism.

**Electronic supplementary material:**

The online version of this article (doi:10.1007/s00401-016-1658-6) contains supplementary material, which is available to authorized users.

## Introduction

Alcohol is a major psychoactive drug in western societies [24]. The majority of adults regularly consume alcohol with the risk of developing an alcohol use disorder (AUD) [13, 63], accompanied by severe brain pathologies [68]. It was shown that alcohol can be instrumentalized, i.e., used to achieve goals that would be impossible to achieve or that require a greater workload without alcohol use [47, 48]. Alcohol use can serve numerous instrumentalization goals, such as the facilitation of social interaction, mating behavior, or stress coping. One of the most important goals is the self-medication for innate or induced psychiatric problems, such as for depression and/or anxiety disorders [5, 47, 54]. There is a high comorbidity of depression and alcohol use disorder with bi-directional trajectories [55]. Depression can either be induced after an AUD has manifested, or a depression emerges first and alcohol is used subsequently to ameliorate the symptoms of depression [19, 64]. While the neuropharmacology of alcohol is well known [17, 73], the neurobiological mechanisms for the potential antidepressant effects of alcohol and their instrumentalization remain poorly understood [34].

Together with cholesterol and glycerophospholipids, sphingolipids are the most common lipids in brain membranes [30]. Sphingolipids form lipid rafts and signaling platforms, which are membrane compartments that are enriched in G-protein-coupled receptors [20, 72]. Changes in the composition of the lipid rafts and platforms directly affect receptor affinity, signaling and subsequent internalization [14, 20]. Acid sphingomyelinase (ASM) hydrolyses sphingomyelin (SM) to ceramide (Cer) and phosphorylcholine, and thus represents a major regulator of sphingolipid metabolism [31]. Sphingolipids are involved in fundamental cellular processes, such as differentiation, senescence, and apoptosis [22], as well as in behavioral adaptations [29, 53] and psychiatric disorders [51, 65]. It was shown that depressed patients and alcohol dependent patients during withdrawal show increased ASM activity in peripheral blood mononucleated cells and in the plasma [36, 58, 59]. Transgenic mice over-expressing ASM (tgASM) display a reduced neurogenesis in the hippocampus and a depression/anxiety phenotype, which can be reversed by the functional inhibitors of ASM [21, 38].

Here, we identify a new molecular mechanism for the paradoxical antidepressant effects of alcohol when used voluntarily to self-medicate and ameliorate the behavioral symptoms of a genetically induced depression. We show that alcohol drinking normalizes ASM function and re-establishes sphingolipid- and monoamine homeostasis in the nucleus accumbens of depressed mice. Thus, sphingolipid homeostasis emerges as a new mechanism to control depression-AUD comorbidity.

## Materials and methods

### Animals

In ASM transgenic mice (tgASM), the murine *Smpd1* cDNA was expressed under the control of the ubiquitous CAG promoter. A loxP-flanked STOP cassette was included between the promoter and the transgene so that the expression can be conditionally regulated by the action of Cre recombinase. The conditional transgene was introduced into the deleted *Hprt* gene locus of E14 embryonic stem cells. The transgenic mice were generated and back-crossed for at least 5 generations to C57BL/6 mice. The transgene was constitutively expressed by crossing the mice with E2A-Cre mice expressing Cre recombinase under the control of an E2A promoter with a C57BL/6 background [21]. The WT mice used in this study were floxed Cre-positive mice without the ASM transgene. Heterozygous ASM-deficient mice (*Smpd1* ^+*/*−^, hetKO ASM) [27] were originally obtained from Dr. R. Kolesnick, Memorial Sloan-Kettering Cancer Center, New York, NY, USA. Male and female tgASM and hetKO ASM and respective wild type (WT) mice (8–12 weeks old) were studied in gender balanced designs. All experiments were carried out in accordance with the National Institutes of Health guidelines for the humane treatment of animals and the European Communities Council Directive (86/609/EEC), and were approved by the local governmental commission for animal health.

### Alcohol drinking and alcohol deprivation effect

Alcohol drinking was tested in naïve tgASM, hetKO ASM and respective WT mice using a two-bottle free-choice drinking paradigm. Mice were single housed and each cage was equipped with two bottles that were constantly available, one of which contained tap water and the other bottle contained alcohol at various concentrations. After an acclimatization period of two weeks to establish a drinking baseline, the animals received alcohol at increasing concentrations of 2, 4, 8, 12, and 16 vol% for 4 days each. Thereafter, the alcohol concentration was maintained at 16 vol% for 12–14 days. To measure the alcohol deprivation effect, alcohol was removed for 3 weeks (with both bottles containing tap water) before it was re-introduced for 4 days. This procedure was repeated one (hetKO ASM) or two (tgASM) more times. The bottles were changed and weighed daily. The consumed amount of alcohol relative to body weight and preference vs. water were measured [11, 69].

### Taste preference test

Alcohol-experienced animals (42 free-choice drinking days) were used for this test. Sucrose (0.5 and 5%) and quinine (2 and 20 mg/dl) preferences were measured in a two-bottle free-choice test vs. water three days after the last alcohol exposure. Each dose was offered for 3 days with the position of the bottles being changed and weighed daily with a 1-day wash out between the sucrose and quinine testing [11, 69].

### Conditioned place preference

The establishment of alcohol-induced conditioned place preference (CPP) was tested in naïve tgASM, hetKO ASM and respective WT mice with a time-course CPP model described previously [12]. Subsequently, food-induced CPP was tested in naïve tgASM and WT mice following the same procedure. The week before testing, the animals were accustomed to food pellets (45 mg sucrose pellets) with five pellets/day. Before each conditioning trial, animals were food deprived for 24 h. During conditioning, they received 20 pellets during the 30 min conditioning trials, or no food during pseudo-conditioning trials. After each learning trial, they received free access to food for 2 h in their home cages (see Supplementary Information (SI)).

### Loss of righting reflex

Alcohol-naïve animals were administered with 3.5 g/kg (i.p.) alcohol in saline (*v*_inj_ = 20 ml/kg) to induce a loss of the righting reflex (LORR), and were immediately placed in an empty cage. LORR was observed when the animal becomes ataxic and stopped moving for at least 30 s. The animal was then placed on its back. Recovery from alcohol administration was defined as the animal being able to right itself three times within a minute. A 2 h cut off was used. The time taken for the animal to lose its righting reflex and the time to recovery from the alcohol’s effects were recorded [11, 75].

### Blood alcohol determination

Alcohol naïve animals received an alcohol injection (3.0 g/kg, i.p.) and 20 µl blood samples were obtained from the submandibular vein 1, 2, and 3 h after injection. The blood samples were directly mixed with 80 µl 6.25% (w/v) trichloroacetic acid. After centrifugation, 15 µl of the supernatant were subjected to enzymatic alcohol determination using the alcohol dehydrogenase method as described elsewhere [56, 75].

### Emotional behavior after alcohol exposure

#### Free-choice consumption

Alcohol drinking was established in naïve tgASM and WT mice using a two-bottle free-choice drinking paradigm as described above but with bottle measurements every second day [9, 60]. After 12 days of drinking 16 vol% alcohol, the animals were tested with a battery of behavioral tests including open field, elevated plus maze, novelty suppressed feeding, and the forced swim test, as described previously [10, 70] (see SI). They continued drinking alcohol in their home cages throughout the testing.

#### Forced alcohol exposure

Naïve tgASM or hetKO ASM and respective WT mice received an injection with alcohol (2 g/kg; i.p.) or saline 30 min before each behavioral test (total: 5 injections). Behavioral tests were then performed as described above [10, 70] (see SI). The mice were tested in a pseudorandom order and were moved to the behavioral suite adjacent to the housing room 1 h before testing. The mice were returned to their home cages at the end of each test and were allowed at least 2 days to recover before further testing [10, 70]. After the behavioral testing commenced the animals were sacrificed, after which blood was collected and brain tissue was harvested for an analysis of ASM activity.

### Acid sphingomyelinase activity

Acid sphingomyelinase activity was measured in the dorsal hippocampus and serum of either free-choice alcohol drinking or forced alcohol exposure-treated tgASM, hetKO ASM, or respective WT mice using the fluorescent substrate BODIPY-FL-C12-SM (N-(4,4-difluoro-5,7-dimethyl-4-bora-3a,4a-diaza-s-indacene-3-dodecanoyl) sphingosyl phosphocholine, D-7711, Invitrogen/Life Technologies) method, with three replicates for each sample as described previously [29] (see SI).

### MALDI imaging mass spectrometry

tgASM and WT mice were trained to drink 16 vol% alcohol in a two-bottle free-choice drinking procedure as described above. The control groups received water in both bottles the entire time. Immediately after a 20 day reinstatement of 16 vol% alcohol drinking, the animals were sacrificed, after which the brains were harvested and snap-frozen with dry ice and then stored at −80 °C. For MALDI Imaging MS, coronal brain slices were prepared at the level of either the nucleus accumbens (Nac; area of interest 1) or the dorsal hippocampus (DH; area of interest 2). All slides were prepared using the ImagePrep (Bruker Daltonik GmbH, Bremen) using α-cyano-4-hydroxycinnamic acid as the matrix and the standard method for this matrix for the preparation device. For MALDI-MS measurements, the prepared slides were mounted onto a Slide Adapter (Bruker Daltonik GmbH, Bremen) and loaded into the dual source of a 12T FTICR MS (SolariX XR, Bruker Daltonics). The data were analyzed using FlexImaging (4.1), and DataAnalysis (4.2) (both Bruker Daltonik GmbH) [66] (see SI).

### Post mortem neurochemistry

For an estimation of the brain tissue monoamine levels, tgASM and WT mice were investigated after free-choice alcohol consumption and emotional behavior testing. One day after behavioral testing and immediately after continued free-choice 16 vol% alcohol consumption, the mice were sacrificed and decapitated, and the brains were harvested and snap frozen with CO_2_. For the neurochemical analysis, the brain areas were dissected: ventral striatum, dorsal hippocampus, and prefrontal cortex. The tissue was homogenized in 0.5 M perchloric acid, it was then centrifuged, filtered, and stored at −80 °C until an analysis of monoamine content was done, as previously described [52, 57] (see SI).

### RNA-seq analysis, co-expression network analysis and module characterization

tgASM and WT mice, which had been either drinking 16 vol% alcohol or water as described above, were tested for mRNA expression in the DH using RNA-seq. Immediately after a 20 day reinstatement of 16 vol% alcohol drinking, the mice were sacrificed by cervical dislocation, and brains were harvested and snap frozen on dry ice. The areas of interest were cut from coronal sections of 1-mm thickness according to the anatomical coordinates taken from the Franklin & Paxinos [15] mouse brain atlas. The total RNA was isolated with an RNeasy Mini Kit (Qiagen, Hilden, Germany) according to the manufacturers’ instructions (see SI).

Before and during the library preparation, the RNA quality was ascertained using a 2100 Bioanalyzer system (Agilent Technologies). Barcoded and strand-specific whole transcriptome sequencing libraries were prepared from 100 ng of DNase digested total RNA using an Ovation Human FFPE RNA-seq System (NuGEN) according to the manufacturer’s instructions. The human rRNA was depleted using human specific NuGEN InDA-C oligonucleotides. The pooled libraries were sequenced on a HiSeq 2500 platform (Illumina) generating 82 million 101 bp single-end reads on average.

After alignment against the reference genome GRCm38 using STAR v.2.4.0i [7], the absolute read counts for all Ensembl genes (version 75) were determined with HTSeq count v.0.6.1. [1]. A differential expression analysis accounting for the paired design with respect to the primary cell cultures was performed using the DESeq 2 package v.1.10.1. [9, 43]. The *p* values were corrected using the false discovery rate method.

A network analysis was performed with the R package weighted gene co-expression networks (WGCNA) on normalized and transformed data obtained from the RNA-seq. Genes with excessive zero counts (80% of total sample) were removed, and the genes with a variance above the median variance were retained for the network analysis. We used a robust correlation measure bicor to create a correlation matrix containing all pair-wise correlations between all genes across all samples (see SI).

### Superoxide dismutase protein expression

The effects of free-choice 16 vol% alcohol drinking and abstinence after drinking on superoxide dismutase (SOD) activity and SOD1 and SOD2 protein expression in the cerebellum of the tgASM and WT mice were analyzed as previously described [67] (see SI). The cerebellum was chosen as an exploratory target for the SOD assay due to its high sensitivity to the toxic morphological effects of alcohol [16] mediated at least in part by oxidative stress [32], the involvement of the cerebellum in depression and addiction [46], and the high correlation of ASM activity between the dorsal hippocampus and cerebellum [29].

### Statistics

All quantitative data were expressed as mean ± SEM. The data were analyzed using ANOVAs (for repeated measures where appropriate) followed by pre-planned comparisons using Fisher’s LSD tests with Bonferroni-correction or by post hoc Newman–Keuls’ test when appropriate. For single group comparisons of normally distributed data, *t* tests were used. Although sex differences are well known in alcoholism-related behaviors [42], we did not see significant sex differences in the major parameters of this study (Suppl. Tab. 1). Therefore, the data were collapsed for analysis. A significance level of *p* < 0.05 was used.

## Results

### Enhanced alcohol consumption in mice with ASM hyperactivity

**Fig. 1:**
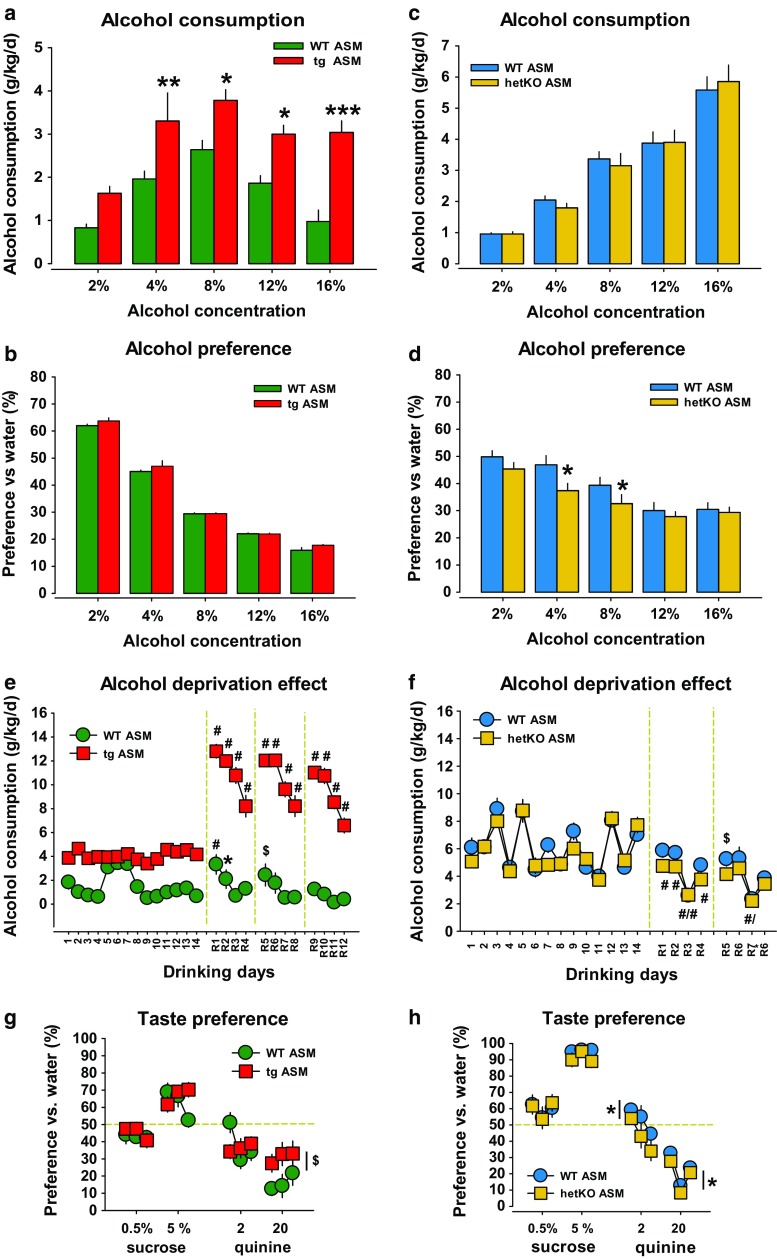
Alcohol consumption is enhanced in mice overexpressing acid sphingomyelinase (tgASM) and is reduced in mice with acid sphingomyelinase deficiency (heterozygous ASM knock out—hetKO ASM). The *error bars* show the mean ± SEM. **a**, **b** Alcohol consumption and preference vs. water are enhanced in tgASM mice at different doses of alcohol in a two-bottle free-choice drinking paradigm (*n* = 16/group). A two-way ANOVA for alcohol consumption showed a significant effect for the genotype (*F*_1,150_ = 51.737, *p* < 0.001) and dose (*F*_4,150_ = 13.438, *p* < 0.001) but showed no interaction. A two-way ANOVA for alcohol preference showed a significant effect for the genotype (*F*_1,150_ = 4.40, *p* = 0.037) and dose (*F*_4,150_ = 1002.19, *p* < 0.001) but no interaction (*p < 0.05, **p < 0.01, ***p < 0.001 vs. wild type; WT). **c**, **d** Alcohol preference vs. water—but not total consumption—is attenuated in hetKO ASM mice at different doses of alcohol in a two-bottle free-choice drinking paradigm (*n* = 12–15/group). A two-way ANOVA for alcohol preference showed a significant effect for the genotype (*F*_1,120_ = 9.846, *p* = 0.002) and dose (*F*_4,120_ = 18.488, *p* < 0.001) but no interaction (*p < 0.05, vs. WT). **e** tgASM mice show persistent high consumption of a 16 vol% alcohol solution (*n* = 16/group). Three-week withdrawal periods (*dashed green lines*) repeatedly induced an alcohol deprivation effect (ADE). This effect was significantly enhanced in tgASM mice, suggesting potentiated susceptibility to alcohol withdrawal effects on drinking motivation. A two-way ANOVA for the ADE showed a significant effect for the genotype (*F*_1,30_ = 324.621, *p* < 0.001) and days (*F*_25,750_ = 37.438, *p* < 0.001) and a genotype × test day interaction (*F*_25,750_ = 33.763, *p* < 0.001) (*p < 0.05, ^$^ *p* < 0.01, ^#^ *p* < 0.001 vs. day 14—last day before withdrawal). **f** Two three-week withdrawal periods (*dashed green lines*) further reduced alcohol drinking in hetKO ASM mice (*n* = 12–14/group). A two-way ANOVA showed a significant effect for genotype (*F*_1,528_ = 6.535, *p* = 0.011) and days (*F*_21,528_ = 25.354, *p* < 0.001) but no interaction (*p < 0.05, ^$^ *p* < 0.01, ^#^ *p* < 0.001 vs. day 14—last day before withdrawal). **g** tgASM mice show no difference in preference for sweetness (sucrose) or avoidance of the low dose bitter solution (quinine) (*n* = 16/group). However, the avoidance of a high dose bitter solution was attenuated (genotype: *F*_1,90_ = 8.472, *p* = 0.004). **h** hetKO ASM mice show no difference in preference for sweet substances compared to WT (*n* = 12–14/group). However, hetKO ASM mice show a stronger avoidance of the bitter taste at 2 mg/dl (genotype: *F*_1,72_ = 4.192, *p* = 0.044) and 20 mg/dl quinine solution (genotype: *F*_1,72_ = 5.201, *p* = 0.026)

We have previously shown that the overexpression of ASM and the resulting increase in Cer inhibit neurogenesis in the hippocampus and induce depression-like behavior in mice [21]. Here, we report that tgASM mice drink significantly more alcohol in a two-bottle free-choice paradigm than wild type (WT) controls (Fig. 1a). They also show a small enhancement in the alcohol preference vs. water at the genotype level which, however, reached significance only at the ANOVA level (factor genotype: *F*_1,150_ = 4.40, *p* = 0.038; Fig. 1b). Water consumption alone was not different in tgASM and WT mice when they had only water available (Suppl. Figure 1). To test alcohol consumption in animals with reduced ASM activity, we used heterozygous ASM knock-out mice (hetKO ASM). Homozygous ASM KO mice develop neurodegeneration similar to Niemann–Pick disease after only a few month of age [4, 40], while hetKO ASM mice show no such symptoms. In hetKO ASM mice, the total amount of consumed alcohol did not significantly change (Fig. 1c). Alcohol preference vs. water, in contrast, showed a significant decline vs. WT but only at a medium concentration of 4–8 vol% alcohol (Fig. 1d).

In tgASM mice, enhanced consumption of 16 vol% alcohol was preserved over time. Repeated withdrawal from alcohol for three weeks and subsequent reinstatement induced an alcohol deprivation effect (ADE) in WT mice. This effect was significantly potentiated in tgASM mice after the first, second, and third withdrawal (Fig. 1e). HetKO ASM mice, however, maintained their alcohol consumption over time. The withdrawal and reinstatement of alcohol drinking resulted in a significant decline in the amount of alcohol consumed (Fig. 1f). Neither tgASM nor hetKO ASM mice showed an altered preference for sweetness (Fig. 1g, h). However, tgASM mice showed a significantly reduced avoidance of a strong bitter tasting quinine solution at a dose of 20 mg/dl, while bitter taste avoidance was enhanced in hetKO ASM mice at 2 and 20 mg/dl solutions. Overall, the marginally altered sensitivity to bitterness cannot explain the strong alcohol preference of tgASM mice or the attenuated preference in hetKO ASM mice. Altogether, these findings suggest that ASM hyperfunction results not only in a depression-like phenotype [21] but also in a much enhanced consumption of alcohol and potentiated sensitivity for the reinstatement of drinking after withdrawal.

### Accelerated onset of conditioned alcohol effects in mice with ASM hyperactivity

**Fig. 2:**
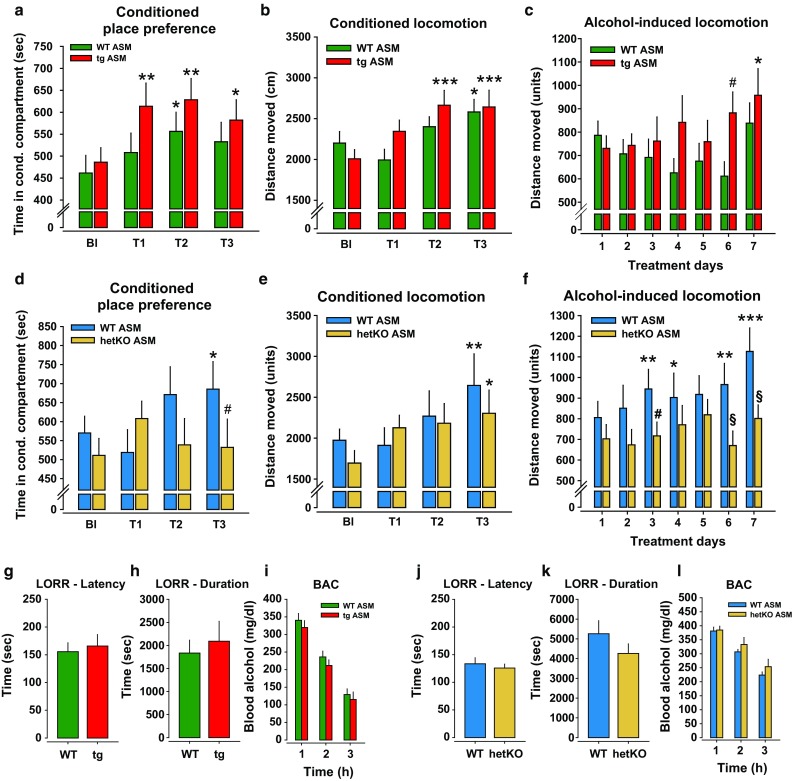
Acid sphingomyelinase (ASM) facilitates the establishment of alcohol-conditioned behavioral effects. The *error bars* show the mean ± SEM. **a** A conditioned place preference (CPP) is established after a repeated conditioning with alcohol (2 g/kg, i.p.; ANOVA, factor time: *F*_3,78_ = 5.676, *p* = 0.001). However, the alcohol CPP is established more quickly in mice overexpressing ASM (tgASM, *n* = 13) than in wild type (WT, *n* = 15) mice. The animals were tested for time spent in the alcohol paired compartment during the baseline (Bl) and after one (T1), three (T2) and seven (T3) conditioning sessions with alcohol (*p < 0.05, **p < 0.01, vs. Bl). **b** Conditioned hyperactivity is expressed during CPP test trials (ANOVA, factor time: *F*_3,78_ = 9.027, *p* < 0.001). Conditioned hyperactivity emerged faster in tgASM (*n* = 13) than in WT (*n* = 15) mice (*p < 0.05, ***p < 0.001 vs. Bl). **c** There was no difference in the acute locomotor response to alcohol between tgASM and WT mice. The acute locomotor effects changed with repeated injections (ANOVA, factor time: *F*_6,156_ = 2.459, *p* = 0.027). While there was a decline in locomotor responding after repeated alcohol injections in WT mice (*n* = 15), tgASM mice (*n* = 13) showed a significant sensitization (*p < 0.05 vs. BL; ^#^ *p* < 0.05 vs. WT). **d** Alcohol CPP is not established in mice with ASM hypoactivity (hetKO ASM; n = 14) but was established in WT mice (*n* = 14; ANOVA, time × genotype interaction: *F*_3,72_ = 3.575, *p* = 0.018). The animals were tested for the time spent in the alcohol-paired compartment during the baseline and after one (T1), three (T2) and seven (T3) conditioning sessions with alcohol (2 g/kg, i.p.; *p < 0.05 vs. BL; ^#^ *p* < 0.05 vs. WT). **e** Conditioned hyperactivity is expressed during CPP test trials (ANOVA, factor time: *F*_3,72_ = 5.074, *p* = 0.003) to a similar degree in WT (*n* = 12) and hetKO ASM mice (*n* = 14; (*p < 0.05, **p < 0.01 vs. Bl). **f** There was no difference in the acute locomotor response to alcohol between hetKO ASM (*n* = 14) and WT mice (*n* = 12) during the first conditioning trial. Acute locomotor effects changed with repeated injections (ANOVA, factor time: *F*_6,144_ = 3.936, *p* = 0.001). While there was a significant sensitization after repeated injection in WT mice, there was no sensitization effect observed in hetKO ASM mice (ANOVA, factor genotype: *F*_1,24_ = 5.105, *p* = 0.033; *p < 0.05, **p < 0.01, ***p < 0.001 vs. BL; ^#^ *p* < 0.05, ^§^ *p* < 0.01 vs. WT)). No effect of ASM hyperactivity (**g**, **h)** *n* = 8–11/group) or hypoactivity (**j**, **k**) (*n* = 6–16) on the sedating effect of alcohol (3.5 g/kg, i.p.), measured by the loss of righting reflex (LORR), was seen. (**i**, **l**) No effect of ASM hyperactivity (*n* = 8/group) or hypoactivity (*n* = 6–10/group) on alcohol bioavailability, as shown in the blood alcohol concentration after an i.p. injection of alcohol (3 g/kg, i.p.), was seen

The establishment of controlled drug use for drug instrumentalization requires distinct drug memories [47, 49, 61]. To test the role of ASM in drug memories we measured the time course for the establishment of conditioned reinforcing and conditioned locomotor effects and locomotor sensitization [11, 12]. We found that tgASM mice show a faster establishment of conditioned place preference (CPP) compared to WT controls (Fig. 2a). A significant CPP was observed in tgASM mice following only a single conditioning trial with alcohol. The repeated pairing of a spatial context with alcohol induced a conditioned hyperlocomotion in mice (Fig. 2b). This behavior was also established more quickly in tgASM mice than in WT mice, i.e., after only 3 conditioning trials at T2. The locomotor effects of an acute alcohol injection (2 g/kg, i.p.) did not differ between tgASM and WT mice. Repeated alcohol injections (2 g/kg, i.p.) had no significant effect on locomotor activity compared to the first administration in WT mice. In tgASM mice, however, there was a significant sensitization of locomotor activity (Fig. 2c). Alcohol is a pharmacological reinforcer [68]. To test whether ASM hyperactivity would specifically facilitate the reinforcing action of alcohol or that of all reinforcers, we measured the time course of food CPP establishment in tgASM mice. Repeated food delivery induced a significant preference for the food-paired compartment and conditioned hyperlocomotion in this compartment. However, there was no difference between tgASM and WT mice (Suppl. Figure 2).

While a significant CPP was established after seven alcohol conditioning trials in WT mice, reduced ASM activity prevented the establishment of an alcohol CPP completely (Fig. 2d). However, not all conditioned alcohol effects were absent in hetKO ASM mice. Conditioned hyperlocomotion was established in WT and hetKO ASM mice at comparable speeds (Fig. 2e). Locomotor activity after an acute alcohol injection (2 g/kg, i.p.) did not differ between hetKO ASM and WT mice (Fig. 2f). While the locomotor response to an alcohol challenge (2 g/kg; i.p.) sensitized after seven treatments in WT mice, there was no sensitization in hetKO ASM mice. Taken together, these findings suggest that ASM hyperactivity facilitates the establishment of the conditioned reinforcing-, conditioned locomotor- and locomotor sensitization effects of repeated alcohol treatment [28, 29]. From this, ASM emerges as a specific facilitator of the reinforcing effects of alcohol.

### ASM activity does not control alcohol’s sedating effects or bioavailability

In depressed people, alcohol may be consumed to reach a level of sedation that attenuates rumination and perception of a negative emotional state. We, therefore, tested whether ASM plays a role in the sedating effects of alcohol using the LORR test. We found that neither ASM hyperactivity in tgASM mice nor the ASM hypofunction in hetKO ASM mice altered the sedating effects of alcohol measured in the time to achieve sedation and in the duration of the sedation after a single injection with 3.5 g/kg (i.p.) alcohol (Fig. 2g, h, j, k). Neither the ASM hyperactivity nor hypoactivity altered the bioavailability of alcohol in mice (Fig. 2i, l).

### Free-choice alcohol drinking restores ASM homeostasis and reverses depression-like behavior in tgASM mice

**Fig. 3:**
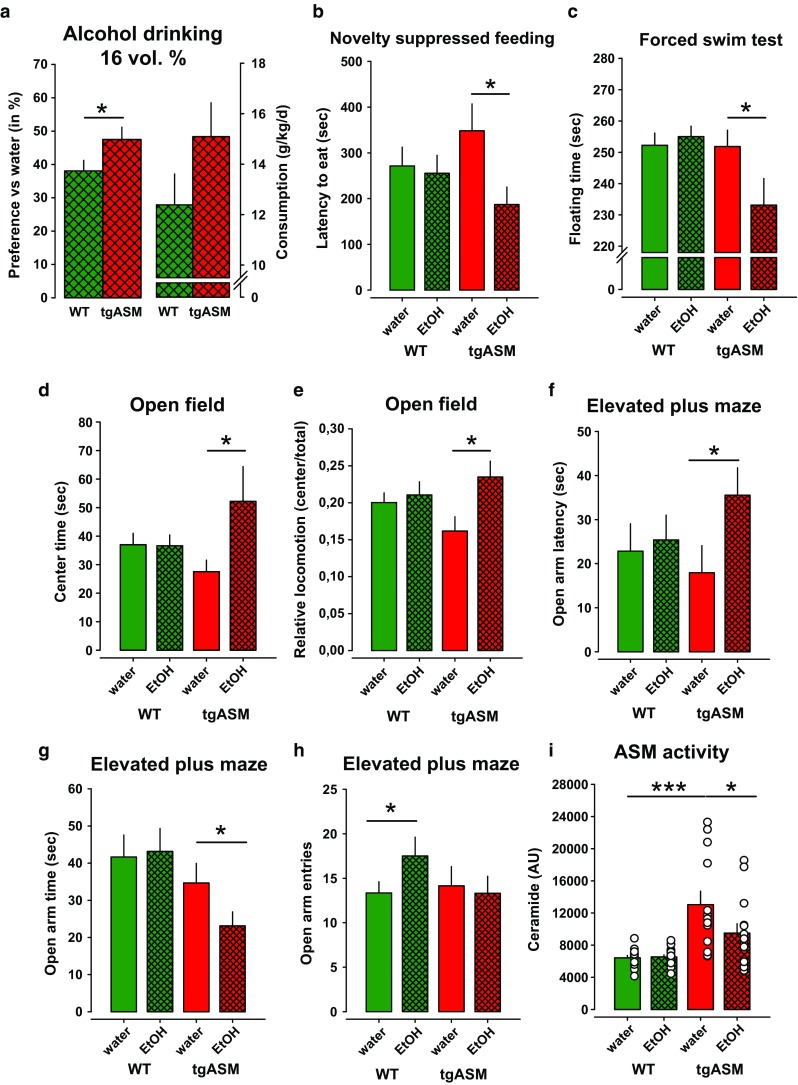
Free-choice alcohol drinking reduces depression-like behavior selectively in depressed mice. The *error bars* show the mean ± SEM. **a** Preference vs. water and absolute consumption of 16 vol% alcohol in mice over-expressing acid sphingomyelinase (tgASM, *n* = 16) and wild type (WT, *n* = 16) mice in a two-bottle free-choice drinking test. The animals were trained for consumption with increasing doses of alcohol and then remained on a 16 vol% solution during testing. The values present an average consumption of 4 days. There was a significant difference in preference at the target concentration of 16 vol% alcohol (*t* = −1.953; *p* = 0.03) and a trend for absolute consumption (*t* = −1.489, *p* = 0.07). **b** Alcohol drinking reduces depression-like behavior in the novelty suppressed feeding test selectively in tgASM mice (*t* = 2.305, *p* = 0.016; *n* = 13–16/group). **c** Alcohol drinking reduces depression-like behavior in the forced swim test selectively in tgASM mice (*t* = 1.878, *p* = 0.037). **d**, **e** The open field test showed a selective and temporally restricted anxiolytic-like effect of free-choice alcohol drinking in tgASM mice, which is only indicated by an increase in center time (*t* = −1.922, *p* = 0.047) and locomotion in the center of the maze (*t* = −2.516, *p* = 0.0135) in the first 5 min of testing. **f**–**h** In the elevated plus maze test for anxiety alcohol drinking enhanced anxiety-like behavior in tgASM mice, which is shown by prolonged latency to enter the open arms (*t* = −2.013, *p* = 0.027) and enhanced open arm time (*t* = 1.785, *p* = 0.044). In WT mice, alcohol drinking had a mild anxiolytic effect that was indicated by an increase in the number of open arm entries (*t* = −1.685, *p* = 0.05). **i** The ASM activity in the dorsal hippocampus was enhanced in tgASM mice compared to WT (*n* = 13–16/group; *t* = −3.918, *p* = 0.001). Free-choice alcohol drinking attenuated ASM activity in tgASM mice (*t* = 21.787, *p* = 0.047) (*p < 0.05, **p ≤ 0.01; ***p ≤ 0.001)

We found that enhanced ASM activity in mice led to depression [21] and enhanced alcohol consumption. However, this could be due to ASM being a common underlying factor for depression and alcohol drinking or a depression-AUD causality [6]. Hence, we asked whether alcohol consumption could reverse the depression-like phenotype and, therefore, serve as a self-medication. We tested naïve tgASM mice after establishing stable 16 vol% alcohol consumption in a two-bottle free-choice drinking test or after water drinking for their emotional phenotype. In this experiment, we replicated the enhanced alcohol consumption in tgASM compared to WT mice (Fig. 3a). We found that free-choice alcohol drinking significantly reduced the latency to eat in the novelty suppressed feeding test only in tgASM mice but not in WT mice (Fig. 3b). Additionally, in the forced swim test, alcohol had antidepressant effects only in tgASM but not in WT mice by reducing the time of floating (Fig. 3c). A correlation analysis revealed that the higher the amount of alcohol consumed by the tgASM mice was, the stronger the reduction was in floating behavior in this test (Pearson, *r* = −0.642, *p* = 0.01). No such correlation was found for WT mice (*r* = −0.139; *p* = 0.62; Suppl. Figure 3). In the open field, alcohol drinking enhanced the time spent in the center (Fig. 3d) and the locomotion in the center (Fig. 3e) only in tgASM mice. Alcohol drinking had no significant effect on total locomotion in the open field (*p* > 0.05), which suggests that the alcohol effects are not simply stimulant effects. Anxiety and depression are highly comorbid disorders but may be differentially regulated by sphingolipid systems [21, 51]. In this experiment, free-choice alcohol drinking enhanced anxiety levels in tgASM mice, as shown by an increase in the latency to enter the open arms (Fig. 3f) and a decline in the time spent on the open arms (Fig. 3g). In contrast, alcohol drinking slightly reduced anxiety in WT mice indicated by an increase of open arm entries (Fig. 3h). It is unlikely that the anxiety induced in tgASM mice is due to withdrawal effects [41] since the animals were tested with alcohol that was available before and after testing. Additionally, the alcohol drinking did not yield a gross locomotor phenotype, which could have explained the antidepressant or anxiogenic effects of the alcohol drinking in the tgASM mice [44]. To test how alcohol drinking would affect ASM activity, we measured ASM mediated SM-Cer turnover in the dorsal hippocampus after free-choice alcohol drinking. Enhanced ASM activity and subsequently increased ceramide levels in the dorsal hippocampus can induce depression in mice by inhibiting hippocampal neurogenesis [21]. In contrast, the dynamic down-regulation of dorsal hippocampus ASM activity was shown to predict re-learning during the extinction of an operant task [29]. Water-drinking tgASM mice showed ASM hyperactivity. Alcohol drinking had no effect on ASM activity in WT mice but instead normalized it in tgASM mice (Fig. 3i). ASM activity in the serum was enhanced in tgASM mice but was not affected by alcohol consumption (Suppl. Figure 4), thus suggesting that serum ASM is not a valid marker for the anti-depressant action of alcohol in the brains of depressed animals. Taken together, these findings suggest that free-choice alcohol drinking reduces depression-like behavior selectively in depressed animals but enhances anxiety levels. The anti-depressant effect of alcohol may be mediated by the normalization of ASM activity.

### No antidepressant effects from forced alcohol exposure

**Fig. 4:**
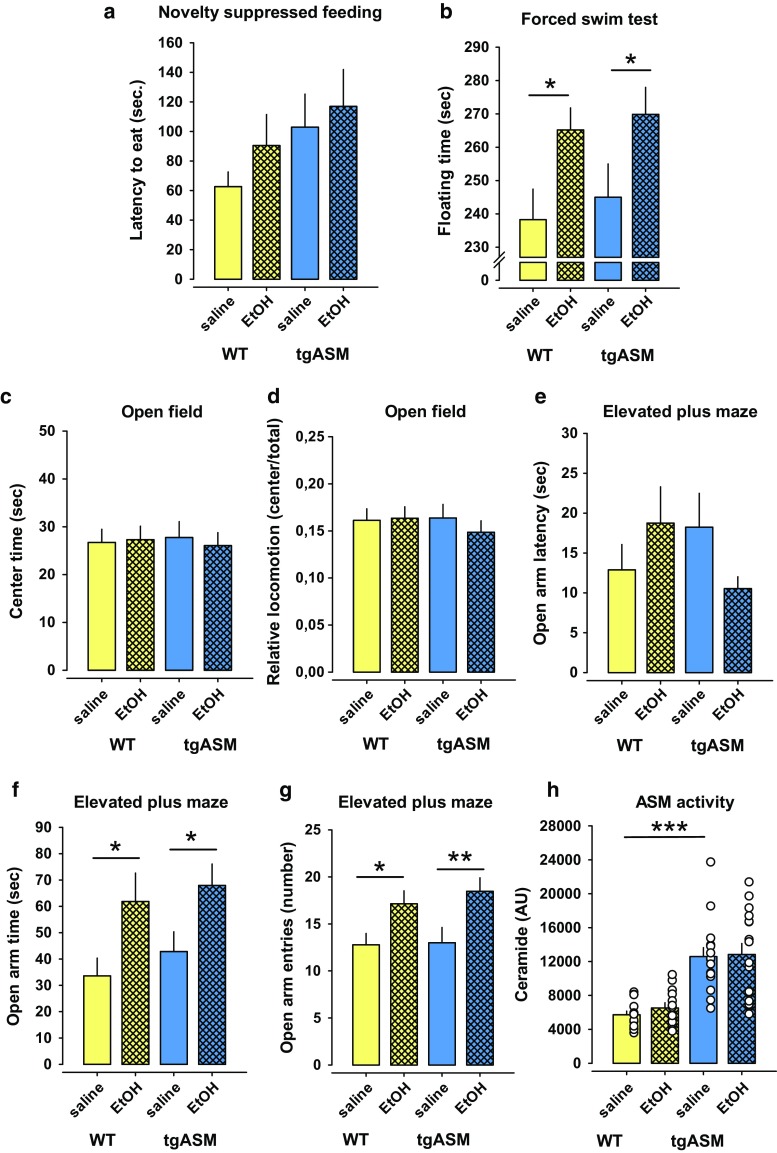
Forced alcohol exposure by repeated injection (2 g/kg; i.p.) has no anti-depressant effects on depression-like behavior in depressed mice, but non-specifically reduces anxiety. The *error bars* show the mean ± SEM. **a** Forced alcohol exposure has no effect on mice over-expressing acid sphingomyelinase (tgASM, *n* = 14–15/group) or wild type (WT, *n* = 12–13/group) mice in the novelty suppressed feeding test (*p* > 0.05). **b** Forced alcohol exposure enhances depression-like behavior in the forced swim test non-selectively in tgASM (*t* = −1.931, *p* = 0.031) as well as WT mice (*t* = −2.388, *p* = 0.012). **c**, **d** No effect of forced alcohol exposure in the open field test in tgASM as well as WT mice (*p* > 0.05) in first 5 min of testing (*p* > 0.05) was seen. **e**–**g** In the elevated plus maze test for anxiety, forced alcohol exposure reduced anxiety-like behavior non-selectively in tgASM as well as WT mice, which is shown by enhanced open arm time (tgASM: *t* = −2.277, *p* = 0.015; WT: *t* = −2.216, *p* = 0.018) and an increase in the number of open arm entries (tgASM: *t* = −2.521, *p* = 0.009; WT: *t* = −2.380, *p* = 0.012), but no significant effect on the latency to enter open arms (*p* > 0.05) was seen. **h** The ASM activity in plasma was enhanced in tgASM mice (*n* = 12–15/group) compared to WT (*n* = 13–14/group; *t* = −6.072, *p* < 0.001). Forced alcohol exposure had no effect on ASM activity in tgASM mice (*p* > 0.05) (*p < 0.05, **p < 0.01; ***p < 0.001)

It was suggested that the instrumentalization of alcohol as self-medication for depression requires individual self-titration and control over intake [47, 48]. Therefore, we asked whether the pharmacological reinforcing and mild euphoria-inducing action of alcohol [68] would suffice to exert the antidepressant and ASM normalizing effects. The animals were submitted to a forced alcohol exposure by receiving injections with either saline or alcohol (2 g/kg; i.p), which has been shown to induce reinforcing effects in the CPP tests. During this exposition, we tested their emotional behavior. In contrast to free-choice alcohol drinking, repeated forced exposure had no effects on the emotional behavior of the tgASM or WT mice in the novelty suppressed feeding- and open field test (Fig. 4a, c, d). In the forced swim test, forced alcohol exposure enhanced the floating time in WT and tgASM mice to a similar extent, which is suggestive of an increase in depression-like behavior (Fig. 4b). Interestingly, forced alcohol exposure also had the opposite effects on anxiety-related behavior compared to free-choice alcohol drinking. Alcohol injections had an unspecific anxiolytic effect in tgASM and WT mice, indicated by an increase in the time spent on the open arms and the number of entries while not significantly affecting the latency of entries to open arms (Fig. 4e–g). In contrast to free choice alcohol drinking, forced alcohol exposure did not reverse the enhanced ASM activity in the DH of tgASM mice (Fig. 4h).

We also tested hetKO ASM mice for the effects of repeated forced alcohol exposure on emotional behavior. Mice with innate attenuation of ASM activity showed less depression-like behavior and reduced anxiety in some, but not all tests (Suppl. Figure 5) [21]. Repeated forced alcohol exposure (2 g/kg; i.p.) had no significant effect in WT mice but it enhanced depression-like behavior in the novelty suppressed feeding- and forced swim test in hetKO ASM mice. Forced alcohol exposure also enhanced anxiety-like behavior in the elevated plus maze test in hetKO ASM mice but had no effect on ASM activity in the DH (Suppl. Figure 5).

Altogether, these findings suggest that the pharmacological and positively reinforcing action of alcohol is not sufficient to attenuate depression in depressed individuals, possibly because it fails to normalize enhanced ASM activity. These findings favor an interpretation that the self-titration of the pharmacological action of alcohol is essential for its instrumentalization as self-medication for depression.

### Alcohol drinking partially restores sphingomyelin levels in the nucleus accumbens but not in the dorsal hippocampus

**Fig. 5:**
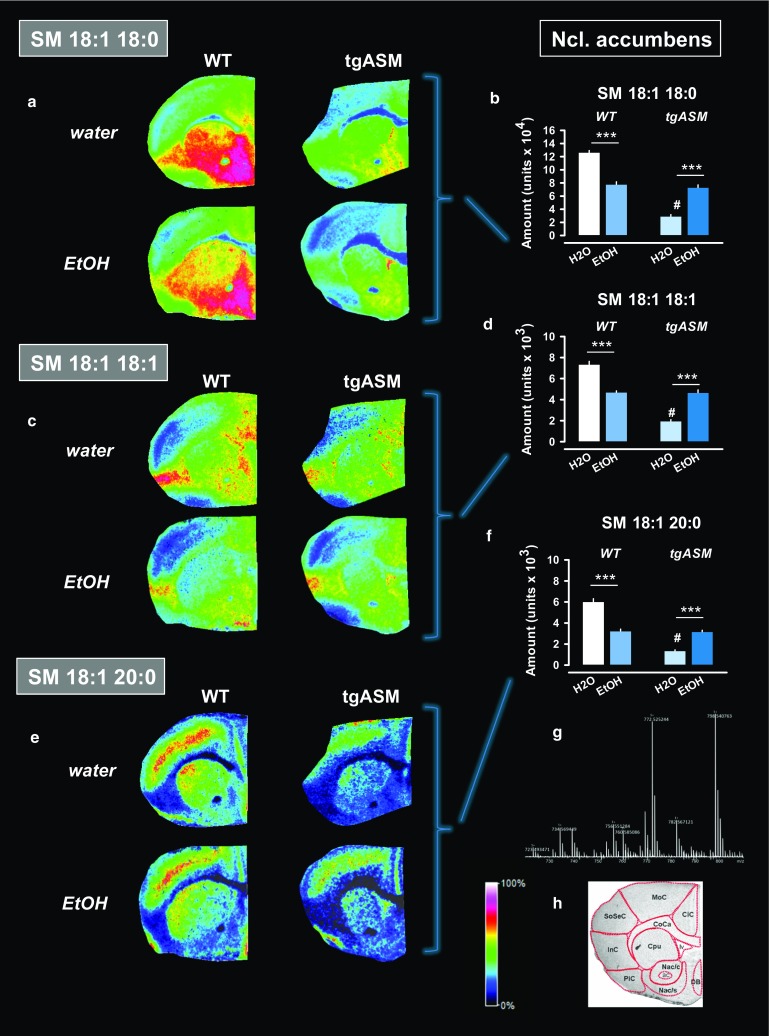
Free-choice alcohol drinking re-establishes sphingomyelin homeostasis in the nucleus accumbens (Nac) of mice over-expressing acid sphingomyelinase (tgASM). The mass spectrum generated in a MALDI-MS imaging contains hundreds of ion signals associated with each individual pixel. The MALDI images were acquired with *x*–*y*-raster widths of 30 µm for coronal sections. Slice MALDI mass spectrograms for the three most abundant sphingomyelin (SM) species in the neuropil of the Nac of water or 16 vol% alcohol (EtOH) drinking tgASM or wild type (WT) mice with regular consumption and three withdrawal and reinstatement episodes. The Nac was analyzed as a target area of interest. The *error bars* show a mean of nine samples/genotype-treatment (±SEM) for four animals. The data are shown using a rainbow scale, normalized against the total ion count. **a** The coronal tissue sections show SM 18:1 18:0 highly enriched in the Nac. **b** The abundance of SM 18:1 18:0 is largely reduced in tgASM mice (ANOVA, factor genotype: *F*_1,32_ = 240.793, *p* < 0.001). Alcohol drinking reduces levels of SM 18:1 18:0 in WT mice, but enhances it in tgASM mice (ANOVA, genotype × treatment interaction: *F*_1,32_ = 196.600, *p* < 0.001). **c** The coronal tissue sections show SM 18:1 18:1 that is highly enriched in the Nac and piriform cortex. **d** The abundance of SM 18:1 18:1 is largely reduced in tgASM mice (ANOVA, factor genotype: *F*_1,32_ = 165.064, *p* < 0.001). Alcohol drinking reduces levels of SM 18:1 18:1 in WT mice but enhances it in tgASM mice (ANOVA, genotype × treatment interaction: *F*_1,32_ = 161.448, *p* < 0.001). **e** The coronal tissue sections show SM 18:1 20:0 that is highly enriched in the somatosensory and motor cortex and in dorsolateral striatum. **f** The abundance of SM 18:1 20:0 is reduced in tgASM mice (ANOVA, factor genotype: *F*_1,32_ = 174.123, *p* < 0.001). Alcohol drinking reduces levels of SM 18:1 20:0 in WT mice but enhances it in tgASM mice (ANOVA, factor treatment: *F*_1,32_ = 7.292, *p* < 0.011; genotype × treatment interaction: *F*_1,32_ = 163.235, *p* < 0.001). **g** Mass spectrogram for a single slice at the Nac level with SM target masses. **h** Anatomical analysis of the slice at the level of the Nac (*ac* commisura anterior, *CiC* cingular cortex, *CoCa* corpus callosum, *CPu* caudate putamen, *DB* diagonal band of Broca, *lv* ventricle, *InC* insular cortex, *MoC* motor cortex, *Nac/c* Nac core, *Nac/s* Nac shell, *PiC* piriform cortex, *SoSeC* somatosensory cortex). For an overview on white matter and the liquor associated SM species, see Suppl. Figure 6, 7 (***p < 0.001; ^#^ *p* < 0.001 vs. WT-water)

ASM hyperactivity can induce depression by inducing a permanent sphingolipid allostasis in the brain. Many antidepressant drugs work, in addition to their action on monoamine systems, by exerting a functional inhibition of ASM [37, 38, 51]. Here, we explored how free-choice alcohol drinking affects sphingolipid homeostasis in the brain. We tested animals that had established stable 16 vol% alcohol consumption and underwent three withdrawal and reinstatement periods, which had boosted consumption, for the abundance of SM species in the nucleus accumbens (Nac) and dorsal hippocampus (DH). Solarix XR (Bruker Daltonik GmbH) FT- MALDI Imaging mass spectrometric measurements in brain slices revealed significantly attenuated levels of the three most abundant neuropil SM species, SM 18:1 18:0, SM 18:1 18:1, and SM 18:1 20:0, in tgASM mice in the Nac (Fig. 5; Suppl. Figure 6, 7), which most likely results from enhanced ASM-mediated turnover to Cer as suggested above. Alcohol drinking significantly reduced the abundance of all three SM species in the Nac in WT mice. Interestingly, the alcohol effects were reversed in tgASM mice in the Nac, where drinking significantly enhanced SM levels. This is in line with the above reported alcohol inhibition of ASM activity, and thus the reduced turnover of SM species.

**Fig. 6:**
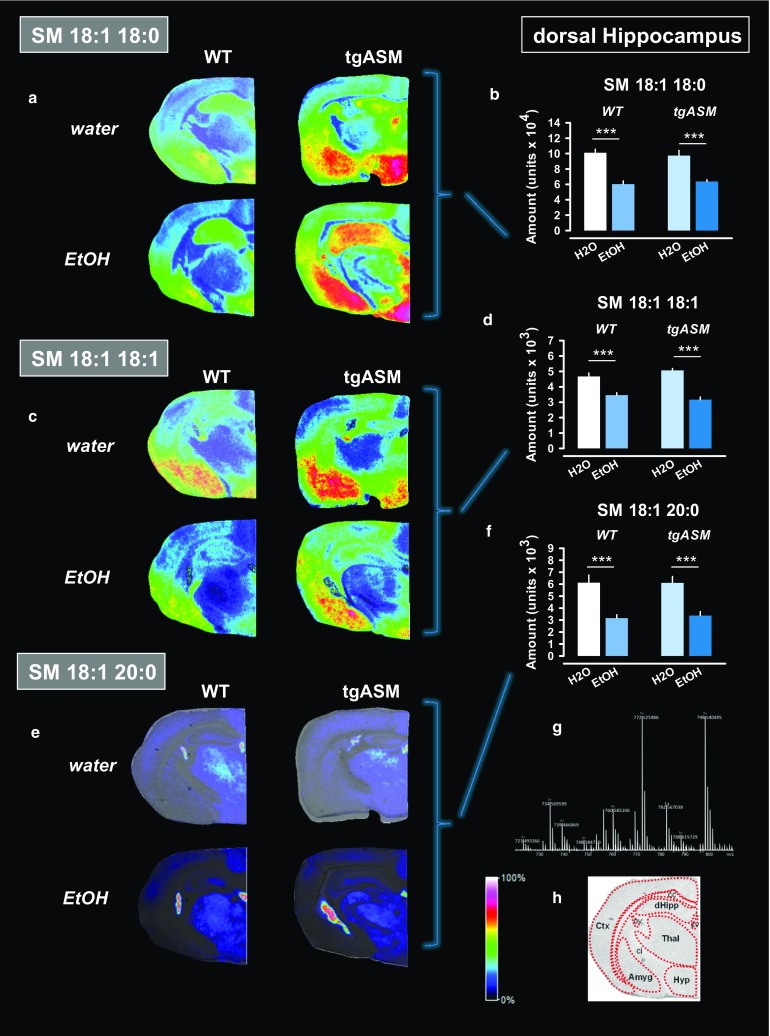
Free-choice alcohol drinking reduces sphingomyelin levels in the dorsal hippocampus (DH) of mice over-expressing acid sphingomyelinase (tgASM). The MALDI images were acquired with *x*–*y*-raster widths of 30 µm for coronal sections. Slice MALDI mass spectrograms for the three most abundant sphingomyelin (SM) species in the neuropil of the DH of water or 16 vol% alcohol (EtOH) drinking tgASM or wild type (WT) mice with regular consumption and three withdrawal and reinstatement episodes. The DH was analyzed as a target area of interest. The error*bars* show the mean of eight samples/genotype–treatment (±SEM) for four animals. The data are shown using a rainbow scale that is normalized against the total ion count. **a** The coronal tissue sections show that SM 18:1 18:0 is enriched in the hypothalamus, amygdala, and DH. **b** The abundance of SM 18:1 18:0 was not changed in tgASM mice (*p* > 0.05). Alcohol drinking reduces levels of SM 18:1 18:0 in WT and tgASM mice to the same degree (ANOVA, factor treatment: *F*_1,28_ = 63.281, *p* < 0.001). **c** The coronal tissue sections show that SM 18:1 18:1 is particularly enriched in the amygdala and hypothalamus. **d** The abundance of SM 18:1 18:1 was not changed in tgASM mice (*p* > 0.05). Alcohol drinking reduces levels of SM 18:1 18:1 in WT and tgASM mice to the same degree (ANOVA, factor treatment: *F*_1,28_ = 80.920, *p* < 0.001). **e** The coronal tissue sections show SM 18:1 20:0 enriched in the thalamus and ventricles. **f** The abundance of SM 18:1 20:0 was not changed in tgASM mice (*p* > 0.05). Alcohol drinking reduces levels of SM 18:1 20:0 in WT and tgASM mice to the same degree (ANOVA, factor treatment: *F*_1,28_ = 36.964, *p* < 0.001). **g** Mass spectrogram for a single slice point at the DH level with SM target masses. **h** Anatomical analysis of the slice at level of the Nac (*Amyg* amygdala, *cc* corpus callosum, *ci* commisura interior, *Ctx* cortex, *dHipp* dorsal hippocampus, *Hyp* hypothalamus, *Thal* thalamus). For an overview on white matter and liquor associated SM species, see Suppl. Figure 8, 9 (***p < 0.001)

The same SM species were measured in the DH (Fig. 6; Suppl. Figure 8, 9). In this structure, ASM hyperactivity did not lead to altered SM abundance. Alcohol drinking reduced levels of all three SM species, but in the same way in WT and tgASM mice. Altogether, these findings suggest that ASM hyperactivity induces a sphingolipid allostasis in the Nac but not DH. Free-choice alcohol drinking partially restores sphingolipid homeostasis in a brain region-selective way.

### Alcohol drinking restores monoamine levels in tgASM mice

**Fig. 7:**
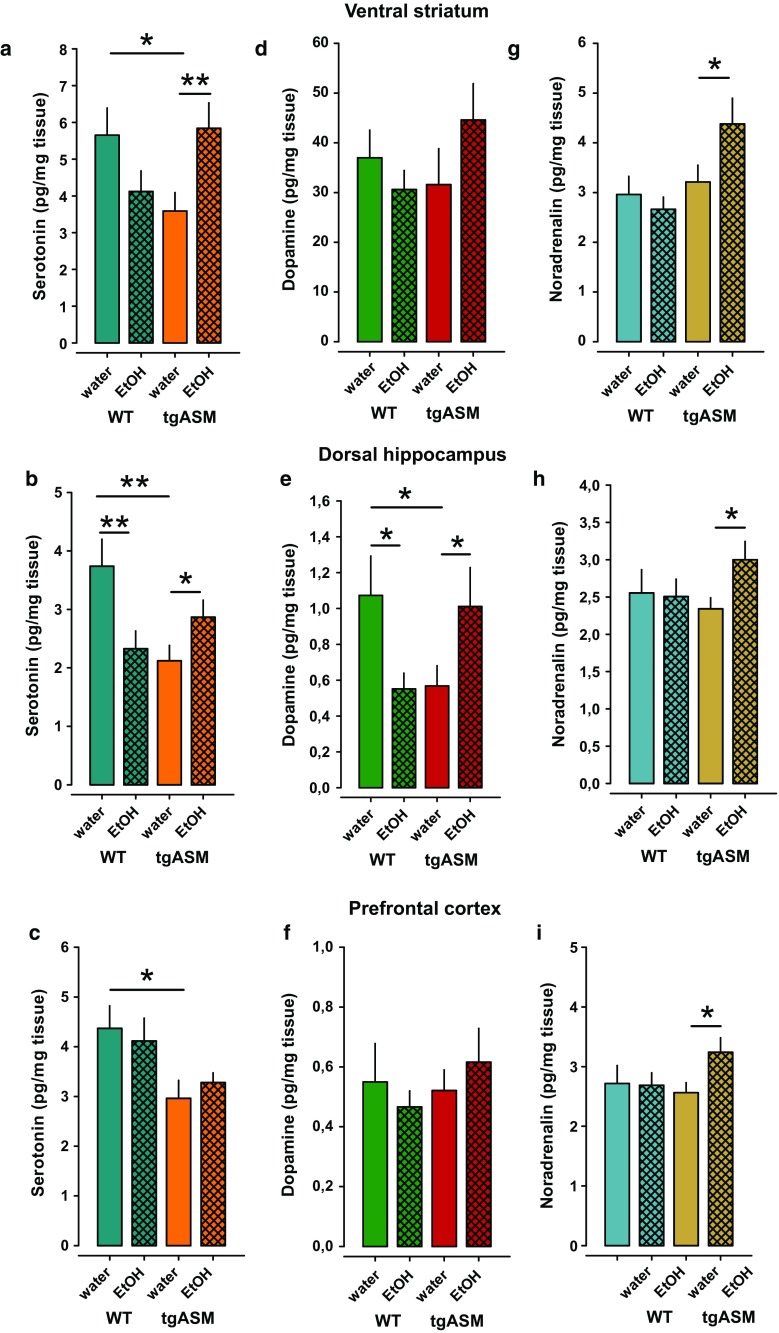
Free-choice alcohol drinking re-establishes monoamine tissue homeostasis in the ventral striatum (VS) and dorsal hippocampus (DH) of mice over-expressing acid sphingomyelinase (tgASM; *n* = 14–15/group). The *error bars* show the mean ± SEM. of monoamine concentrations in the wet tissue of water or 16 vol% alcohol drinking tgASM or wild type (WT, *n* = 14–16/group) mice. **a**–**c** The serotonin (5-HT) tissue levels are reduced in tgASM mice in the VS, DH, and prefrontal cortex PFC. Alcohol drinking reduces 5-HT levels in the DH and as a trend in the VS but has no effect in the PFC in WT mice. In contrast, in tgASM mice, alcohol drinking enhances 5-HT levels in the VS (ANOVA, genotype × treatment interaction: *F*_1,56_ = 8.627, *p* = 0.005) and in the DH (ANOVA, genotype × treatment interaction: *F*_1,55_ = 9.590, *p* = 0.003) but had no effect in the PFC (ANOVA, factor genotype: *F*_1,58_ = 8.170, *p* = 0.006). **d**–**f** The dopamine (DA) tissue levels are reduced in tgASM mice in the DH but not in the VS or PFC. Alcohol drinking reduces DA levels in the DH in WT mice. In contrast, in tgASM mice, alcohol drinking enhances DA levels in the DH (ANOVA, genotype × treatment interaction: *F*_1,54_ = 7.616, *p* = 0.008) but had no effect in the VS or PFC (*p* > 0.05). **g**–**i** Noradrenaline (NA) tissue levels are not affected by ASM over-expression in any of the brain structures investigated. Alcohol drinking had no effect on NA levels in WT mice but enhanced NA levels in tgASM mice in the VS, DH, and PFC, as shown by pre-planned comparisons (*p < 0.05, **p < 0.01; ***p < 0.001)

Depression is frequently associated with a disturbance of monoaminergic homeostasis in the brain [8, 39]. Here, we inquired as to how ASM hyperactivity would change monoamine homeostasis and whether alcohol drinking could reverse this. We established a stable consumption of water or 16 vol% alcohol in a free-choice alcohol drinking paradigm. After 12 days of stable drinking animals were sacrificed, and the tissue levels of serotonin (5-HT), dopamine (DA), and noradrenaline (NA) were measured in the ventral striatum (VS), DH and prefrontal cortex (PFC). In tgASM mice, we found a significant reduction in tissue 5-HT levels in the VS, DH, and PFC compared to WT (Fig. 7a–c). Alcohol drinking reversed this effect, and thus re-established 5-HT homeostasis in the VS and DH, but not in the PFC. ASM hyperactivity significantly reduced DA tissue levels in the DH but not in the VS or PFC (Fig. 7d–f). In the DH, alcohol drinking re-established DA homeostasis but had no effect on DA levels in the VS and PFC. NA tissue levels did not appear to be under the control of ASM in all the brain structures investigated (Fig. 7g–i). Nevertheless, alcohol drinking significantly enhanced NA tissue levels selectively in tgASM but not in WT mice in all three brain areas. Altogether, these findings show that alcohol drinking restored 5-HT- and DA tissue homeostasis only in animals with ASM hyperfunction, which may contribute to the anti-depressant effects.

### Alcohol and gene expression profiles in tgASM mice

**Fig. 8:**
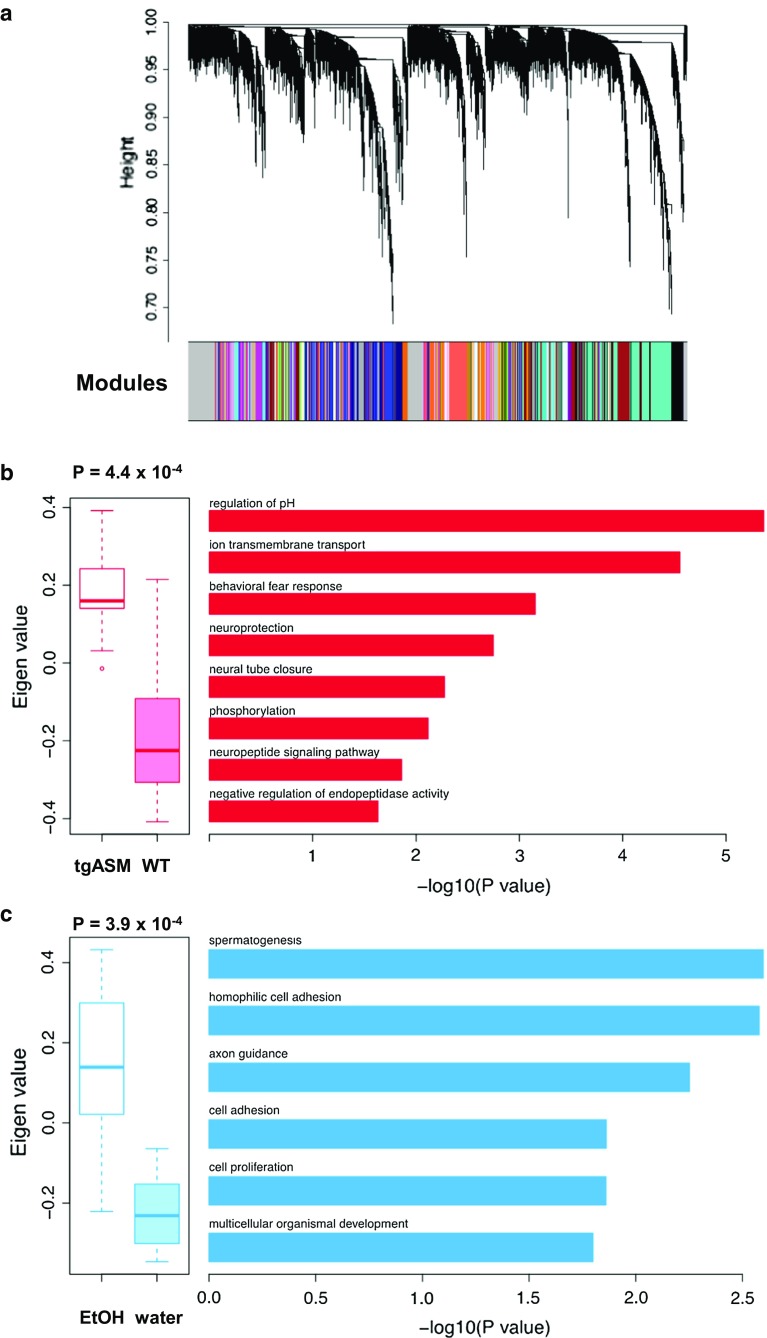
Acid sphingomyelinase (ASM) controls gene co-expression in the dorsal hippocampus (DH) of mice, a brain region that is essentially related to the ASM control of depression- and extinction-related behavior [21, 29]. **a** The co-expression network of ASM and alcohol. A cluster dendrogram generated by hierarchical clustering of genes on the basis of topological overlap. The modules of correlated genes were assigned colors and are indicated by the horizontal bar beneath the dendrogram, where all unassigned genes were placed in the gray module. **b** The functional enrichment analyses of the module associated with genotype status. The *boxplots* compare the module eigenvalues of transgenic ASM (TG) and wild-type (WT) genotype status. For genotype status comparisons, the *p* values are calculated using the full ANOVA model. The results of the functional enrichment analyses (BP: gene ontology biological process) for this module are represented in the bar plots. We used the hypergeometric test to calculate the *p* values and used the false discovery rate (FDR) of 5% to obtain significantly enriched BP terms. **c** The functional enrichment analyses of the module associated with treatment conditions. The boxplots compare the module eigenvalues of the active drinker (AD) and water- (WA) treated mice, and the *p* values are calculated using the full ANOVA model. The results of the functional enrichment analyses for this module are represented in the *bar plots*

Monoamine and sphingolipid homeostasis are both controlled by the activity of numerous proteins in the brain. To map the downstream effects of ASM hyperactivity and alcohol drinking, we tested animals that had established a stable 16 vol% alcohol consumption and underwent three withdrawal and reinstatement periods for gene expression in the DH using RNA-seq (tgASM-alcohol: *n* = 6; WT-alcohol: *n* = 5; tgASM-water: *n* = 3; WT-water: *n* = 4). The network analysis permits the identification of groups of genes (modules) with similar expression patterns across samples, which reflects shared biological functions and key functional pathways, as well as key hub genes within the module. From a total of 25,028 genes in the PFC, the network analysis identified a large number of modules (*n* = 88) with sizes ranging from 31 genes to 3378 genes (Fig. 8a). In this manner, the network analysis reduced thousands of genes across four conditions to a relatively small number of coherent gene modules that represent distinct transcriptional responses to either the overexpression in the murine ASM locus or alcohol treatment. We tested whether the expression of each module (summarized by its first principal component, the module eigengene) was associated with a genotype (tgASM vs. WT) or treatment (alcohol vs. water) or genotype × treatment interaction using an ANOVA model. We identified one module each for genotype (M1) and treatment (M2) that passed a false discover rate (FDR) threshold of 5% (Suppl. Tab. 2, 3). None of the interaction modules satisfied this criterion.

The module with strongest association with the genotype consisted of 78 genes and showed an increased eigengene profile in tgASM (Fig. 8b). The gene ontology (GO) enrichment analysis showed that genes related to regulation of pH (*Slc9a4*, *Slc9a2*, and *Slc26a4*), ion transmembrane transport (*Grik4*, *Kctd18*, *Slc9a4*, *Gabra5*, *Pkd1l3*, and *Slc26a4*), behavioral fear responses (*Npy2r* and *Gabra5*), neuroprotection (*Gabra5* and *Trim2*) and a neuropeptide signaling pathway (*Npy2r* and *Pkd1l3*) were significantly enriched in this module (Fig. 8b; Suppl. Tab. 2). In addition, several canonical pathways were significantly enriched in this module including neuroactive ligand-receptor interactions, metabolic pathways, and endocytosis (Suppl. Tab. 2). Interestingly, all genes in the module are up-regulated in tgASM when compared to WT mice and the top differentially expressed genes included the glutamate-gated ionic channel family gene, *Grik4* (log_2_ Fold Change, FC = 0.3, *p* = 5.10 × 10^−06^); the intracellular pH regulator, *Slc9a4* (FC = 0.83, *p* = 1.28 × 10^−05^); the guanine nucleotide exchange factor, *Dock9* (FC = 0.22, *p* = 1.69 × 10^−05^); the gene encoding glycoprotein, *Nell2* (FC = 0.22, *p* = 4.31 × 10^−05^); and the gene involved in central nervous system development, *Hapln1* (FC = 0.31 *p* = 1.54 × 10^−04^). *Grik4* encodes the kainic acid-type glutamate receptor 1 (KA1) subunit, which co-assembles with other glutamate receptor subunits to form cation-selective ion channels but may also possess metabotropic functions. Interestingly, human genetic studies have shown that variants in this gene were associated with major depressive disorder and alcoholism.

The module M2, the most significantly associated module with alcohol consumption, is comprised of 50 genes and showed an increased eigengene profile in active drinker (AD) mice compared to mice treated with water (Fig. 8c). The M2 is significantly enriched with genes involved in spermatogenesis (*Catsperg2*, *Ovol1*, and *Tbpl1*), cell adhesion (*Cdh7*, *Cass4*, and *Cdh4*), cell proliferation (*Srrt* and *Tyr*), and multicellular organismal development (*Catsperg2*, *Gap43*, *Utp3*, and *Fam3c*) (Suppl. Tab. 3). Several hub genes in this module showed a strong differential expression between AD and water-treated mice, including the calcium dependent cell–cell adhesion molecule, *Cdh7* (FC = 0.44, *p* = 7.28 × 10^−04^); a member of a family with sequence similarity 3 (FAM3), *Fam3c* (FC = 0.23, *p* = 3.5 × 10^−03^); and a gene encoding a protein that promotes adhesion between dendrites and axons, *Nxph1* (FC = 0.23, *p* = 4.75 × 10^−03^). Interestingly, genome-wide association studies in humans demonstrated that common variants in *Cdh7* were associated with bipolar disorder and major depressive disorder (MDD), indicating the comorbidity of MDD and alcohol. Altogether this analysis does not suggest a direct ASM regulation of cell membrane properties by controlling sphingolipid pathways. It rather suggests a regulation of cell homeostasis by controlling properties, such as pH, ion transmembrane transport, and neuroprotection-related genes. ASM hyperactivity leads to an over-expression of anxiety-related genes coding for the neuropeptide Y receptor Y2 (NPY-RY2) and gamma-aminobutyric acid A receptor, subunit alpha 5 (α5-GABA_A_-R). Neuropeptide Y (NPY) may exert potent anxiolytic effects through Y1 receptors but augments anxiety through Y2 receptors [71]. An enhanced α5-GABA_A_-R function was shown to be associated with anxiety in animals [7] and humans [26]. Both genes may, therefore, be additional mechanisms of the depressogenic effects of ASM hyperactivity [21].

### No evidence for the involvement of oxidative stress in alcohol’s antidepressant effects

While the ‘‘housekeeping’’ function of ASM in permanent SM turnover has been known for a long time, more recent results have defined the role of ASM in the cellular response to oxidative stress [31]. The activation of ASM, following psychosocial stress-triggered oxidative stress results in the generation of Cer [38]. Depression is associated with oxidative stress at the cellular level [45], which may in turn further enhance ASM activity. Antidepressant action may be at least partially mediated through a reduction of oxidative stress in the brain [67]. To investigate oxidative stress in tgASM mice and its potential reversal by alcohol, we measured the superoxide dismutase activity (SOD) of animals with established consumption of 16 vol% alcohol drinking immediately after drinking cessation and after one week of abstinence. Alcohol drinking did not affect SOD activity in the cerebellum of the WT or tgASM mice after drinking. Only after 7 days of abstinence from drinking there was an increase in tgASM mice (Suppl. Figure 10). These findings suggest that oxidative stress plays no role in ASM-induced depression and its reversal by alcohol drinking.

## Discussion

Neurobiological models of alcoholism assume that the direct positive and negative reinforcing action of alcohol maintains consumption, which is mediated by changes in the protein function of the brain [25, 35, 68]. This, so far, has neither led to effective prevention nor treatment of AUD. This may also not explain paradoxical action, such as anti-depressive effects. The expansion of the scope towards a systemic understanding may thus offer new approaches. Here, we show the first mechanism, to our knowledge, of effective alcohol instrumentalization, which is based on ASM action in the brain and an alcohol-induced re-establishment of sphingolipid homeostasis. We found that mice in which ASM hyperactivity caused a depressive phenotype drink significantly more alcohol than the WT controls. Alcohol drinking is also more susceptible to withdrawal effects. Alcohol conditioned reinforcing and locomotor effects are established faster in these mice, while the sedative effects are not affected. Thus, the anti-depressive effects of alcohol may not result from a sedating action. Mice with ASM hypoactivity showed slightly diminished alcohol consumption and a lack of alcohol’s conditioned reinforcing and locomotor sensitizing effects. This suggests that ASM controls alcohol consumption and alcohol reinforcement in both directions in parallel to emotional behavior [21]. Interestingly, free-choice alcohol consumption, which allows for self-titration, normalizes ASM activity in the brain and selectively reduces depression-like behavior in depressed tgASM mice. Forced alcohol exposure, in contrast, had no effect on brain ASM activity and instead enhanced depression-like behavior in tgASM and WT mice alike. It has to be admitted that due to the individual choice of the amount of alcohol consumed and the specific time of consumption, free-choice alcohol drinking cannot exactly be mimicked by forced alcohol exposure, where a medium dose of alcohol was given at a fixed time to each animal. However, both methods of alcohol exposure had been shown to have positive reinforcing action in mice, either in conditioned place preference [12] or by maintaining consumption behavior [11].

There is a well-known correlative link between the anxiety and depression traits of an organism and its responsiveness to alcohol [3, 23]. Numerous studies have shown that forced exposure, e.g., by injection, as well as the free-choice consumption of alcohol can have acute anxiolytic and antidepressant effects, which may last up to 24 h [4, 74]. The effects of forced exposure, however, wear off with repeated exposure. This reversal was suggested to be based on a switch from excitatory to the inhibitory action of alcohol on mesocortico-limbic monoamine responses after chronic exposure [33, 50]. We found mild anxiolytic effects in WT mice after several weeks of free-choice alcohol consumption and strong anxiolytic effects after acute alcohol injection in the EPM test. Since the EPM was tested after only two injections in a series of five sub-chronic alcohol injections, this may account for the rather acute effects of the test. Chronic alcohol exposure during free drinking did not affect depression-like behavior in several tests. However, repeated alcohol injections increased depression-like behavior in WT and tgASM mice in the FST. At the time of the testing the animals had received a total of five alcohol injections in the forced exposure treatment. Following this, our findings confirm the previous data on the rather complex time course in the emotional effects of acute and chronic forced alcohol exposure. In this study, free-choice alcohol consumption—but not sub-chronic forced exposure—reduced depression-like behavior in depressed tgASM mice. The selective anti-depressive effects of chronic free-choice alcohol consumption have also been reported in animals with a high susceptibility to chronic mild stress which was related to an opioidergic mechanism [62]. Overall, the present studies suggest that the anti-depressant effects of alcohol do not only depend on its pharmacological action but also on the emotional state of the organism and the free-access and self-titration opportunity.

In WT mice, free-choice alcohol drinking reduced the tissue levels of the most abundant SM species in the Nac and DH of mice. This is in line with the reduction of plasma SM species in alcohol dependent patients [60]. Previous studies also reported a corresponding decrease in Cer species in the cortex and forebrain after alcohol binge drinking in mice [2] and rats [18], that was not mediated by the altered activity of ASM but by reduced activity of sphingolipid delta(4)-desaturase, an enzyme involved in Cer *de novo* synthesis [18]. In mice with ASM hyperactivity that show already attenuated levels of these SM species, alcohol drinking had the opposite effect and partially reversed the SM decline, thus re-establishing SM homeostasis. These findings are in line with a previous observation that a functional inhibition of the ASM would yield an antidepressant effect [21, 38]. Interestingly, this effect was brain-region specific and did not occur in the DH. These findings further support the notion of a brain-region specific fine tuning of sphingolipid activity during behavioral adaptations and their emotional correlates [29, 53].

Depression is associated with the dysregulation of monoaminergic activity [8, 39]. Here, we show that depression induced by ASM hyperactivity coincides with reduced tissue levels of 5-HT and DA, but not NA, in several brain structures. 5-HT and DA deficits are partially reversed by alcohol drinking in depressed mice, with no effect in WT mice. These findings suggest that re-establishing sphingolipid homeostasis has downstream effects and also normalizes the 5-HT and DA function preferentially in the VS and DH.

An interesting observation was the region specific effect of free-choice alcohol drinking on sphingolipid allostasis. This study reported three different types of sphingolipid expressions in the brain: (1) gray matter-associated (Figs. 5, 6), (2) white matter-associated (Suppl. Figure 6 and 8), and (3) liquor-associated (Suppl. Figure 7 and 9) SM species. Among the gray matter-associated sphingolipids, there was a brain region specific distribution of SM and Cer species [2, 29], which is mediated by local sphingolipid catabolizing enzymes, such as ASM [29]. This may also suggests, that sphingolipid homeostasis is regulated at the local tissue level. The Nac is compared to, e.g., the DH, a brain region with strong monoaminergic innervation (e.g., Fig. 7). In light of the observed link between ASM activity and monoamine tissue levels and the well-known effect of alcohol on dopamine [68] and serotonin [50] activity in the Nac, the Nac may appear to be a brain region that is exclusively sensitive to a sphingolipid-dopamine/serotonin interaction, and thus the alcohol effects on sphingolipid allostasis.

A limitation of this study is that the present model of a sphingolipid-driven depression does not account for all types of depression observed. Depression is a heterogeneous disorder with numerous genetic and environmental risk factors leading to distinct pathways and rather unique behavioral and somatic symptoms [38, 39]. Therefore, the suggested mechanism of depression-induced alcohol abuse may only apply to a subpopulation of depressed patients, characterized by high innate or induced ASM activity [36]. For these patients, the present findings may offer a new target for diagnostic and individualized treatment. Whether other pathogenic pathways into a depression use a similar pathway to induce alcohol abuse behavior needs to be addressed in future studies.

Notably, alcohol is not a treatment for depression and, given its neurotoxic action and neuropathological effects at higher doses, it should never be one. However, the clinical reality in the western world, where approximately 30% of depressed patients with co-morbid AUD engage in alcohol consumption to self-medicate for their depression, should not be denied. Therefore, our findings do not suggest alcohol as a new treatment for depression but focus on what alcohol does in these patients: it normalizes a lipid imbalance in the brain. Herein, the present findings suggest a new class of molecules (i.e., the sphingolipids) and pathways (their controlling enzyme cascades) for the understanding and treatment of affective disorders-AUD comorbidity.

We propose that alcohol drinking can have paradoxical antidepressant effects in depressed organisms. This is mediated by an alcohol-induced restoration of ASM activity and subsequently of sphingolipid- and monoamine homeostasis. This may provide the first mechanism for alcohol instrumentalization with the goal to self-medicate for depressive symptoms.

## Electronic supplementary material

Below is the link to the electronic supplementary material.

Supplementary material 1 (PPT 13446 kb)

## References

[1] Anders S, Pyl PT, Huber W (2015). HTSeq—a Python framework to work with high-throughput sequencing data. Bioinformatics.

[2] Bae M, Bandaru VV, Patel N, Haughey NJ (2014). Ceramide metabolism analysis in a model of binge drinking reveals both neuroprotective and toxic effects of ethanol. J Neurochem.

[3] Bahi A (2013). Individual differences in elevated plus-maze exploration predicted higher ethanol consumption and preference in outbred mice. Pharmacol Biochem Behav.

[4] Barkley-Levenson AM, Crabbe JC (2015). Genotypic and sex differences in anxiety-like behavior and alcohol-induced anxiolysis in High Drinking in the Dark selected mice. Alcohol.

[5] Baum-Baicker C (1985). The psychological benefits of moderate alcohol consumption: a review of the literature. Drug Alcohol Depend.

[6] Boden JM, Fergusson DM (2011). Alcohol and depression. Addiction.

[7] Botta P, Demmou L, Kasugai Y, Markovic M, Xu C, Fadok JP (2015). Regulating anxiety with extrasynaptic inhibition. Nat Neurosci.

[8] Carr GV, Lucki I, Müller CP, Jacobs BL (2010). The Role of Serotonin in Depression. Handbook of the behavioral neurobiology of serotonin.

[9] Dobin A, Davis CA, Schlesinger F, Drenkow J, Zaleski C, Jha S (2013). STAR: ultrafast universal RNA-seq aligner. Bioinformatics.

[10] Easton AC, Lucchesi W, Schumann G, Peter GK, Müller CP, Fernandes C (2011). alphaCaMKII autophosphorylation controls exploratory activity to threatening novel stimuli. Neuropharmacology.

[11] Easton AC, Lucchesi W, Lourdusamy A, Lenz B, Solati J, Golub Y (2013). αCaMKII autophosphorylation controls the establishment of alcohol drinking behavior. Neuropsychopharmacology.

[12] Easton AC, Lucchesi W, Mizuno K, Fernandes C, Schumann G, Giese KP (2013). alphaCaMKII autophosphorylation controls the establishment of alcohol-induced conditioned place preference in mice. Behav Brain Res.

[13] EMCDDA (2012) European Monitoring Center for Addictive Drugs, State of the drug problem 2012. http://www.emcdda.europa.eu/publications/annual-report/2012. Accessed 20 June 2013

[14] Fantini J, Barrantes FJ (2009). Sphingolipid/cholesterol regulation of neurotransmitter receptor conformation and function. Biochim Biophys Acta.

[15] Franklin KBJ, Paxionos G (1997). The mouse brain in stereotaxic coordinates.

[16] Garcia-Valdecasas-Campelo E, Gonzalez-Reimers E, Santolaria-Fernandez F, De La Vega-Prieto MJ, Milena-Abril A, Sanchez-Perez MJ (2007). Brain atrophy in alcoholics: relationship with alcohol intake; liver disease; nutritional status, and inflammation. Alcohol Alcohol.

[17] Gilpin NW, Koob GF (2008). Neurobiology of alcohol dependence: focus on motivational mechanisms. Alcohol Res Health.

[18] Godfrey J, Jeanguenin L, Castro N, Olney JJ, Dudley J, Pipkin J (2015). Chronic voluntary ethanol consumption induces favorable ceramide profiles in selectively bred alcohol-preferring (P) rats. PLoS One.

[19] Grant BF, Harford TC (1995). Comorbidity between DSM-IV alcohol use disorders and major depression: results of a national survey. Drug Alcohol Depend.

[20] Gulbins E, Kolesnick R (2003). Raft ceramide in molecular medicine. Oncogene.

[21] Gulbins E, Palmada M, Reichel M, Lüth A, Böhmer C, Amato D (2013). Acid sphingomyelinase-ceramide system mediates effects of antidepressant drugs. Nature Med.

[22] Hannun YA, Obeid LM (2008). Principles of bioactive lipid signalling: lessons from sphingolipids. Nature Rev Mol Cell Biol.

[23] Hayton SJ, Mahoney MK, Olmstead MC (2012). Behavioral traits predicting alcohol drinking in outbred rats: an investigation of anxiety, novelty seeking, and cognitive flexibility. Alcohol Clin Exp Res.

[24] Heath DB (2000). Drinking occasions: comparative perspectives on alcohol and culture.

[25] Heilig M, Koob GF (2007). A key role for corticotropin-releasing factor in alcohol dependence. Trends Neurosci.

[26] Hodges LM, Fyer AJ, Weissman MM, Logue MW, Haghighi F, Evgrafov O (2014). Evidence for linkage and association of GABRB3 and GABRA5 to panic disorder. Neuropsychopharmacology.

[27] Horinouchi K, Erlich S, Perl DP, Ferlinz K, Bisgaier CL, Sandhoff K (1995). Acid sphingomyelinase deficient mice: a model of types A and B Niemann–Pick disease. Nature Genet.

[28] Huston JP, Silva MA, Topic B, Müller CP (2013). What’s conditioned in conditioned place preference?. Trends Pharmacol Sci.

[29] Huston JP, Kornhuber J, Mühle C, Japtok L, Komorowski M, Mattern C (2016). A sphingolipid mechanism for behavioral extinction. J Neurochem.

[30] Jain M, Ngoy S, Sheth SA, Swanson RA, Rhee EP, Liao R (2014). Systematic survey of lipids across mouse tissues. Am J Physiol Endocrinol Metab.

[31] Jenkins RW, Canals D, Hannun YA (2009). Roles and regulation of secretory and lysosomal acid sphingomyelinase. Cell Signal.

[32] Jung ME, Metzger DB (2016). A sex difference in oxidative stress and behavioral suppression induced by ethanol withdrawal in rats. Behav Brain Res.

[33] Karkhanis AN, Huggins KN, Rose JH, Jones SR (2016). Switch from excitatory to inhibitory actions of ethanol on dopamine levels after chronic exposure: role of kappa opioid receptors. Neuropharmacology.

[34] Kippin TE (2011). Does drug mis-instrumentalization lead to drug abuse?. Behav Brain Sci.

[35] Koob GF, Le Moal M (2008). Addiction and the brain antireward system. Annu Rev Psychol.

[36] Kornhuber J, Medlin A, Bleich S, Jendrossek V, Henkel AW, Wiltfang J (2005). High activity of acid sphingomyelinase in major depression. J Neural Transm.

[37] Kornhuber J, Tripal P, Reichel M, Mühle C, Rhein C, Muehlbacher M (2010). Functional Inhibitors of Acid Sphingomyelinase (FIASMAs): a novel pharmacological group of drugs with broad clinical applications. Cell Physiol Biochem.

[38] Kornhuber J, Müller CP, Becker KA, Reichel M, Gulbins E (2014). The ceramide system as a novel antidepressant target. Trends Pharmacol Sci.

[39] Krishnan V, Nestler EJ (2008). The molecular neurobiology of depression. Nature.

[40] Ledesma MD, Prinetti A, Sonnino S, Schuchman EH (2011). Brain pathology in Niemann Pick disease type A: insights from the acid sphingomyelinase knockout mice. J Neurochem.

[41] Lee KM, Coehlo M, McGregor HA, Waltermire RS, Szumlinski KK (2015). Binge alcohol drinking elicits persistent negative affect in mice. Behav Brain Res.

[42] Lenz B, Müller CP, Stoessel C, Sperling W, Biermann T, Hillemacher T (2012). Sex hormone activity in alcohol addiction: integrating organizational and activational effects. Prog Neurobiol.

[43] Love MI, Huber W, Anders S (2014). Moderated estimation of fold change and dispersion for RNA-seq data with DESeq2. Genome Biol.

[44] Lovinger DM, Crabbe JC (2005). Laboratory models of alcoholism: treatment target identification and insight into mechanisms. Nat Neurosci.

[45] Michel TM, Frangou S, Thiemeyer D, Camara S, Jecel J, Nara K (2007). Evidence for oxidative stress in the frontal cortex in patients with recurrent depressive disorder—a postmortem study. Psychiatry Res.

[46] Miquel M, Vazquez-Sanroman D, Carbo-Gas M, Gil-Miravet I, Sanchis-Segura C, Carulli D (2016). Have we been ignoring the elephant in the room? Seven arguments for considering the cerebellum as part of addiction circuitry. Neurosci Biobehav Rev.

[47] Müller CP, Schumann G (2011). Drugs as instruments: a new framework for non-addictive psychoactive drug use. Behav Brain Sci.

[48] Müller CP, Schumann G (2011). To use or not to use: expanding the view on non-addictive psychoactive drug consumption and its implications. Behav Brain Sci.

[49] Müller CP (2013). Episodic memories and their relevance for psychoactive drug use and addiction. Front Behav Neurosci.

[50] Müller CP, Homberg JR (2015). The role of serotonin in drug use and addiction. Behav Brain Res.

[51] Müller CP, Reichel M, Mühle C, Rhein C, Gulbins E, Kornhuber J (2015). Brain membrane lipids in major depression and anxiety disorders. Biochim Biophys Acta.

[52] Nuber S, Harmuth F, Kohl Z, Adame A, Trejo M, Schönig K (2013). A progressive dopaminergic phenotype associated with neurotoxic conversion of α-synuclein in BAC-transgenic rats. Brain.

[53] Oliveira TG, Chan RB, Bravo FV, Miranda A, Silva RR, Zhou B (2016). The impact of chronic stress on the rat brain lipidome. Mol Psychiatry.

[54] Peele S, Brodsky A (2000). Exploring psychological benefits associated with moderate alcohol use: a necessary corrective to assessments of drinking outcomes?. Drug Alcohol Depend.

[55] Pettinati HM, O’Brien CP, Dundon WD (2013). Current status of co-occurring mood and substance use disorders: a new therapeutic target. Am J Psychiatry.

[56] Poklis A, Mackell MA (1982). Evaluation of a modified alcohol dehydrogenase assay for the determination of ethanol in blood. Clin Chem.

[57] Pum ME, Carey RJ, Huston JP, Müller CP (2008). Role of medial prefrontal, entorhinal, and occipital 5-HT in cocaine-induced place preference and hyperlocomotion: evidence for multiple dissociations. Psychopharmacology.

[58] Reichel M, Greiner E, Richter-Schmidinger T, Yedibela O, Tripal P, Jacobi A (2010). Increased acid sphingomyelinase activity in peripheral blood cells of acutely intoxicated patients with alcohol dependence. Alcohol Clin Exp Res.

[59] Reichel M, Beck J, Mühle C, Rotter A, Bleich S, Gulbins E (2011). Activity of secretory sphingomyelinase is increased in plasma of alcohol-dependent patients. Alcohol Clin Exp Res.

[60] Reichel M, Honig S, Liebisch G, Luth A, Kleuser B, Gulbins E (2015). Alterations of plasma glycerophospholipid and sphingolipid species in male alcohol-dependent patients. Biochim Biophys Acta.

[61] Robbins TW, Ersche KD, Everitt BJ (2008). Drug addiction and the memory systems of the brain. Ann N Y Acad Sci.

[62] Sacharczuk M, Juszczak G, Swiergiel AH, Jaszczak K, Lipkowski AW, Sadowski B (2009). Alcohol reverses depressive and pronociceptive effects of chronic stress in mice with enhanced activity of the opioid system. Acta Neurobiol Exp (Wars).

[63] SAMHSA (2011) National survey on drug use and health, Substance Abuse and Mental Health Services Administration, 2011. Rockville http://www.samhsa.gov/data/NSDUH/ 011SummNatFindDetTables/Index.aspx). Accessed 20 June 2013

[64] Schuckit MA, Tipp JE, Bergman M, Reich W, Hesselbrock VM, Smith TL (1997). Comparison of induced and independent major depressive disorders in 2,945 alcoholics. Am J Psychiatry.

[65] Schwarz E, Prabakaran S, Whitfield P, Major H, Leweke FM, Koethe D (2008). High throughput lipidomic profiling of schizophrenia and bipolar disorder brain tissue reveals alterations of free fatty acids, phosphatidylcholines, and ceramides. J Proteome Res.

[66] Shariatgorji M, Nilsson A, Goodwin RJ, Kallback P, Schintu N, Zhang X (2014). Direct targeted quantitative molecular imaging of neurotransmitters in brain tissue sections. Neuron.

[67] Smaga I, Pomierny B, Krzyzanowska W, Pomierny-Chamiolo L, Miszkiel J, Niedzielska E (2012). *N*-acetylcysteine possesses antidepressant-like activity through reduction of oxidative stress: behavioral and biochemical analyses in rats. Prog Neuropsychopharmacol Biol Psychiatry.

[68] Spanagel R (2009). Alcoholism: a systems approach from molecular physiology to addictive behavior. Physiol Rev.

[69] Stacey D, Bilbao A, Maroteaux M, Jia T, Easton AC, Longueville S (2012). RASGRF2 regulates alcohol-induced reinforcement by influencing mesolimbic dopamine neuron activity and dopamine release. Proc Natl Acad Sci USA.

[70] Süß P, Kalinichenko L, Baum W, Reichel M, Kornhuber J, Loskarn S (2015). Hippocampal structure and function are maintained despite severe innate peripheral inflammation. Brain Behav Immun.

[71] Tasan RO, Nguyen NK, Weger S, Sartori SB, Singewald N, Heilbronn R (2010). The central and basolateral amygdala are critical sites of neuropeptide Y/Y2 receptor-mediated regulation of anxiety and depression. J Neurosci.

[72] Veiga MP, Goñi FM, Alonso A, Marsh D (2000). Mixed membranes of sphingolipids and glycerolipids as studied by spin-label ESR spectroscopy. A search for domain formation. Biochemistry.

[73] Vengeliene V, Bilbao A, Molander A, Spanagel R (2008). Neuropharmacology of alcohol addiction. Br J Pharmacol.

[74] Wolfe SA, Workman ER, Heaney CF, Niere F, Namjoshi S, Cacheaux LP (2016). FMRP regulates an ethanol-dependent shift in GABABR function and expression with rapid antidepressant properties. Nat Commun.

[75] Zheng F, Puppel A, Huber SE, Link AS, Eulenburg V, van Brederode JF (2016). Activin controls ethanol potentiation of Inhibitory syn aptic transmission through GABA receptors and concomitant behavioral sedation. Neuropsychopharmacology.

