# Supplementary material for: Paradoxical antidepressant effects of alcohol are related to acid sphingomyelinase and its control of sphingolipid homeostasis
Source: Acta Neuropathol. 2016 Dec 20;133(3):463–83. doi: 10.1007/s00401-016-1658-6 (PMC5325869; doi:10.1007/s00401-016-1658-6)
Supplement: Supplementary file 1 — Supplementary material 1 (PPT 13446 kb) [file 401_2016_1658_MOESM1_ESM.ppt]

## Slide 1
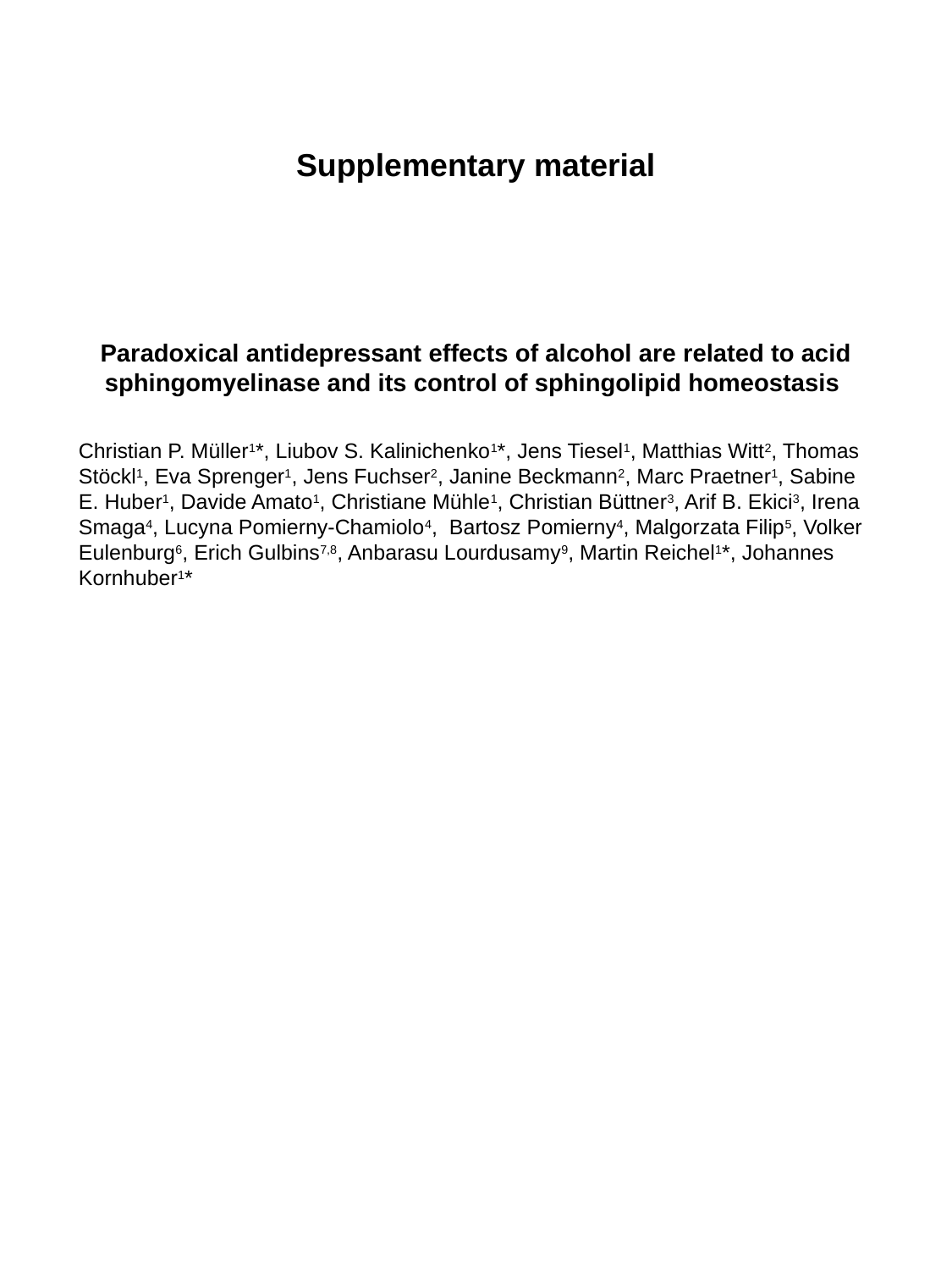

Supplementary material
Paradoxical antidepressant effects of alcohol are related to acid sphingomyelinase and its control of sphingolipid homeostasis
Christian P. Müller1*, Liubov S. Kalinichenko1*, Jens Tiesel1, Matthias Witt2, Thomas Stöckl1, Eva Sprenger1, Jens Fuchser2, Janine Beckmann2, Marc Praetner1, Sabine E. Huber1, Davide Amato1, Christiane Mühle1, Christian Büttner3, Arif B. Ekici3, Irena Smaga4, Lucyna Pomierny-Chamiolo4, Bartosz Pomierny4, Malgorzata Filip5, Volker Eulenburg6, Erich Gulbins7,8, Anbarasu Lourdusamy9, Martin Reichel1*, Johannes Kornhuber1*

## Slide 2
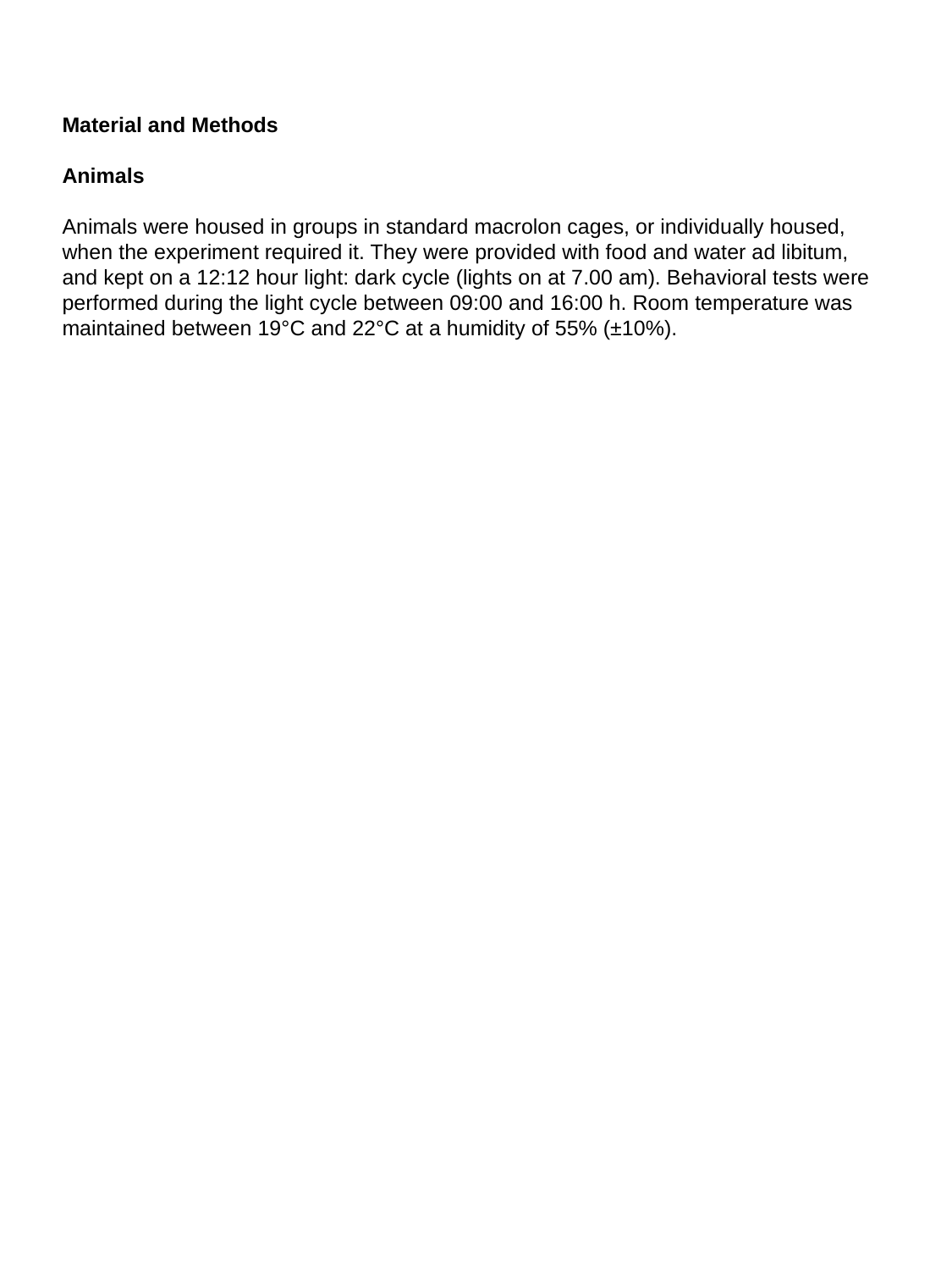

Material and Methods
Animals
Animals were housed in groups in standard macrolon cages, or individually housed, when the experiment required it. They were provided with food and water ad libitum, and kept on a 12:12 hour light: dark cycle (lights on at 7.00 am). Behavioral tests were performed during the light cycle between 09:00 and 16:00 h. Room temperature was maintained between 19°C and 22°C at a humidity of 55% (±10%).

## Slide 3
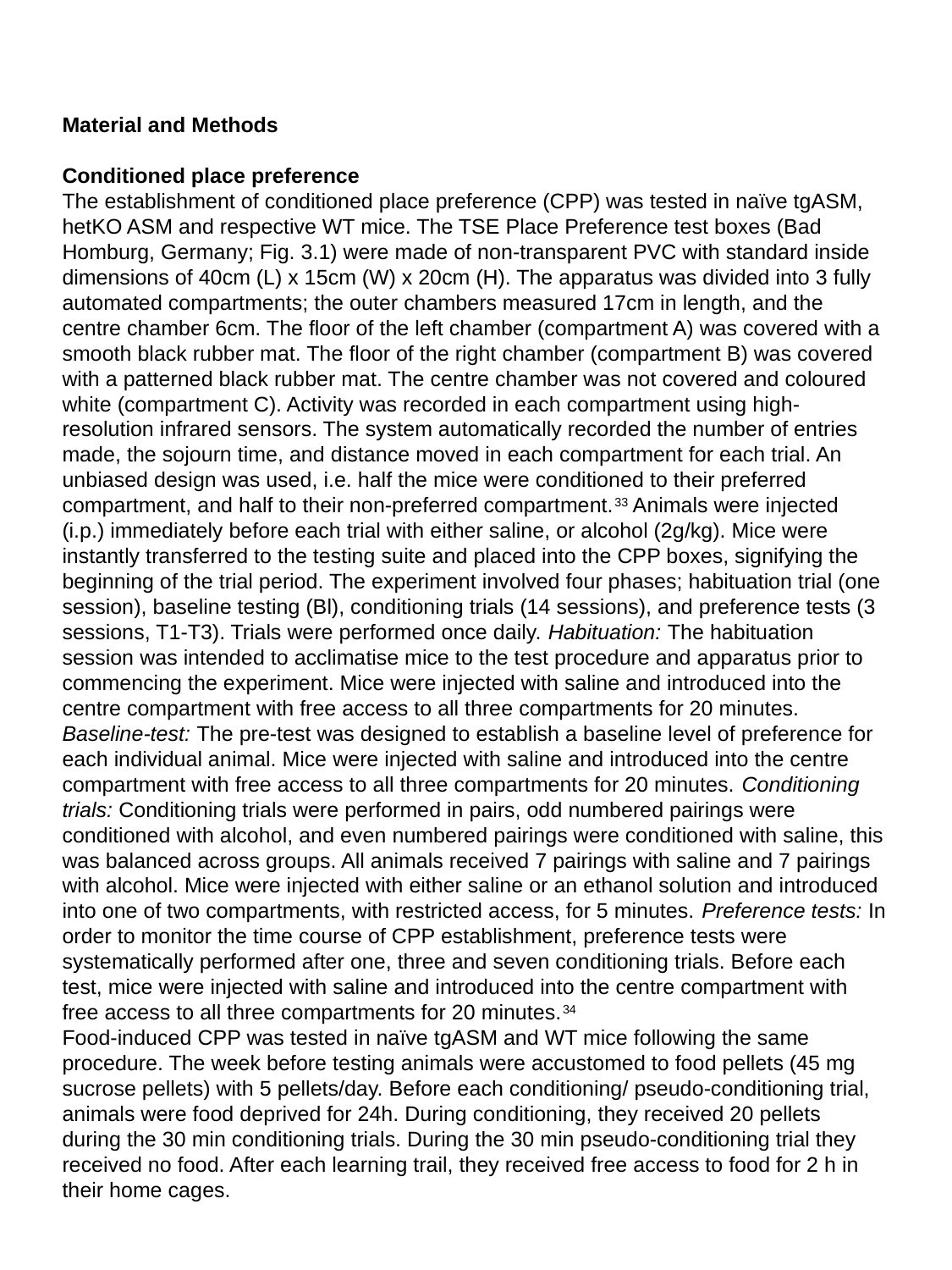

Material and Methods
Conditioned place preference
The establishment of conditioned place preference (CPP) was tested in naïve tgASM, hetKO ASM and respective WT mice. The TSE Place Preference test boxes (Bad Homburg, Germany; Fig. 3.1) were made of non-transparent PVC with standard inside dimensions of 40cm (L) x 15cm (W) x 20cm (H). The apparatus was divided into 3 fully automated compartments; the outer chambers measured 17cm in length, and the centre chamber 6cm. The floor of the left chamber (compartment A) was covered with a smooth black rubber mat. The floor of the right chamber (compartment B) was covered with a patterned black rubber mat. The centre chamber was not covered and coloured white (compartment C). Activity was recorded in each compartment using high-resolution infrared sensors. The system automatically recorded the number of entries made, the sojourn time, and distance moved in each compartment for each trial. An unbiased design was used, i.e. half the mice were conditioned to their preferred compartment, and half to their non-preferred compartment.33 Animals were injected (i.p.) immediately before each trial with either saline, or alcohol (2g/kg). Mice were instantly transferred to the testing suite and placed into the CPP boxes, signifying the beginning of the trial period. The experiment involved four phases; habituation trial (one session), baseline testing (Bl), conditioning trials (14 sessions), and preference tests (3 sessions, T1-T3). Trials were performed once daily. Habituation: The habituation session was intended to acclimatise mice to the test procedure and apparatus prior to commencing the experiment. Mice were injected with saline and introduced into the centre compartment with free access to all three compartments for 20 minutes. Baseline-test: The pre-test was designed to establish a baseline level of preference for each individual animal. Mice were injected with saline and introduced into the centre compartment with free access to all three compartments for 20 minutes. Conditioning trials: Conditioning trials were performed in pairs, odd numbered pairings were conditioned with alcohol, and even numbered pairings were conditioned with saline, this was balanced across groups. All animals received 7 pairings with saline and 7 pairings with alcohol. Mice were injected with either saline or an ethanol solution and introduced into one of two compartments, with restricted access, for 5 minutes. Preference tests: In order to monitor the time course of CPP establishment, preference tests were systematically performed after one, three and seven conditioning trials. Before each test, mice were injected with saline and introduced into the centre compartment with free access to all three compartments for 20 minutes.34
Food-induced CPP was tested in naïve tgASM and WT mice following the same procedure. The week before testing animals were accustomed to food pellets (45 mg sucrose pellets) with 5 pellets/day. Before each conditioning/ pseudo-conditioning trial, animals were food deprived for 24h. During conditioning, they received 20 pellets during the 30 min conditioning trials. During the 30 min pseudo-conditioning trial they received no food. After each learning trail, they received free access to food for 2 h in their home cages.

## Slide 4
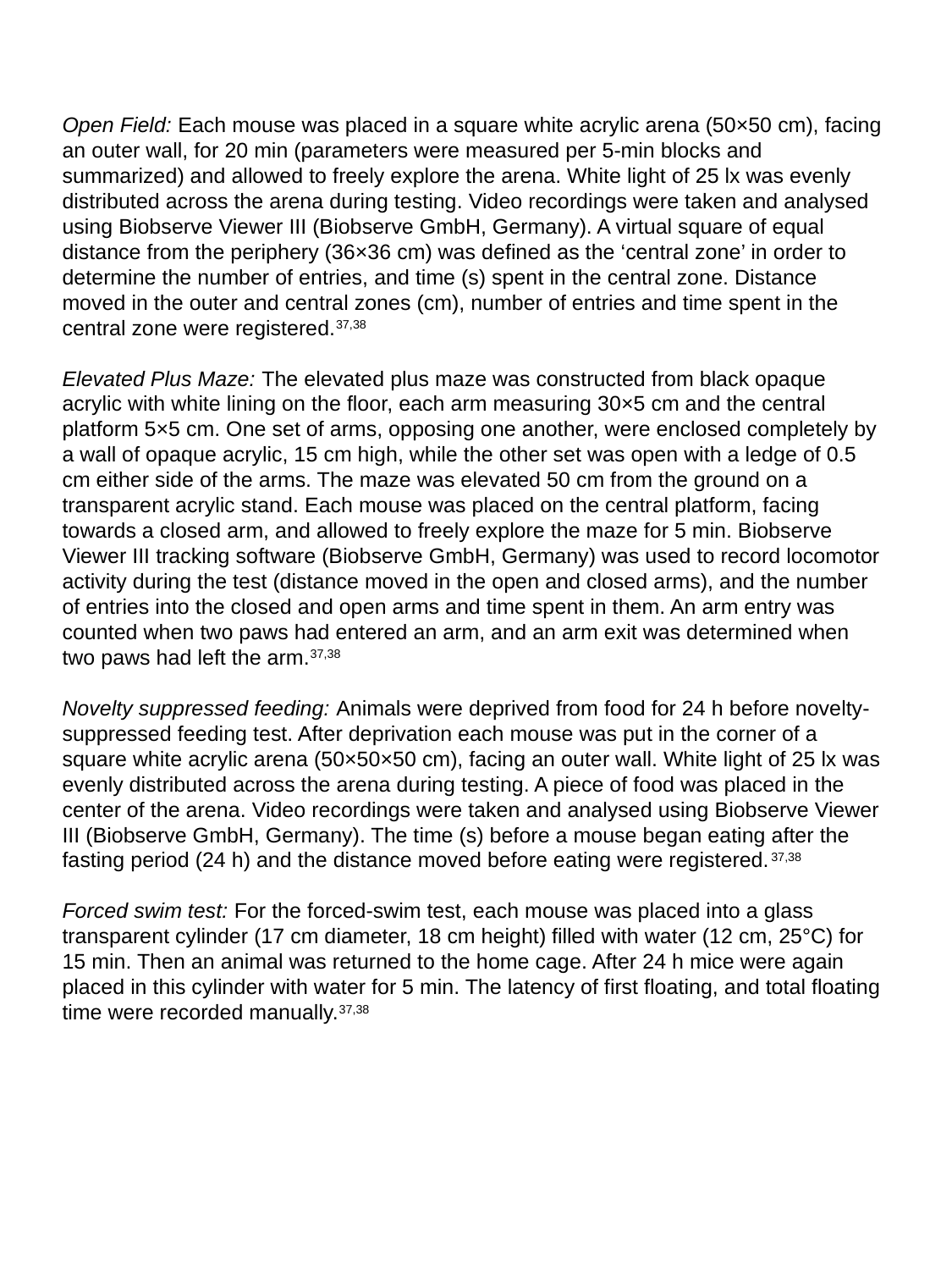

Open Field: Each mouse was placed in a square white acrylic arena (50×50 cm), facing an outer wall, for 20 min (parameters were measured per 5-min blocks and summarized) and allowed to freely explore the arena. White light of 25 lx was evenly distributed across the arena during testing. Video recordings were taken and analysed using Biobserve Viewer III (Biobserve GmbH, Germany). A virtual square of equal distance from the periphery (36×36 cm) was defined as the ‘central zone’ in order to determine the number of entries, and time (s) spent in the central zone. Distance moved in the outer and central zones (cm), number of entries and time spent in the central zone were registered.37,38
Elevated Plus Maze: The elevated plus maze was constructed from black opaque acrylic with white lining on the floor, each arm measuring 30×5 cm and the central platform 5×5 cm. One set of arms, opposing one another, were enclosed completely by a wall of opaque acrylic, 15 cm high, while the other set was open with a ledge of 0.5 cm either side of the arms. The maze was elevated 50 cm from the ground on a transparent acrylic stand. Each mouse was placed on the central platform, facing towards a closed arm, and allowed to freely explore the maze for 5 min. Biobserve Viewer III tracking software (Biobserve GmbH, Germany) was used to record locomotor activity during the test (distance moved in the open and closed arms), and the number of entries into the closed and open arms and time spent in them. An arm entry was counted when two paws had entered an arm, and an arm exit was determined when two paws had left the arm.37,38
Novelty suppressed feeding: Animals were deprived from food for 24 h before novelty-suppressed feeding test. After deprivation each mouse was put in the corner of a square white acrylic arena (50×50×50 cm), facing an outer wall. White light of 25 lx was evenly distributed across the arena during testing. A piece of food was placed in the center of the arena. Video recordings were taken and analysed using Biobserve Viewer III (Biobserve GmbH, Germany). The time (s) before a mouse began eating after the fasting period (24 h) and the distance moved before eating were registered.37,38
Forced swim test: For the forced-swim test, each mouse was placed into a glass transparent cylinder (17 cm diameter, 18 cm height) filled with water (12 cm, 25°C) for 15 min. Then an animal was returned to the home cage. After 24 h mice were again placed in this cylinder with water for 5 min. The latency of first floating, and total floating time were recorded manually.37,38

## Slide 5
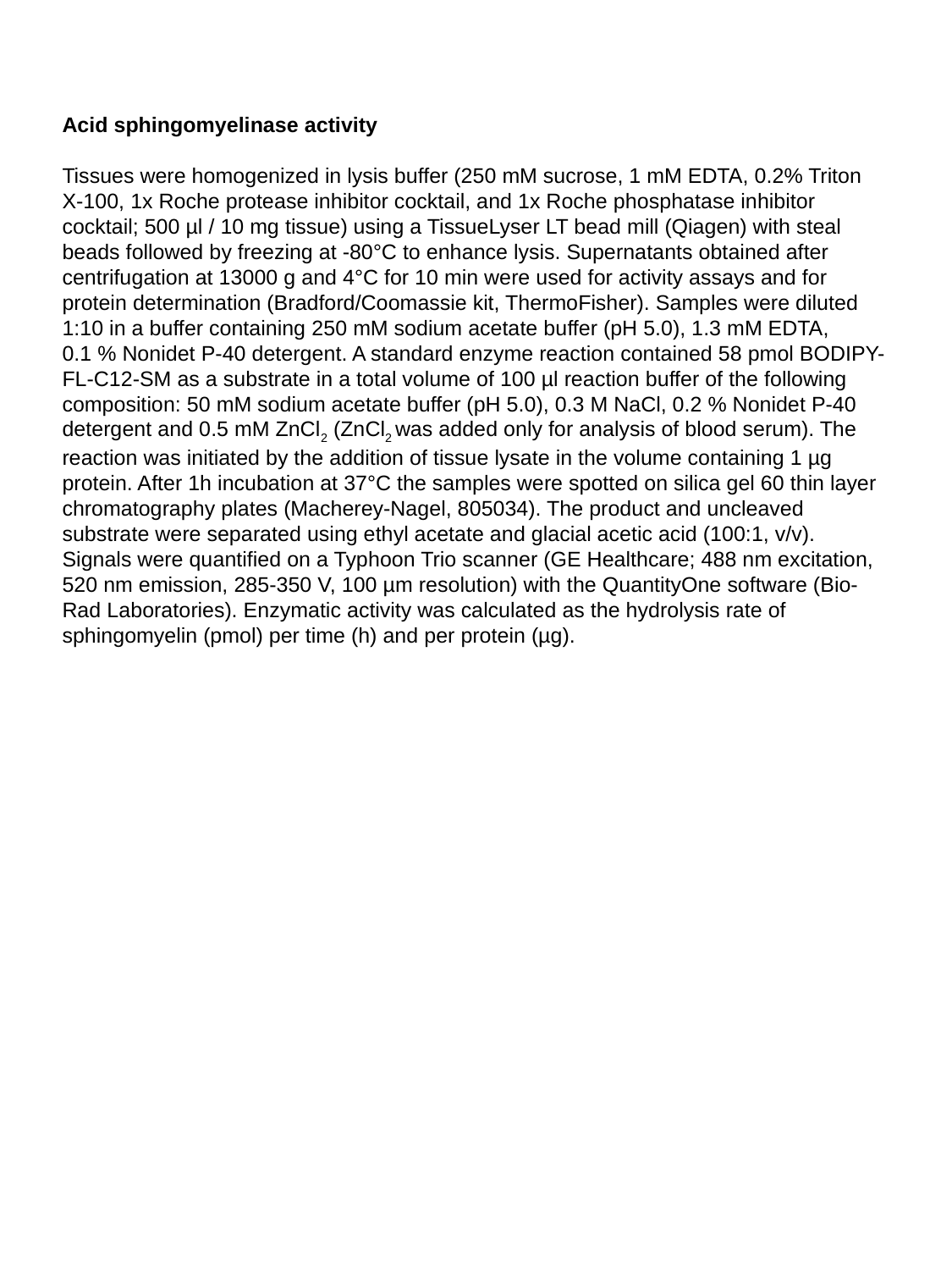

Acid sphingomyelinase activity
Tissues were homogenized in lysis buffer (250 mM sucrose, 1 mM EDTA, 0.2% Triton X-100, 1x Roche protease inhibitor cocktail, and 1x Roche phosphatase inhibitor cocktail; 500 µl / 10 mg tissue) using a TissueLyser LT bead mill (Qiagen) with steal beads followed by freezing at -80°C to enhance lysis. Supernatants obtained after centrifugation at 13000 g and 4°C for 10 min were used for activity assays and for protein determination (Bradford/Coomassie kit, ThermoFisher). Samples were diluted 1:10 in a buffer containing 250 mM sodium acetate buffer (pH 5.0), 1.3 mM EDTA, 0.1 % Nonidet P-40 detergent. A standard enzyme reaction contained 58 pmol BODIPY-FL-C12-SM as a substrate in a total volume of 100 µl reaction buffer of the following composition: 50 mM sodium acetate buffer (pH 5.0), 0.3 M NaCl, 0.2 % Nonidet P-40 detergent and 0.5 mM ZnCl2 (ZnCl2 was added only for analysis of blood serum). The reaction was initiated by the addition of tissue lysate in the volume containing 1 µg protein. After 1h incubation at 37°C the samples were spotted on silica gel 60 thin layer chromatography plates (Macherey-Nagel, 805034). The product and uncleaved substrate were separated using ethyl acetate and glacial acetic acid (100:1, v/v). Signals were quantified on a Typhoon Trio scanner (GE Healthcare; 488 nm excitation, 520 nm emission, 285-350 V, 100 µm resolution) with the QuantityOne software (Bio-Rad Laboratories). Enzymatic activity was calculated as the hydrolysis rate of sphingomyelin (pmol) per time (h) and per protein (µg).

## Slide 6
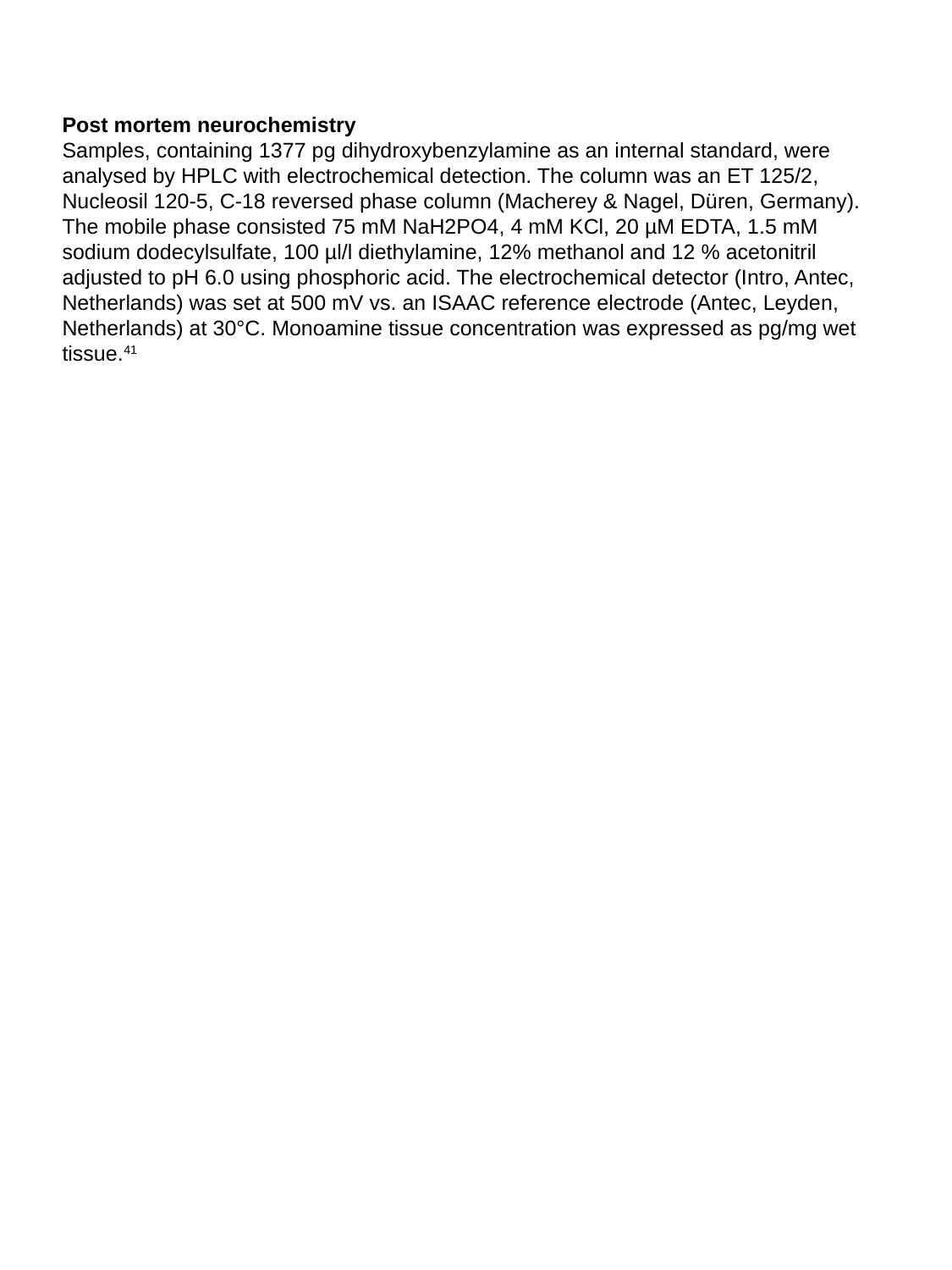

Post mortem neurochemistry
Samples, containing 1377 pg dihydroxybenzylamine as an internal standard, were analysed by HPLC with electrochemical detection. The column was an ET 125/2, Nucleosil 120-5, C-18 reversed phase column (Macherey & Nagel, Düren, Germany). The mobile phase consisted 75 mM NaH2PO4, 4 mM KCl, 20 µM EDTA, 1.5 mM sodium dodecylsulfate, 100 µl/l diethylamine, 12% methanol and 12 % acetonitril adjusted to pH 6.0 using phosphoric acid. The electrochemical detector (Intro, Antec, Netherlands) was set at 500 mV vs. an ISAAC reference electrode (Antec, Leyden, Netherlands) at 30°C. Monoamine tissue concentration was expressed as pg/mg wet tissue.41

## Slide 7
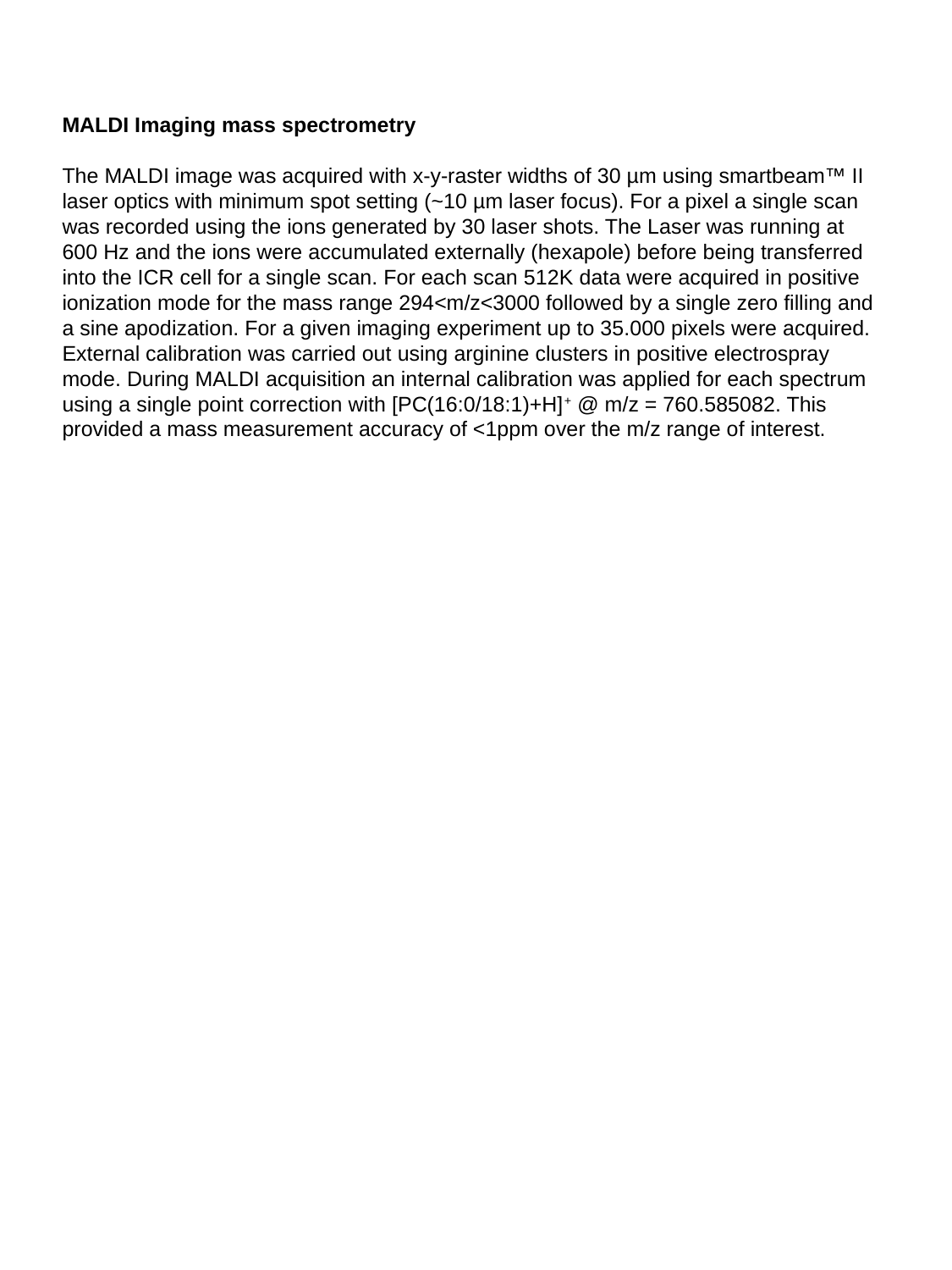

MALDI Imaging mass spectrometry
The MALDI image was acquired with x-y-raster widths of 30 µm using smartbeam™ II laser optics with minimum spot setting (~10 µm laser focus). For a pixel a single scan was recorded using the ions generated by 30 laser shots. The Laser was running at 600 Hz and the ions were accumulated externally (hexapole) before being transferred into the ICR cell for a single scan. For each scan 512K data were acquired in positive ionization mode for the mass range 294<m/z<3000 followed by a single zero filling and a sine apodization. For a given imaging experiment up to 35.000 pixels were acquired. External calibration was carried out using arginine clusters in positive electrospray mode. During MALDI acquisition an internal calibration was applied for each spectrum using a single point correction with [PC(16:0/18:1)+H]+ @ m/z = 760.585082. This provided a mass measurement accuracy of <1ppm over the m/z range of interest.

## Slide 8
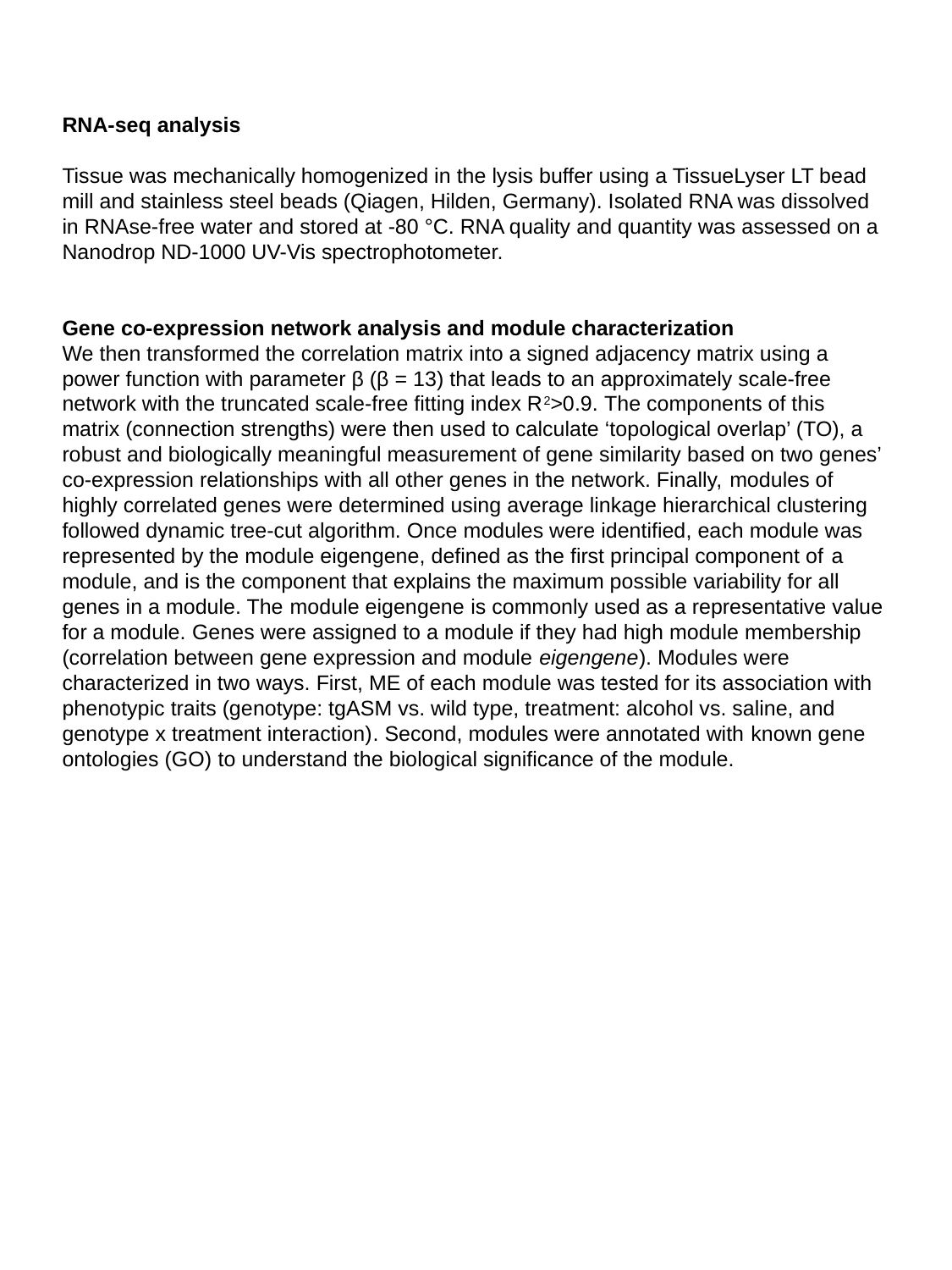

RNA-seq analysis
Tissue was mechanically homogenized in the lysis buffer using a TissueLyser LT bead mill and stainless steel beads (Qiagen, Hilden, Germany). Isolated RNA was dissolved in RNAse-free water and stored at -80 °C. RNA quality and quantity was assessed on a Nanodrop ND-1000 UV-Vis spectrophotometer.
Gene co-expression network analysis and module characterization
We then transformed the correlation matrix into a signed adjacency matrix using a power function with parameter β (β = 13) that leads to an approximately scale-free network with the truncated scale-free fitting index R2>0.9. The components of this matrix (connection strengths) were then used to calculate ‘topological overlap’ (TO), a robust and biologically meaningful measurement of gene similarity based on two genes’ co-expression relationships with all other genes in the network. Finally, modules of highly correlated genes were determined using average linkage hierarchical clustering followed dynamic tree-cut algorithm. Once modules were identified, each module was represented by the module eigengene, defined as the first principal component of a module, and is the component that explains the maximum possible variability for all genes in a module. The module eigengene is commonly used as a representative value for a module. Genes were assigned to a module if they had high module membership (correlation between gene expression and module eigengene). Modules were characterized in two ways. First, ME of each module was tested for its association with phenotypic traits (genotype: tgASM vs. wild type, treatment: alcohol vs. saline, and genotype x treatment interaction). Second, modules were annotated with known gene ontologies (GO) to understand the biological significance of the module.

## Slide 9
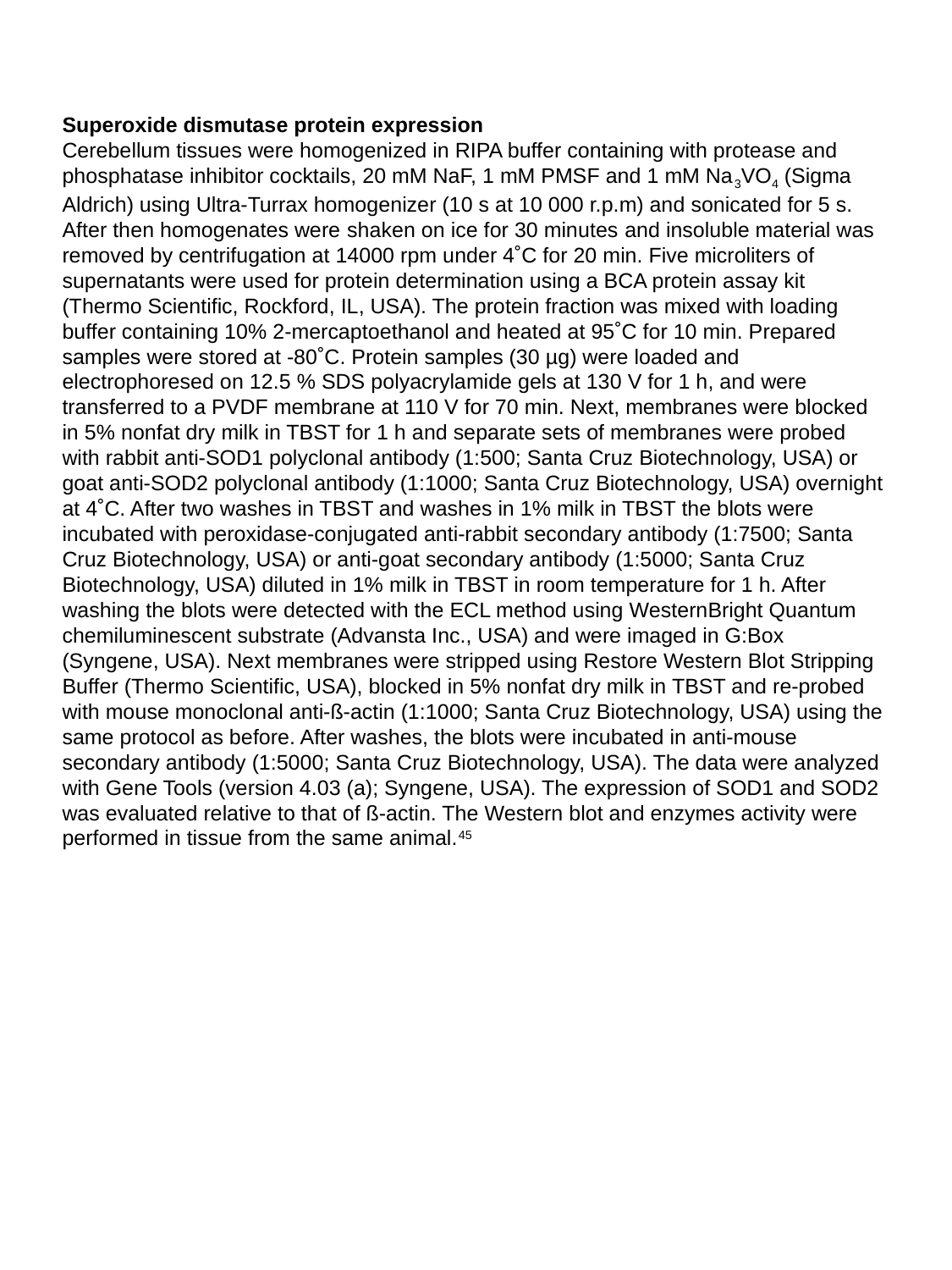

Superoxide dismutase protein expression
Cerebellum tissues were homogenized in RIPA buffer containing with protease and phosphatase inhibitor cocktails, 20 mM NaF, 1 mM PMSF and 1 mM Na3VO4 (Sigma Aldrich) using Ultra-Turrax homogenizer (10 s at 10 000 r.p.m) and sonicated for 5 s. After then homogenates were shaken on ice for 30 minutes and insoluble material was removed by centrifugation at 14000 rpm under 4˚C for 20 min. Five microliters of supernatants were used for protein determination using a BCA protein assay kit (Thermo Scientific, Rockford, IL, USA). The protein fraction was mixed with loading buffer containing 10% 2-mercaptoethanol and heated at 95˚C for 10 min. Prepared samples were stored at -80˚C. Protein samples (30 µg) were loaded and electrophoresed on 12.5 % SDS polyacrylamide gels at 130 V for 1 h, and were transferred to a PVDF membrane at 110 V for 70 min. Next, membranes were blocked in 5% nonfat dry milk in TBST for 1 h and separate sets of membranes were probed with rabbit anti-SOD1 polyclonal antibody (1:500; Santa Cruz Biotechnology, USA) or goat anti-SOD2 polyclonal antibody (1:1000; Santa Cruz Biotechnology, USA) overnight at 4˚C. After two washes in TBST and washes in 1% milk in TBST the blots were incubated with peroxidase-conjugated anti-rabbit secondary antibody (1:7500; Santa Cruz Biotechnology, USA) or anti-goat secondary antibody (1:5000; Santa Cruz Biotechnology, USA) diluted in 1% milk in TBST in room temperature for 1 h. After washing the blots were detected with the ECL method using WesternBright Quantum chemiluminescent substrate (Advansta Inc., USA) and were imaged in G:Box (Syngene, USA). Next membranes were stripped using Restore Western Blot Stripping Buffer (Thermo Scientific, USA), blocked in 5% nonfat dry milk in TBST and re-probed with mouse monoclonal anti-ß-actin (1:1000; Santa Cruz Biotechnology, USA) using the same protocol as before. After washes, the blots were incubated in anti-mouse secondary antibody (1:5000; Santa Cruz Biotechnology, USA). The data were analyzed with Gene Tools (version 4.03 (a); Syngene, USA). The expression of SOD1 and SOD2 was evaluated relative to that of ß-actin. The Western blot and enzymes activity were performed in tissue from the same animal.45

## Slide 10
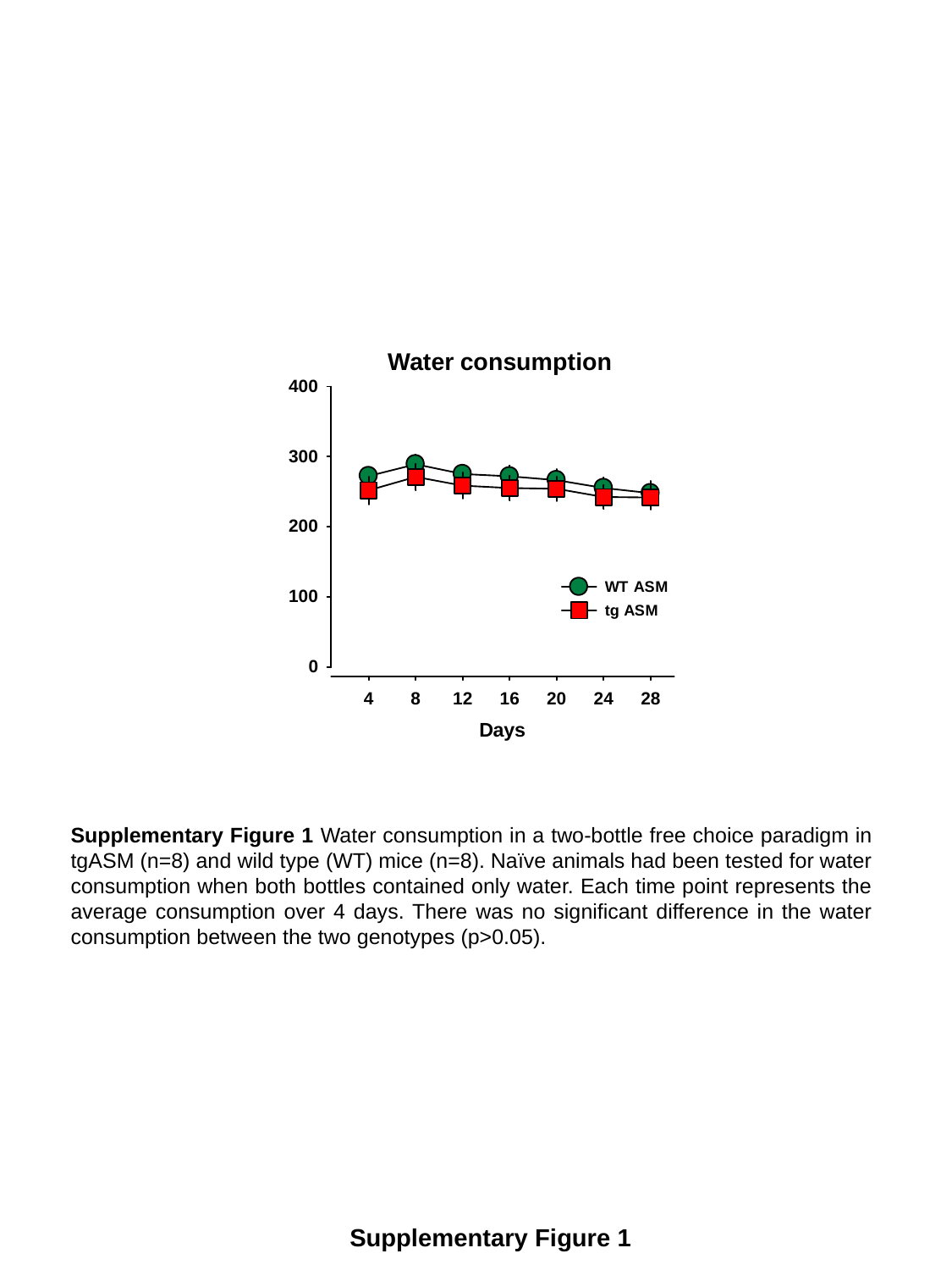

Supplementary Figure 1 Water consumption in a two-bottle free choice paradigm in tgASM (n=8) and wild type (WT) mice (n=8). Naïve animals had been tested for water consumption when both bottles contained only water. Each time point represents the average consumption over 4 days. There was no significant difference in the water consumption between the two genotypes (p>0.05).
Supplementary Figure 1

## Slide 11
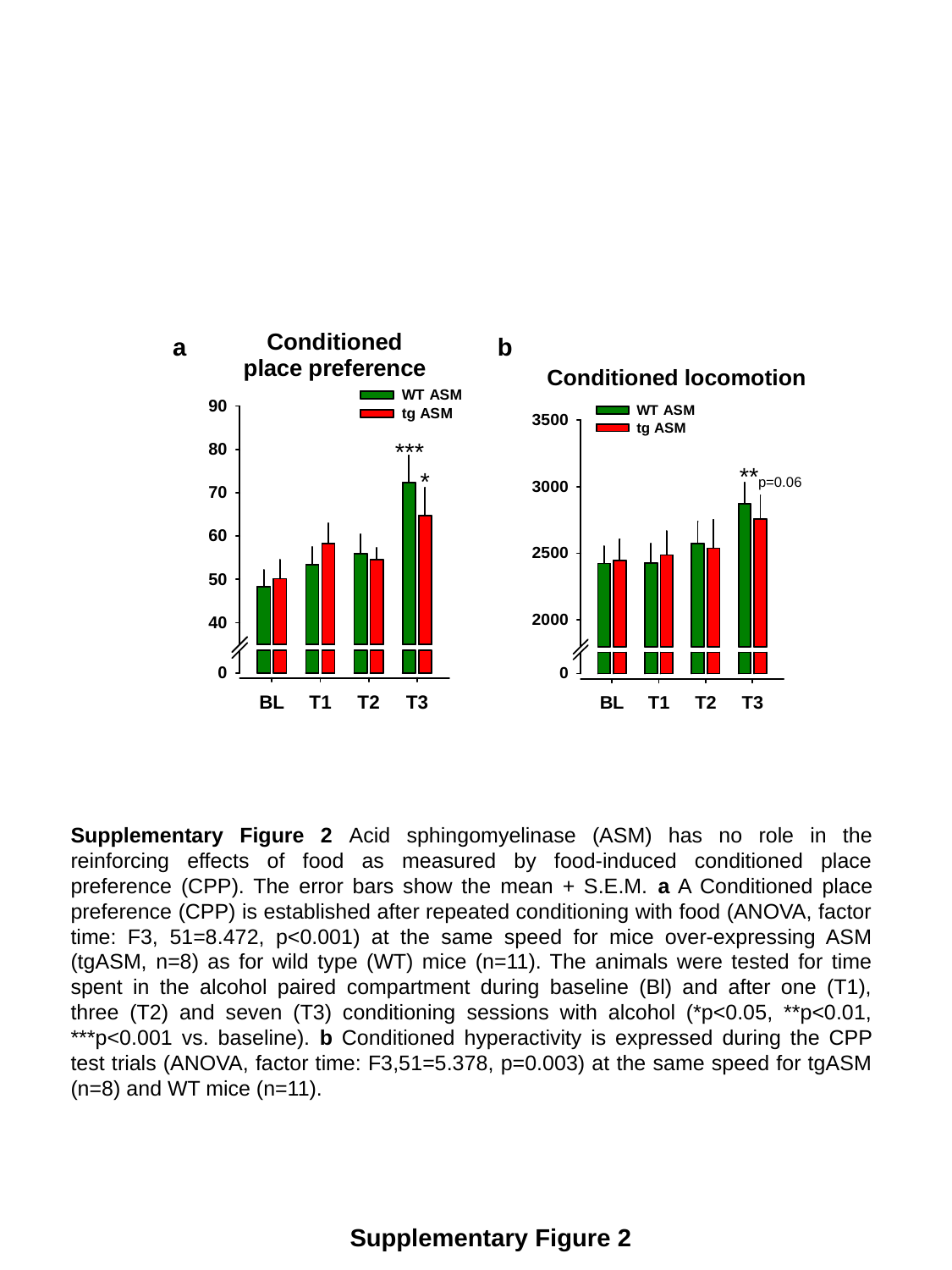

a
b
***
**
*
p=0.06
Supplementary Figure 2 Acid sphingomyelinase (ASM) has no role in the reinforcing effects of food as measured by food-induced conditioned place preference (CPP). The error bars show the mean + S.E.M. a A Conditioned place preference (CPP) is established after repeated conditioning with food (ANOVA, factor time: F3, 51=8.472, p<0.001) at the same speed for mice over-expressing ASM (tgASM, n=8) as for wild type (WT) mice (n=11). The animals were tested for time spent in the alcohol paired compartment during baseline (Bl) and after one (T1), three (T2) and seven (T3) conditioning sessions with alcohol (*p<0.05, **p<0.01, ***p<0.001 vs. baseline). b Conditioned hyperactivity is expressed during the CPP test trials (ANOVA, factor time: F3,51=5.378, p=0.003) at the same speed for tgASM (n=8) and WT mice (n=11).
Supplementary Figure 2

## Slide 12
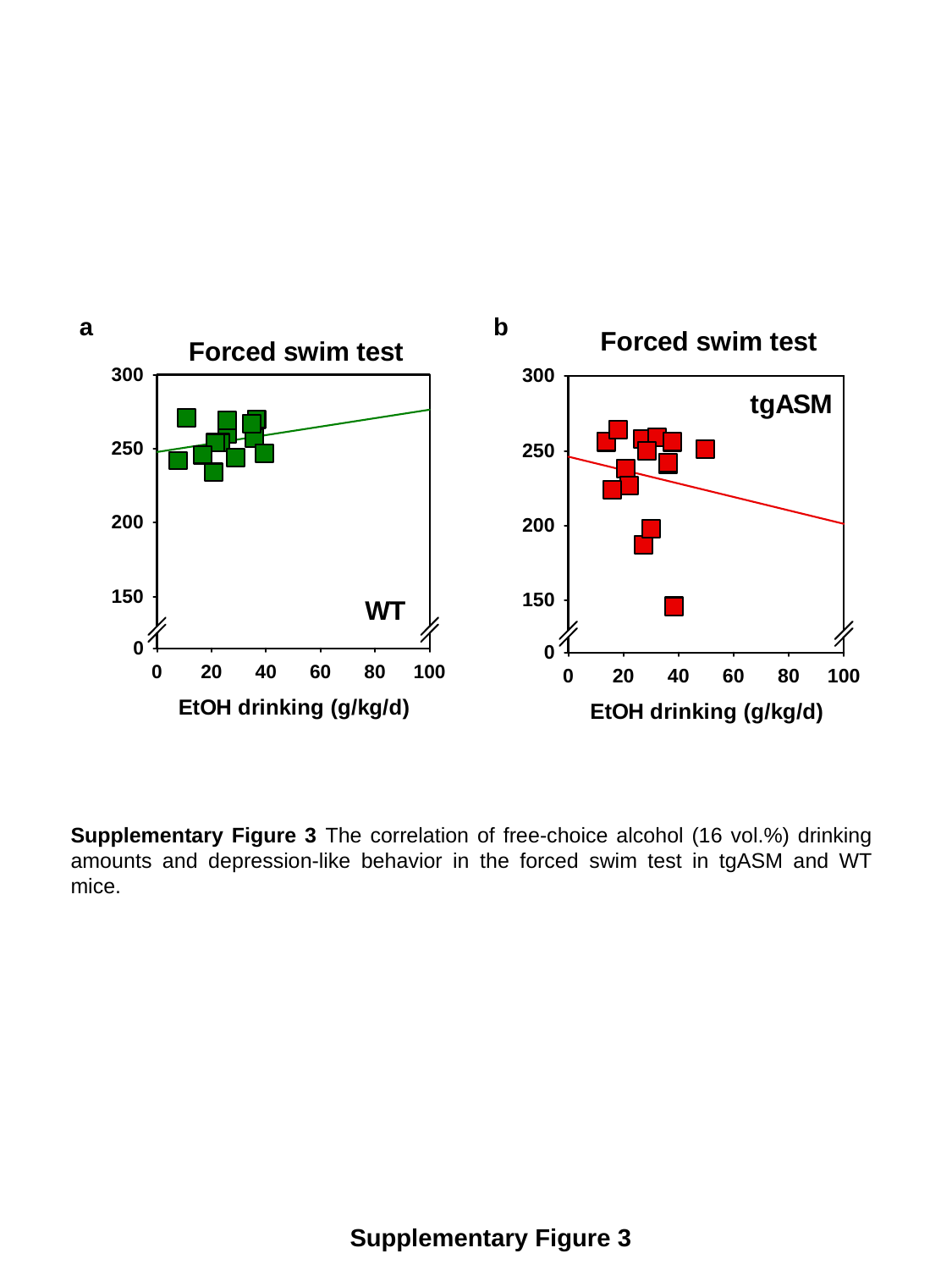

a
b
Supplementary Figure 3 The correlation of free-choice alcohol (16 vol.%) drinking amounts and depression-like behavior in the forced swim test in tgASM and WT mice.
Supplementary Figure 3

## Slide 13
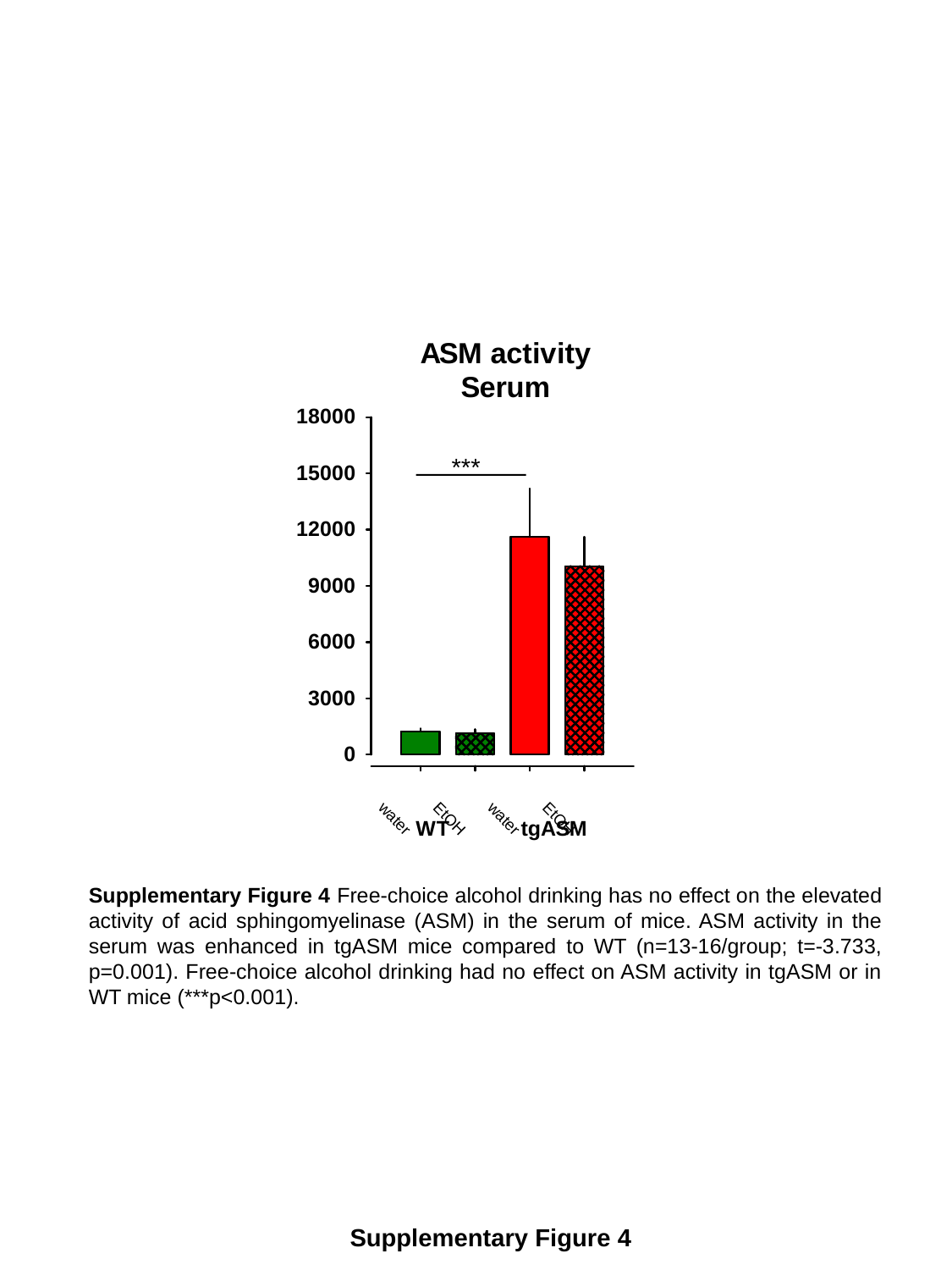

***
Supplementary Figure 4 Free-choice alcohol drinking has no effect on the elevated activity of acid sphingomyelinase (ASM) in the serum of mice. ASM activity in the serum was enhanced in tgASM mice compared to WT (n=13-16/group; t=-3.733, p=0.001). Free-choice alcohol drinking had no effect on ASM activity in tgASM or in WT mice (***p<0.001).
Supplementary Figure 4

## Slide 14
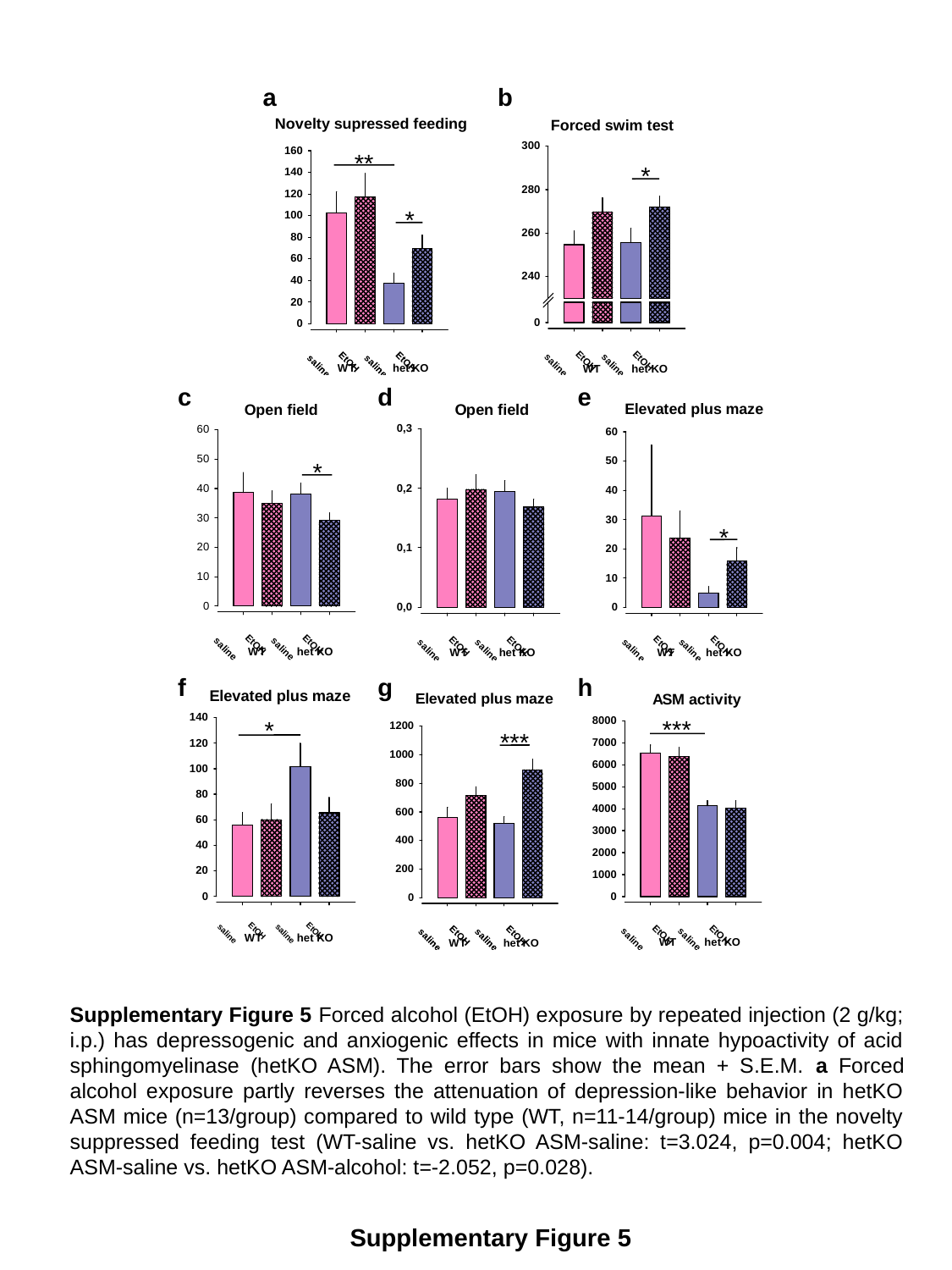

a
b
**
*
*
c
d
e
*
*
f
g
h
***
*
***
Supplementary Figure 5 Forced alcohol (EtOH) exposure by repeated injection (2 g/kg; i.p.) has depressogenic and anxiogenic effects in mice with innate hypoactivity of acid sphingomyelinase (hetKO ASM). The error bars show the mean + S.E.M. a Forced alcohol exposure partly reverses the attenuation of depression-like behavior in hetKO ASM mice (n=13/group) compared to wild type (WT, n=11-14/group) mice in the novelty suppressed feeding test (WT-saline vs. hetKO ASM-saline: t=3.024, p=0.004; hetKO ASM-saline vs. hetKO ASM-alcohol: t=-2.052, p=0.028).
Supplementary Figure 5

## Slide 15
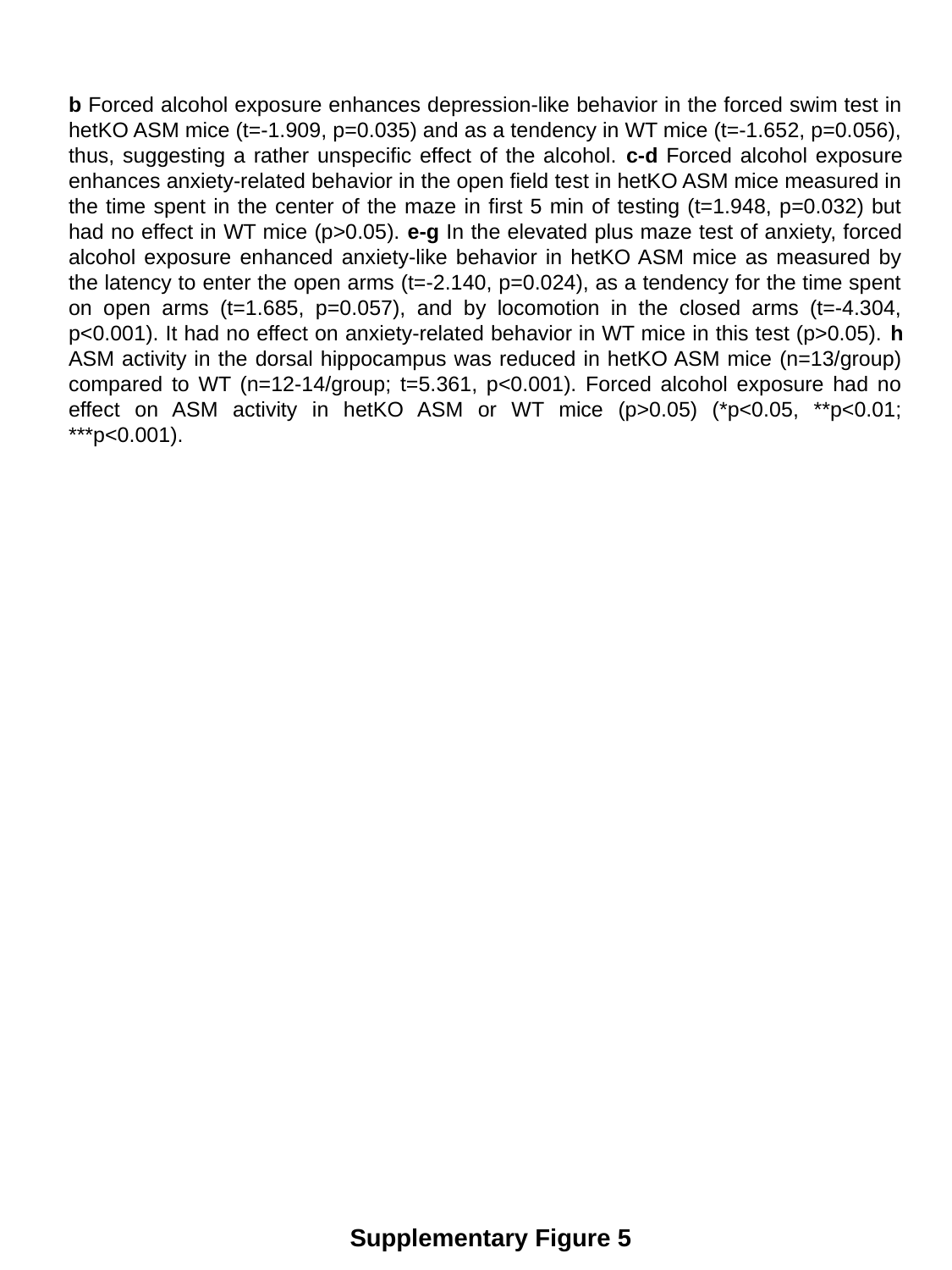

b Forced alcohol exposure enhances depression-like behavior in the forced swim test in hetKO ASM mice (t=-1.909, p=0.035) and as a tendency in WT mice (t=-1.652, p=0.056), thus, suggesting a rather unspecific effect of the alcohol. c-d Forced alcohol exposure enhances anxiety-related behavior in the open field test in hetKO ASM mice measured in the time spent in the center of the maze in first 5 min of testing (t=1.948, p=0.032) but had no effect in WT mice (p>0.05). e-g In the elevated plus maze test of anxiety, forced alcohol exposure enhanced anxiety-like behavior in hetKO ASM mice as measured by the latency to enter the open arms (t=-2.140, p=0.024), as a tendency for the time spent on open arms (t=1.685, p=0.057), and by locomotion in the closed arms (t=-4.304, p<0.001). It had no effect on anxiety-related behavior in WT mice in this test (p>0.05). h ASM activity in the dorsal hippocampus was reduced in hetKO ASM mice (n=13/group) compared to WT (n=12-14/group; t=5.361, p<0.001). Forced alcohol exposure had no effect on ASM activity in hetKO ASM or WT mice (p>0.05) (*p<0.05, **p<0.01; ***p<0.001).
Supplementary Figure 5

## Slide 16
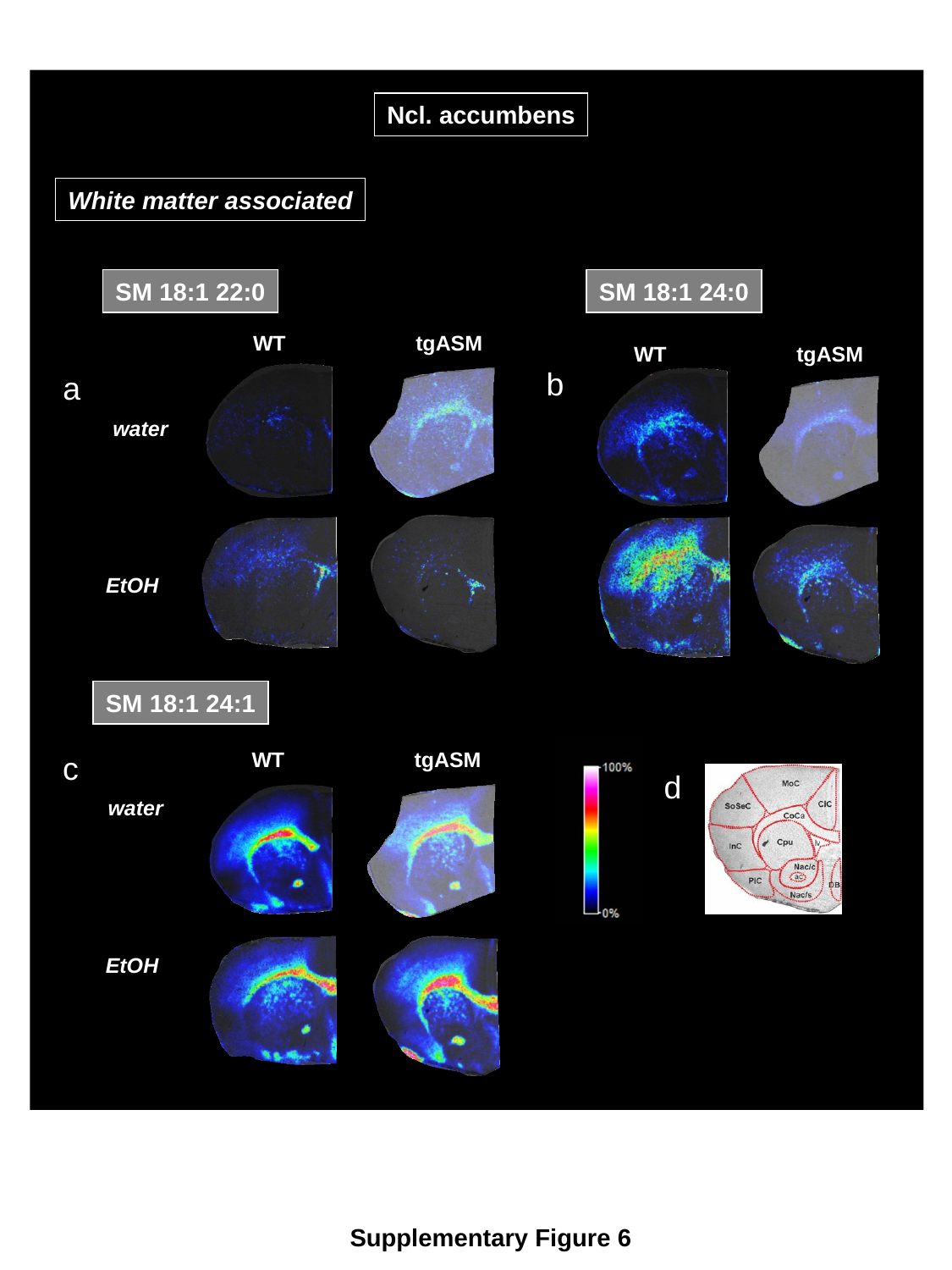

Ncl. accumbens
White matter associated
SM 18:1 22:0
WT tgASM
WT tgASM
a
water
EtOH
SM 18:1 24:1
c
water
EtOH
SM 18:1 24:0
WT tgASM
b
d
Supplementary Figure 6

## Slide 17
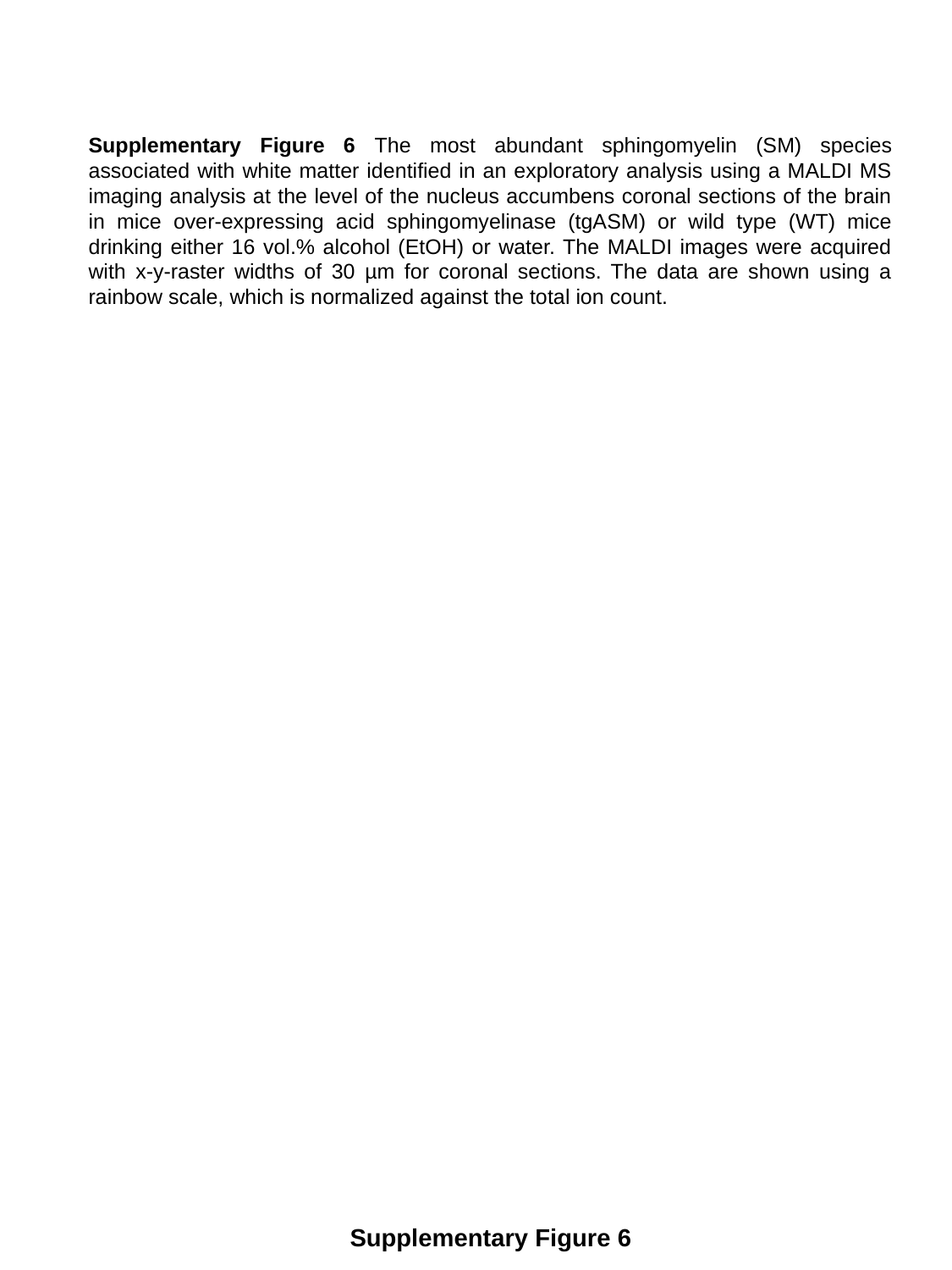

Supplementary Figure 6 The most abundant sphingomyelin (SM) species associated with white matter identified in an exploratory analysis using a MALDI MS imaging analysis at the level of the nucleus accumbens coronal sections of the brain in mice over-expressing acid sphingomyelinase (tgASM) or wild type (WT) mice drinking either 16 vol.% alcohol (EtOH) or water. The MALDI images were acquired with x-y-raster widths of 30 µm for coronal sections. The data are shown using a rainbow scale, which is normalized against the total ion count.
Supplementary Figure 6

## Slide 18
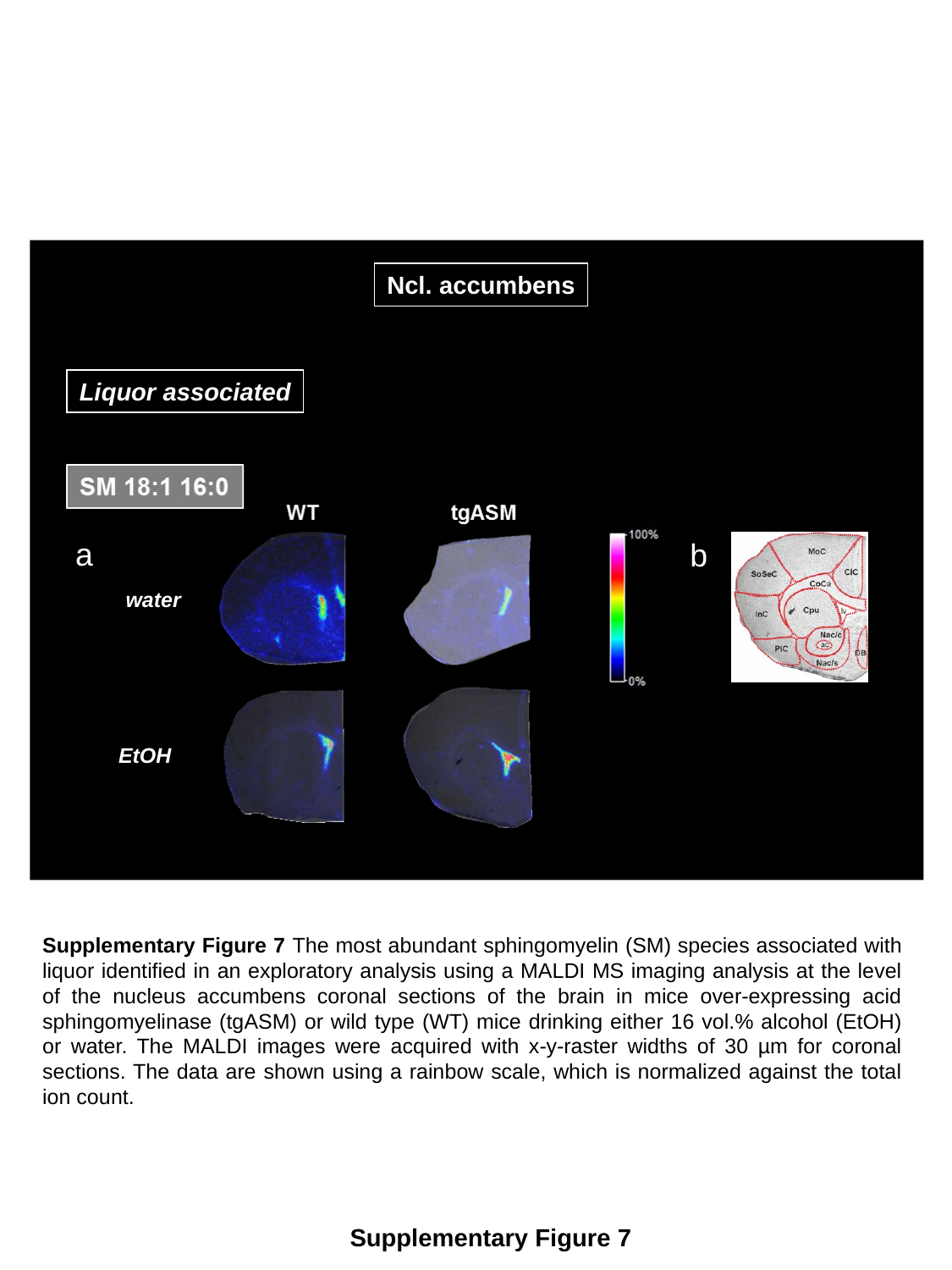

Ncl. accumbens
Liquor associated
a
b
water
EtOH
Supplementary Figure 7 The most abundant sphingomyelin (SM) species associated with liquor identified in an exploratory analysis using a MALDI MS imaging analysis at the level of the nucleus accumbens coronal sections of the brain in mice over-expressing acid sphingomyelinase (tgASM) or wild type (WT) mice drinking either 16 vol.% alcohol (EtOH) or water. The MALDI images were acquired with x-y-raster widths of 30 µm for coronal sections. The data are shown using a rainbow scale, which is normalized against the total ion count.
Supplementary Figure 7

## Slide 19
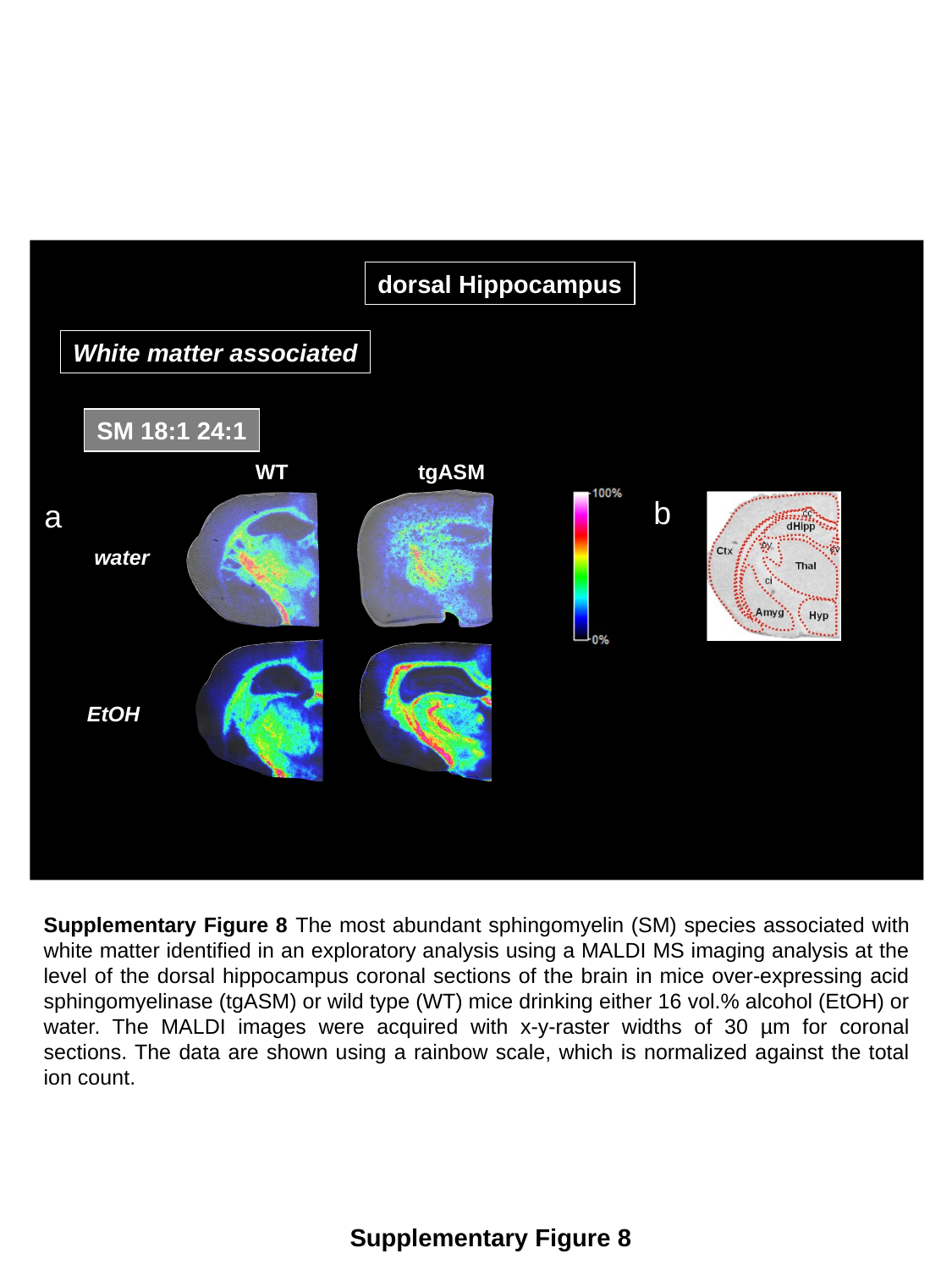

dorsal Hippocampus
White matter associated
SM 18:1 24:1
WT tgASM
b
a
water
EtOH
Supplementary Figure 8 The most abundant sphingomyelin (SM) species associated with white matter identified in an exploratory analysis using a MALDI MS imaging analysis at the level of the dorsal hippocampus coronal sections of the brain in mice over-expressing acid sphingomyelinase (tgASM) or wild type (WT) mice drinking either 16 vol.% alcohol (EtOH) or water. The MALDI images were acquired with x-y-raster widths of 30 µm for coronal sections. The data are shown using a rainbow scale, which is normalized against the total ion count.
Supplementary Figure 8

## Slide 20
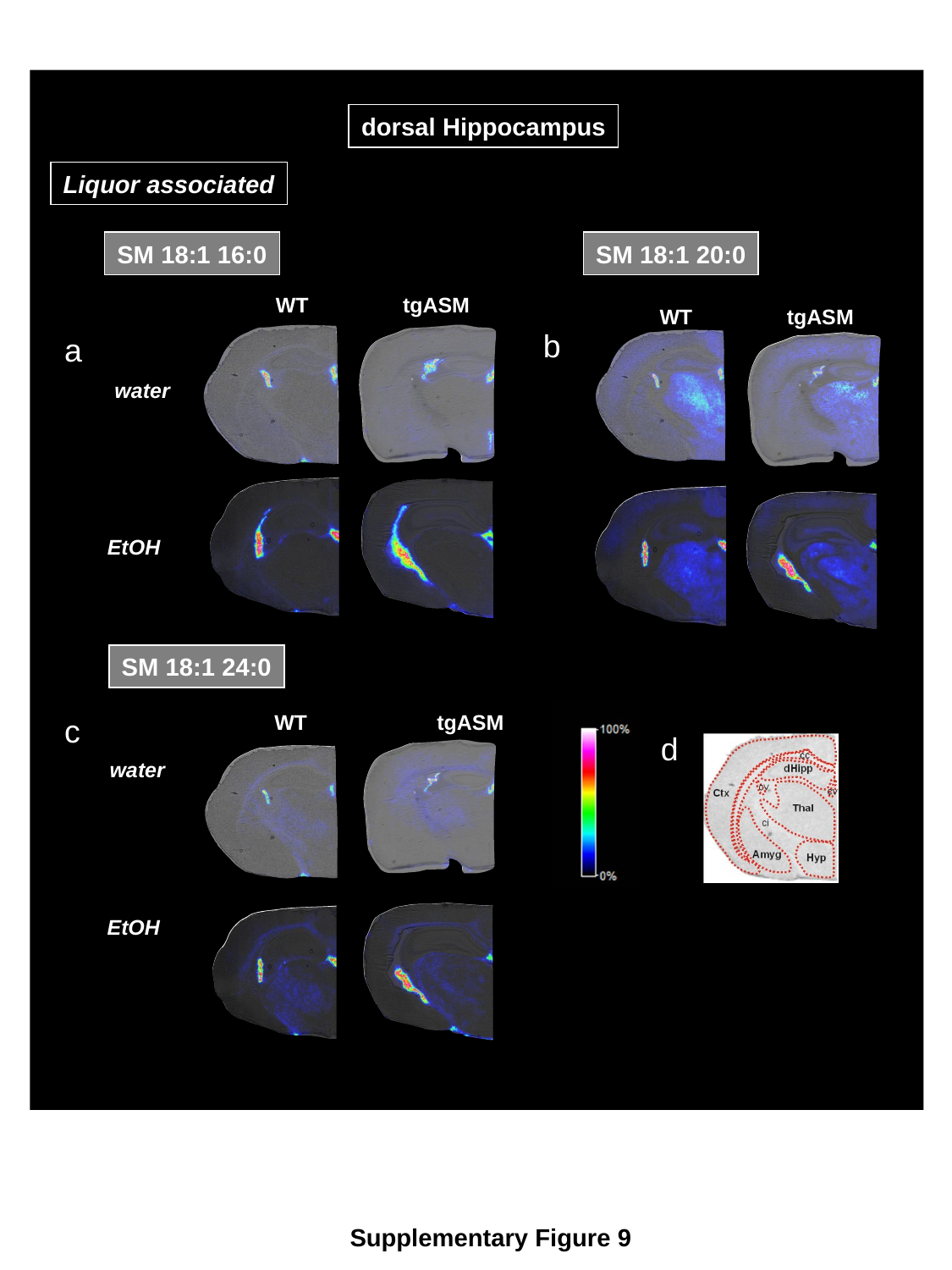

dorsal Hippocampus
Liquor associated
SM 18:1 16:0
WT tgASM
a
water
EtOH
SM 18:1 24:0
WT tgASM
c
water
EtOH
SM 18:1 20:0
WT tgASM
b
d
Supplementary Figure 9

## Slide 21
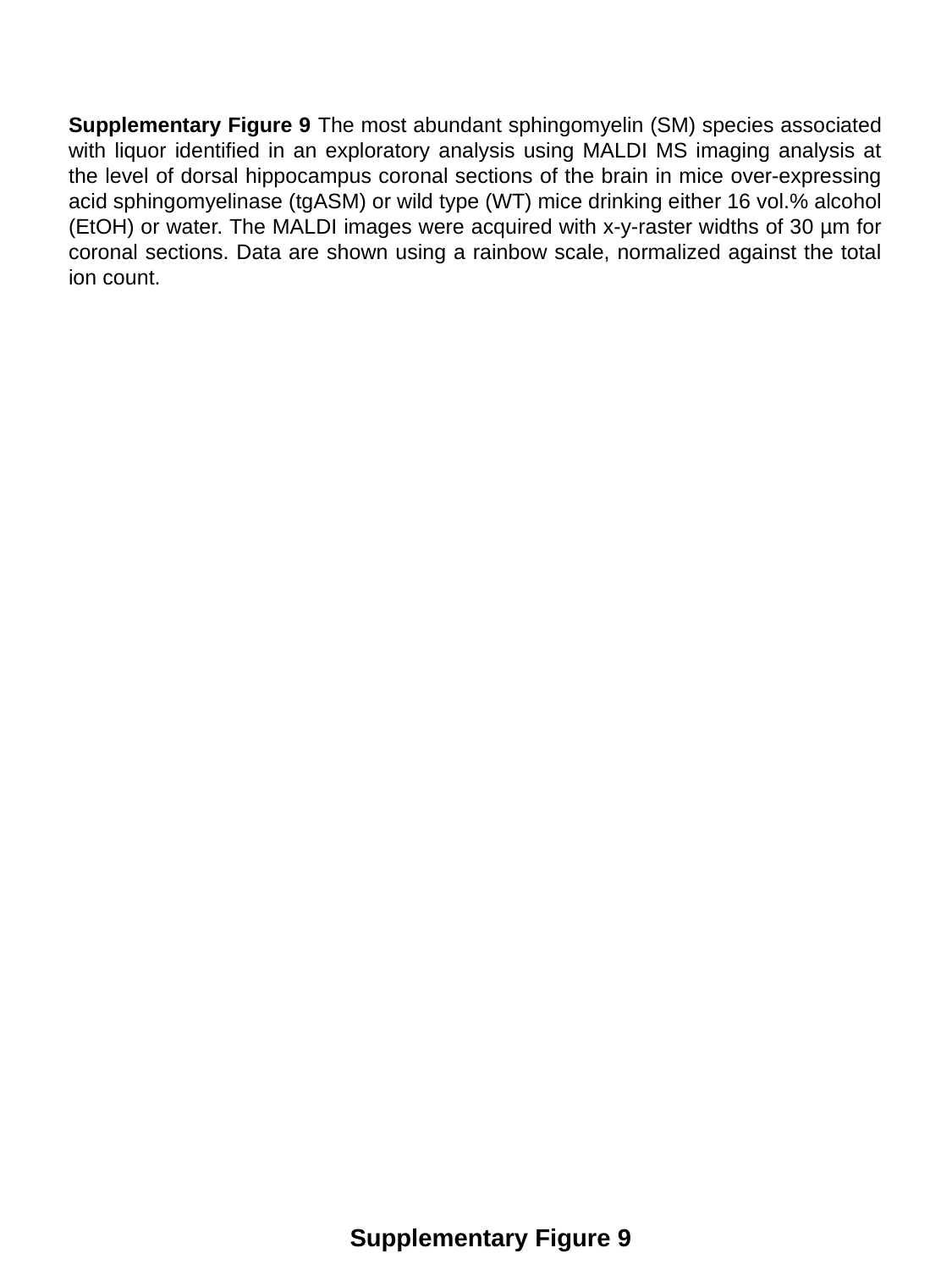

Supplementary Figure 9 The most abundant sphingomyelin (SM) species associated with liquor identified in an exploratory analysis using MALDI MS imaging analysis at the level of dorsal hippocampus coronal sections of the brain in mice over-expressing acid sphingomyelinase (tgASM) or wild type (WT) mice drinking either 16 vol.% alcohol (EtOH) or water. The MALDI images were acquired with x-y-raster widths of 30 µm for coronal sections. Data are shown using a rainbow scale, normalized against the total ion count.
Supplementary Figure 9

## Slide 22
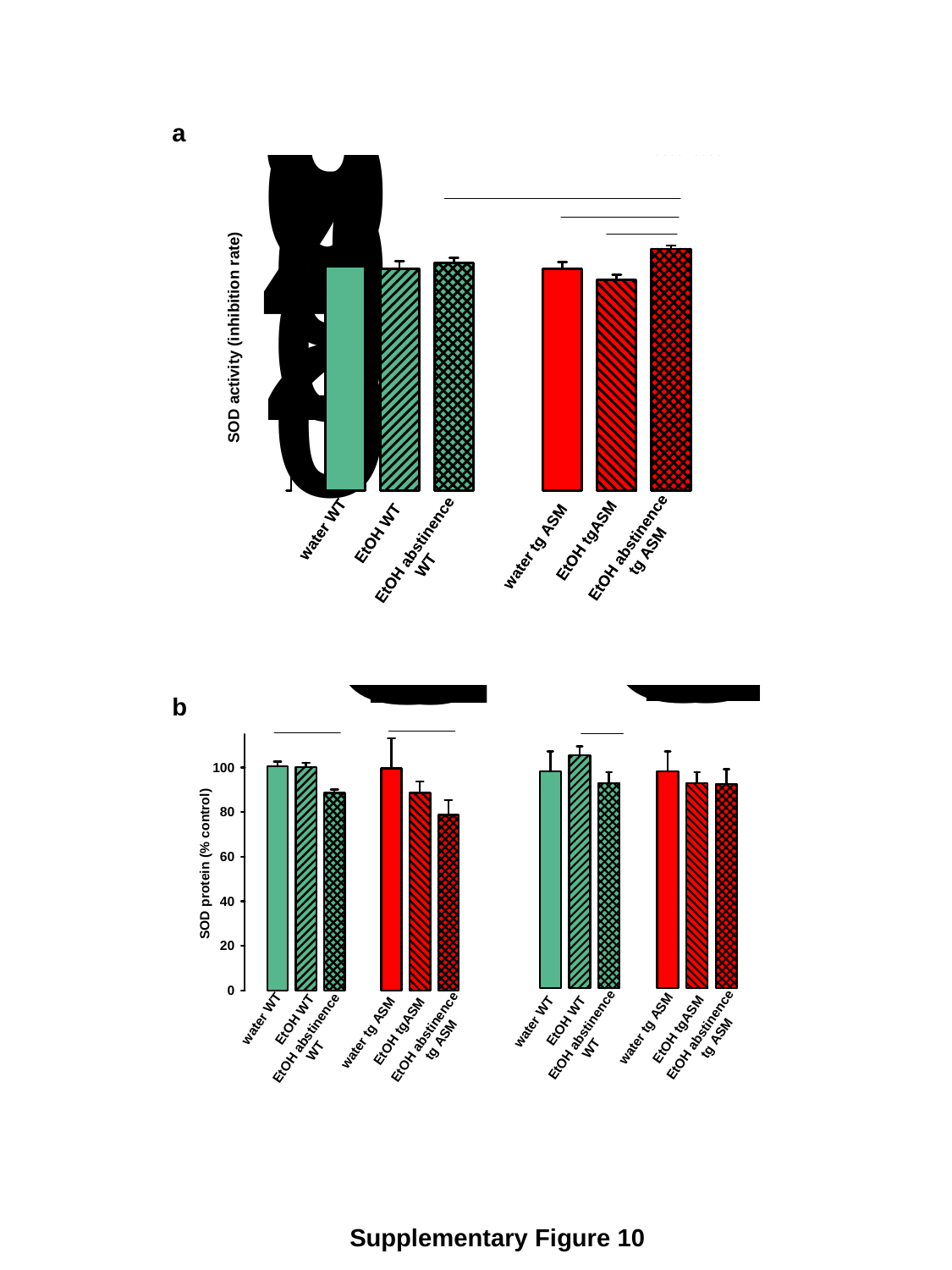

a
b
Supplementary Figure 10

## Slide 23
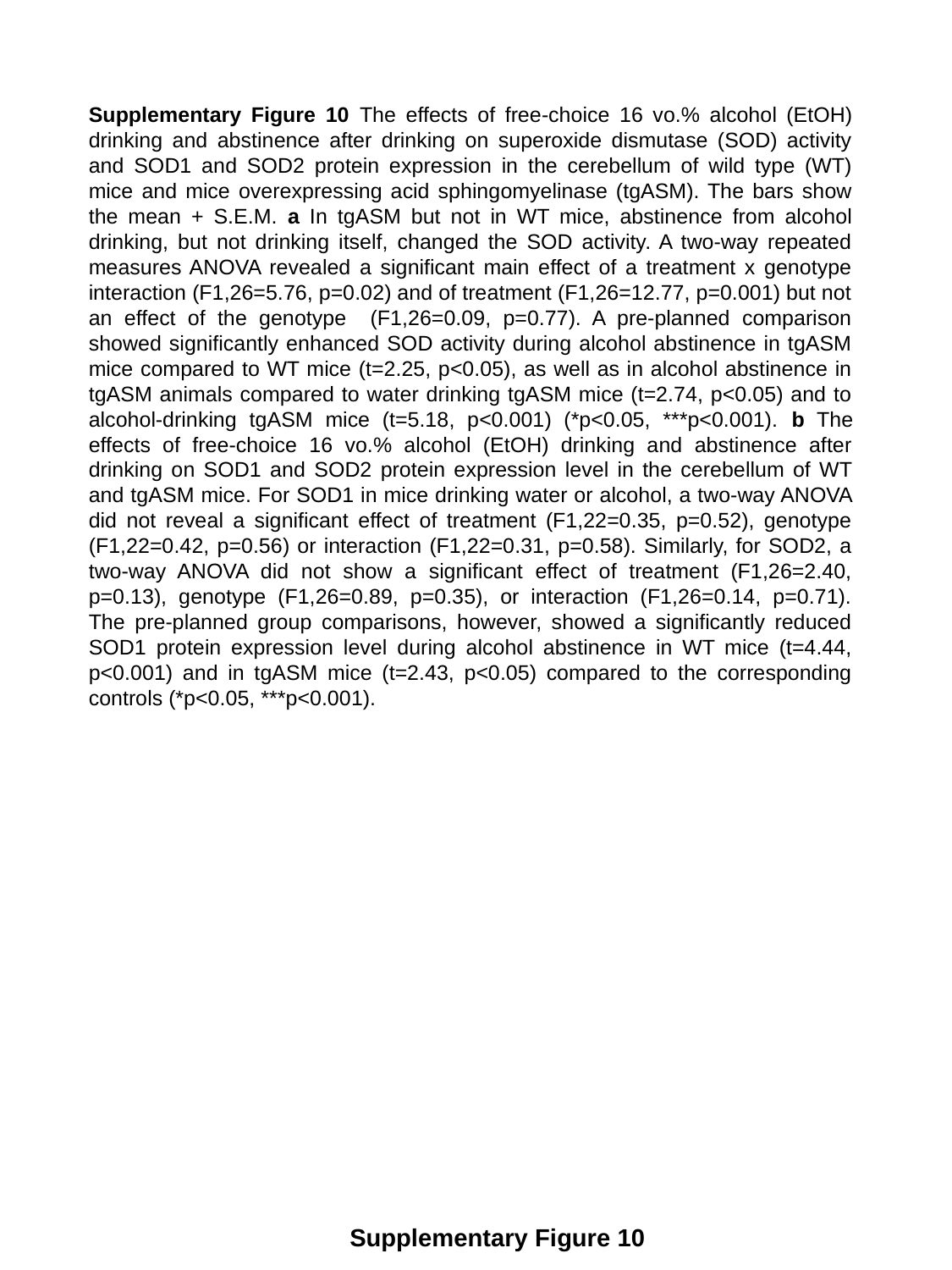

Supplementary Figure 10 The effects of free-choice 16 vo.% alcohol (EtOH) drinking and abstinence after drinking on superoxide dismutase (SOD) activity and SOD1 and SOD2 protein expression in the cerebellum of wild type (WT) mice and mice overexpressing acid sphingomyelinase (tgASM). The bars show the mean + S.E.M. a In tgASM but not in WT mice, abstinence from alcohol drinking, but not drinking itself, changed the SOD activity. A two-way repeated measures ANOVA revealed a significant main effect of a treatment x genotype interaction (F1,26=5.76, p=0.02) and of treatment (F1,26=12.77, p=0.001) but not an effect of the genotype (F1,26=0.09, p=0.77). A pre-planned comparison showed significantly enhanced SOD activity during alcohol abstinence in tgASM mice compared to WT mice (t=2.25, p<0.05), as well as in alcohol abstinence in tgASM animals compared to water drinking tgASM mice (t=2.74, p<0.05) and to alcohol-drinking tgASM mice (t=5.18, p<0.001) (*p<0.05, ***p<0.001). b The effects of free-choice 16 vo.% alcohol (EtOH) drinking and abstinence after drinking on SOD1 and SOD2 protein expression level in the cerebellum of WT and tgASM mice. For SOD1 in mice drinking water or alcohol, a two-way ANOVA did not reveal a significant effect of treatment (F1,22=0.35, p=0.52), genotype (F1,22=0.42, p=0.56) or interaction (F1,22=0.31, p=0.58). Similarly, for SOD2, a two-way ANOVA did not show a significant effect of treatment (F1,26=2.40, p=0.13), genotype (F1,26=0.89, p=0.35), or interaction (F1,26=0.14, p=0.71). The pre-planned group comparisons, however, showed a significantly reduced SOD1 protein expression level during alcohol abstinence in WT mice (t=4.44, p<0.001) and in tgASM mice (t=2.43, p<0.05) compared to the corresponding controls (*p<0.05, ***p<0.001).
Supplementary Figure 10

## Slide 24
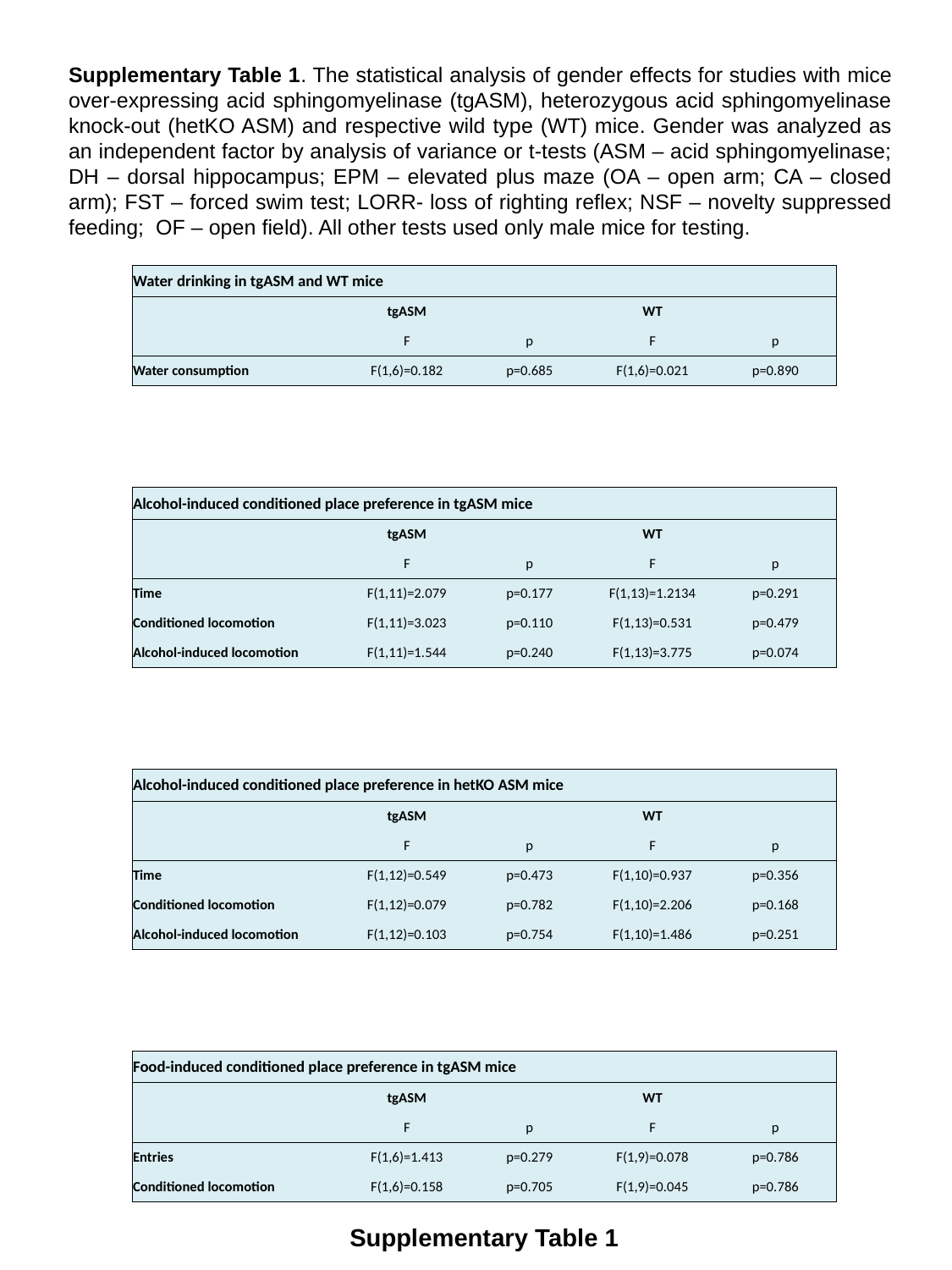

Supplementary Table 1. The statistical analysis of gender effects for studies with mice over-expressing acid sphingomyelinase (tgASM), heterozygous acid sphingomyelinase knock-out (hetKO ASM) and respective wild type (WT) mice. Gender was analyzed as an independent factor by analysis of variance or t-tests (ASM – acid sphingomyelinase; DH – dorsal hippocampus; EPM – elevated plus maze (OA – open arm; CA – closed arm); FST – forced swim test; LORR- loss of righting reflex; NSF – novelty suppressed feeding; OF – open field). All other tests used only male mice for testing.
| Water drinking in tgASM and WT mice | | | | |
| --- | --- | --- | --- | --- |
| | tgASM | | WT | |
| | F | p | F | p |
| Water consumption | F(1,6)=0.182 | p=0.685 | F(1,6)=0.021 | p=0.890 |
| | | | | |
| | | | | |
| Alcohol-induced conditioned place preference in tgASM mice | | | | |
| | tgASM | | WT | |
| | F | p | F | p |
| Time | F(1,11)=2.079 | p=0.177 | F(1,13)=1.2134 | p=0.291 |
| Conditioned locomotion | F(1,11)=3.023 | p=0.110 | F(1,13)=0.531 | p=0.479 |
| Alcohol-induced locomotion | F(1,11)=1.544 | p=0.240 | F(1,13)=3.775 | p=0.074 |
| | | | | |
| | | | | |
| Alcohol-induced conditioned place preference in hetKO ASM mice | | | | |
| | tgASM | | WT | |
| | F | p | F | p |
| Time | F(1,12)=0.549 | p=0.473 | F(1,10)=0.937 | p=0.356 |
| Conditioned locomotion | F(1,12)=0.079 | p=0.782 | F(1,10)=2.206 | p=0.168 |
| Alcohol-induced locomotion | F(1,12)=0.103 | p=0.754 | F(1,10)=1.486 | p=0.251 |
| | | | | |
| | | | | |
| Food-induced conditioned place preference in tgASM mice | | | | |
| | tgASM | | WT | |
| | F | p | F | p |
| Entries | F(1,6)=1.413 | p=0.279 | F(1,9)=0.078 | p=0.786 |
| Conditioned locomotion | F(1,6)=0.158 | p=0.705 | F(1,9)=0.045 | p=0.786 |
| | | | | |
| | | | | |
| | | | | |
| LORR in tgASM mice | | | | |
| | tgASM | | WT | |
| | t | p | t | p |
| Latency | -0.054 | 0.959 | 0.357 | 0.739 |
| Duration | 1.664 | 0.171 | 1.455 | 0.219 |
| | | | | |
| | | | | |
| | | | | |
| Blood alcohol concentration (BAC) after alcohol (i.p.) injection in tgASM and hetKO ASM mice | | | | |
| | F | p | F | p |
| tgASM | F(1,5)=3.842 | 0.107 | F(1,6)=2.662 | 0.154 |
| hetKO ASM | F(1,7)=0.853 | 0.386 | F(1,4)=0.149 | 0.719 |
Supplementary Table 1

## Slide 25
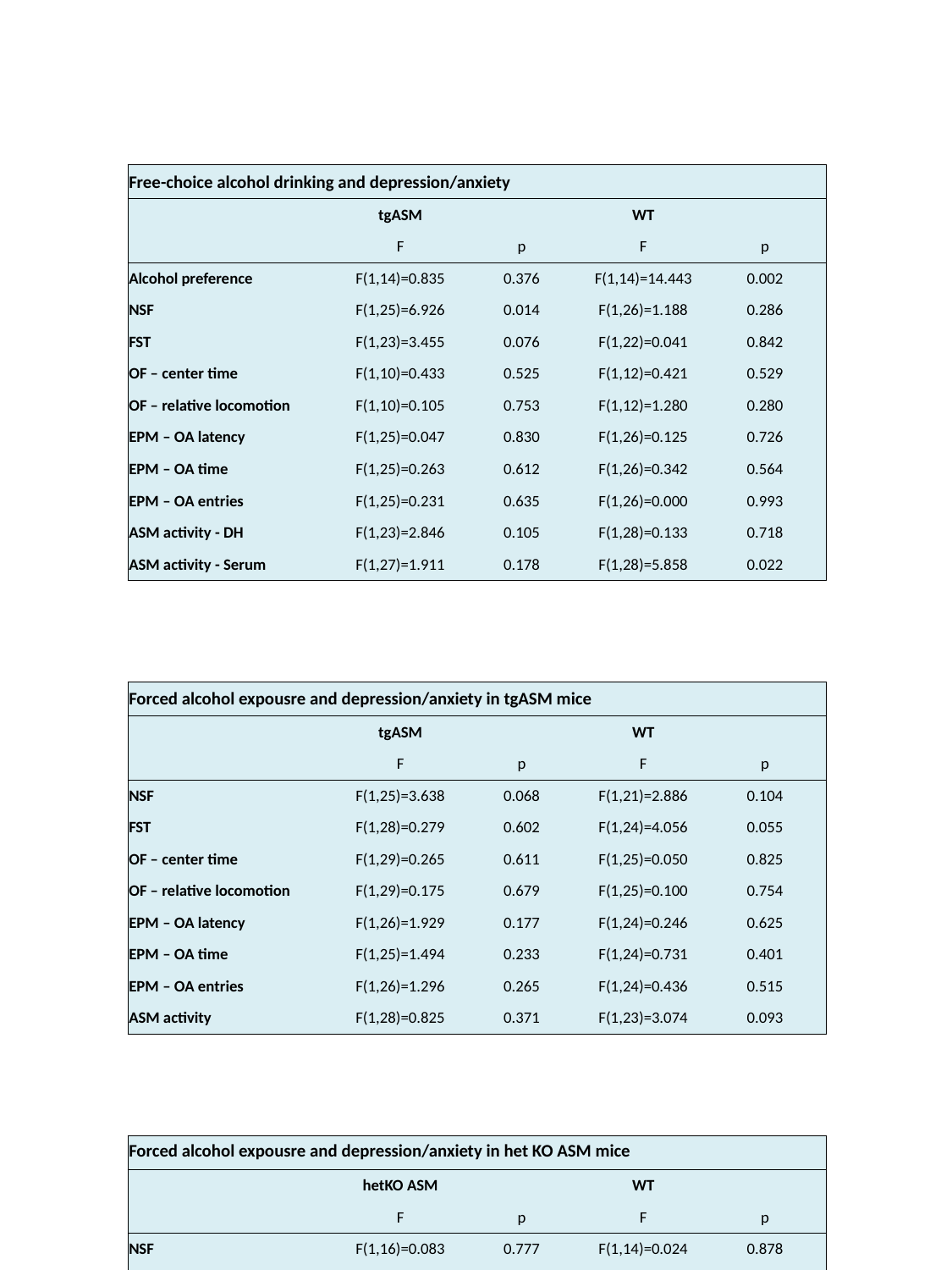

| Free-choice alcohol drinking and depression/anxiety | | | | |
| --- | --- | --- | --- | --- |
| | tgASM | | WT | |
| | F | p | F | p |
| Alcohol preference | F(1,14)=0.835 | 0.376 | F(1,14)=14.443 | 0.002 |
| NSF | F(1,25)=6.926 | 0.014 | F(1,26)=1.188 | 0.286 |
| FST | F(1,23)=3.455 | 0.076 | F(1,22)=0.041 | 0.842 |
| OF – center time | F(1,10)=0.433 | 0.525 | F(1,12)=0.421 | 0.529 |
| OF – relative locomotion | F(1,10)=0.105 | 0.753 | F(1,12)=1.280 | 0.280 |
| EPM – OA latency | F(1,25)=0.047 | 0.830 | F(1,26)=0.125 | 0.726 |
| EPM – OA time | F(1,25)=0.263 | 0.612 | F(1,26)=0.342 | 0.564 |
| EPM – OA entries | F(1,25)=0.231 | 0.635 | F(1,26)=0.000 | 0.993 |
| ASM activity - DH | F(1,23)=2.846 | 0.105 | F(1,28)=0.133 | 0.718 |
| ASM activity - Serum | F(1,27)=1.911 | 0.178 | F(1,28)=5.858 | 0.022 |
| | | | | |
| | | | | |
| Forced alcohol expousre and depression/anxiety in tgASM mice | | | | |
| | tgASM | | WT | |
| | F | p | F | p |
| NSF | F(1,25)=3.638 | 0.068 | F(1,21)=2.886 | 0.104 |
| FST | F(1,28)=0.279 | 0.602 | F(1,24)=4.056 | 0.055 |
| OF – center time | F(1,29)=0.265 | 0.611 | F(1,25)=0.050 | 0.825 |
| OF – relative locomotion | F(1,29)=0.175 | 0.679 | F(1,25)=0.100 | 0.754 |
| EPM – OA latency | F(1,26)=1.929 | 0.177 | F(1,24)=0.246 | 0.625 |
| EPM – OA time | F(1,25)=1.494 | 0.233 | F(1,24)=0.731 | 0.401 |
| EPM – OA entries | F(1,26)=1.296 | 0.265 | F(1,24)=0.436 | 0.515 |
| ASM activity | F(1,28)=0.825 | 0.371 | F(1,23)=3.074 | 0.093 |
| | | | | |
| | | | | |
| Forced alcohol expousre and depression/anxiety in het KO ASM mice | | | | |
| | hetKO ASM | | WT | |
| | F | p | F | p |
| NSF | F(1,16)=0.083 | 0.777 | F(1,14)=0.024 | 0.878 |
| FST | F(1,22)=0.949 | 0.340 | F(1,15)=0.010 | 0.923 |
| OF – center time | F(1,22)=0.161 | 0.692 | F(1,15)=0.200 | 0.661 |
| OF – relative locomotion | F(1,22)=0.498 | 0.488 | F(1,15)=0.444 | 0.515 |
| EPM – OA latency | F(1,20)=0.926 | 0.347 | F(1,13)=1.351 | 0.266 |
| EPM – OA time | F(1,16)=0.789 | 0.387 | F(1,13)=0.946 | 0.341 |
| EPM – CA locomotion | F(1,20)=2.945 | 0.102 | F(1,13)=19.756 | 0.001 |
| ASM activity | F(1,22)=2.961 | 0.099 | F(1,16)=1.308 | 0.270 |
Supplementary Table 1

## Slide 26
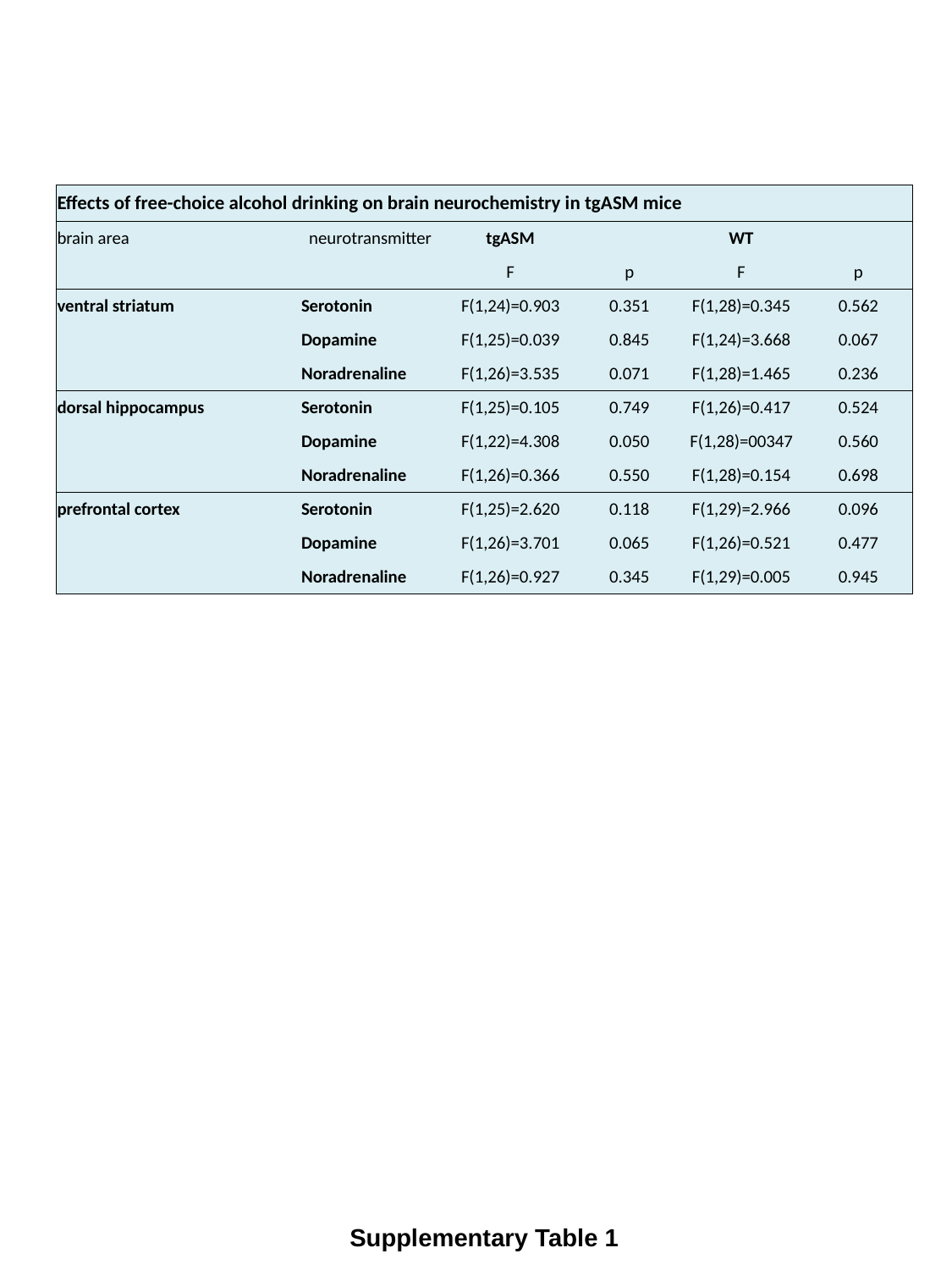

| Effects of free-choice alcohol drinking on brain neurochemistry in tgASM mice | | | | | |
| --- | --- | --- | --- | --- | --- |
| brain area | neurotransmitter | tgASM | | WT | |
| | | F | p | F | p |
| ventral striatum | Serotonin | F(1,24)=0.903 | 0.351 | F(1,28)=0.345 | 0.562 |
| | Dopamine | F(1,25)=0.039 | 0.845 | F(1,24)=3.668 | 0.067 |
| | Noradrenaline | F(1,26)=3.535 | 0.071 | F(1,28)=1.465 | 0.236 |
| dorsal hippocampus | Serotonin | F(1,25)=0.105 | 0.749 | F(1,26)=0.417 | 0.524 |
| | Dopamine | F(1,22)=4.308 | 0.050 | F(1,28)=00347 | 0.560 |
| | Noradrenaline | F(1,26)=0.366 | 0.550 | F(1,28)=0.154 | 0.698 |
| prefrontal cortex | Serotonin | F(1,25)=2.620 | 0.118 | F(1,29)=2.966 | 0.096 |
| | Dopamine | F(1,26)=3.701 | 0.065 | F(1,26)=0.521 | 0.477 |
| | Noradrenaline | F(1,26)=0.927 | 0.345 | F(1,29)=0.005 | 0.945 |
Supplementary Table 1

## Slide 27
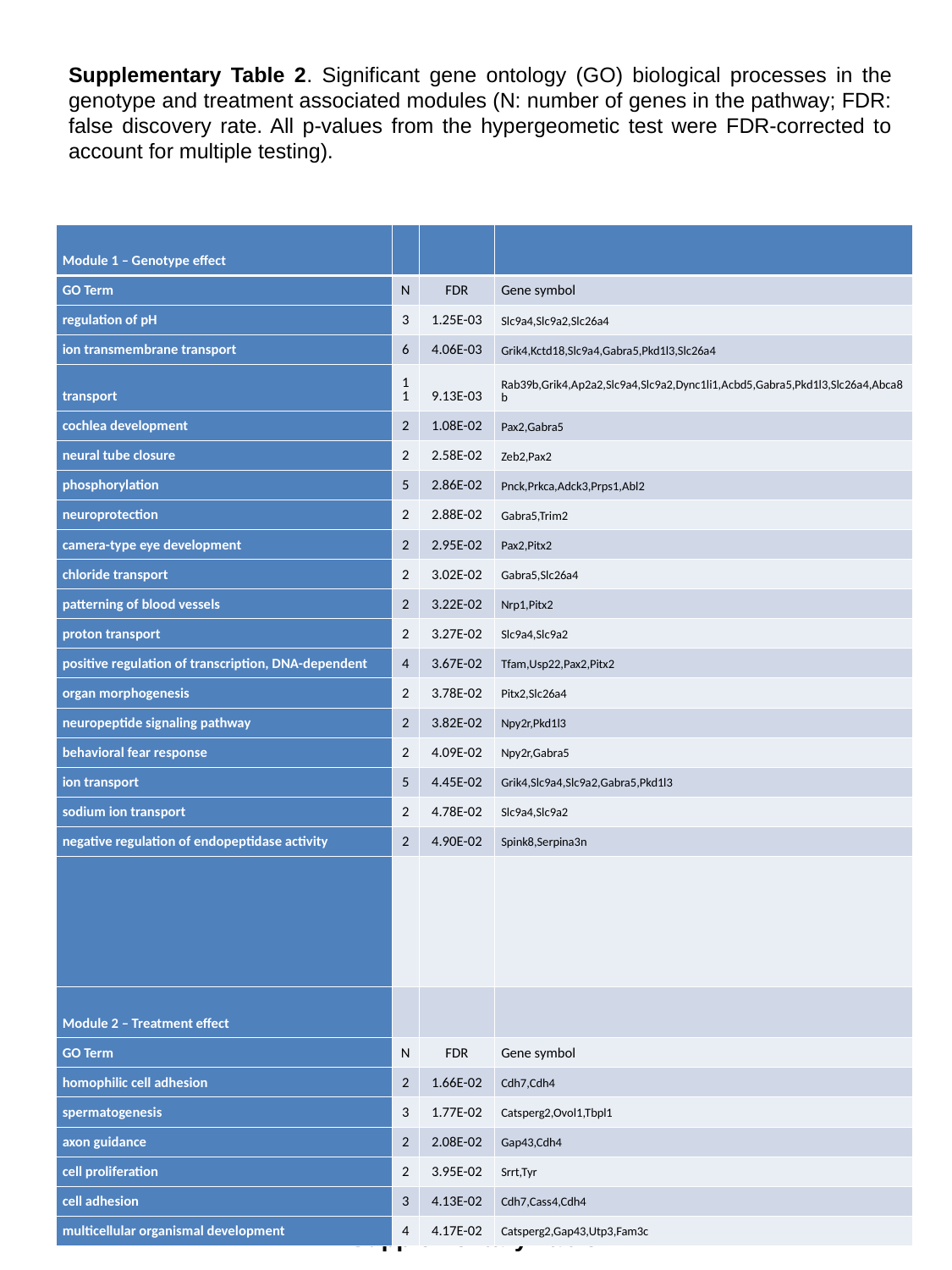

Supplementary Table 2. Significant gene ontology (GO) biological processes in the genotype and treatment associated modules (N: number of genes in the pathway; FDR: false discovery rate. All p-values from the hypergeometic test were FDR-corrected to account for multiple testing).
| Module 1 – Genotype effect | | | |
| --- | --- | --- | --- |
| GO Term | N | FDR | Gene symbol |
| regulation of pH | 3 | 1.25E-03 | Slc9a4,Slc9a2,Slc26a4 |
| ion transmembrane transport | 6 | 4.06E-03 | Grik4,Kctd18,Slc9a4,Gabra5,Pkd1l3,Slc26a4 |
| transport | 11 | 9.13E-03 | Rab39b,Grik4,Ap2a2,Slc9a4,Slc9a2,Dync1li1,Acbd5,Gabra5,Pkd1l3,Slc26a4,Abca8b |
| cochlea development | 2 | 1.08E-02 | Pax2,Gabra5 |
| neural tube closure | 2 | 2.58E-02 | Zeb2,Pax2 |
| phosphorylation | 5 | 2.86E-02 | Pnck,Prkca,Adck3,Prps1,Abl2 |
| neuroprotection | 2 | 2.88E-02 | Gabra5,Trim2 |
| camera-type eye development | 2 | 2.95E-02 | Pax2,Pitx2 |
| chloride transport | 2 | 3.02E-02 | Gabra5,Slc26a4 |
| patterning of blood vessels | 2 | 3.22E-02 | Nrp1,Pitx2 |
| proton transport | 2 | 3.27E-02 | Slc9a4,Slc9a2 |
| positive regulation of transcription, DNA-dependent | 4 | 3.67E-02 | Tfam,Usp22,Pax2,Pitx2 |
| organ morphogenesis | 2 | 3.78E-02 | Pitx2,Slc26a4 |
| neuropeptide signaling pathway | 2 | 3.82E-02 | Npy2r,Pkd1l3 |
| behavioral fear response | 2 | 4.09E-02 | Npy2r,Gabra5 |
| ion transport | 5 | 4.45E-02 | Grik4,Slc9a4,Slc9a2,Gabra5,Pkd1l3 |
| sodium ion transport | 2 | 4.78E-02 | Slc9a4,Slc9a2 |
| negative regulation of endopeptidase activity | 2 | 4.90E-02 | Spink8,Serpina3n |
| | | | |
| Module 2 – Treatment effect | | | |
| GO Term | N | FDR | Gene symbol |
| homophilic cell adhesion | 2 | 1.66E-02 | Cdh7,Cdh4 |
| spermatogenesis | 3 | 1.77E-02 | Catsperg2,Ovol1,Tbpl1 |
| axon guidance | 2 | 2.08E-02 | Gap43,Cdh4 |
| cell proliferation | 2 | 3.95E-02 | Srrt,Tyr |
| cell adhesion | 3 | 4.13E-02 | Cdh7,Cass4,Cdh4 |
| multicellular organismal development | 4 | 4.17E-02 | Catsperg2,Gap43,Utp3,Fam3c |
Supplementary Table 2

## Slide 28
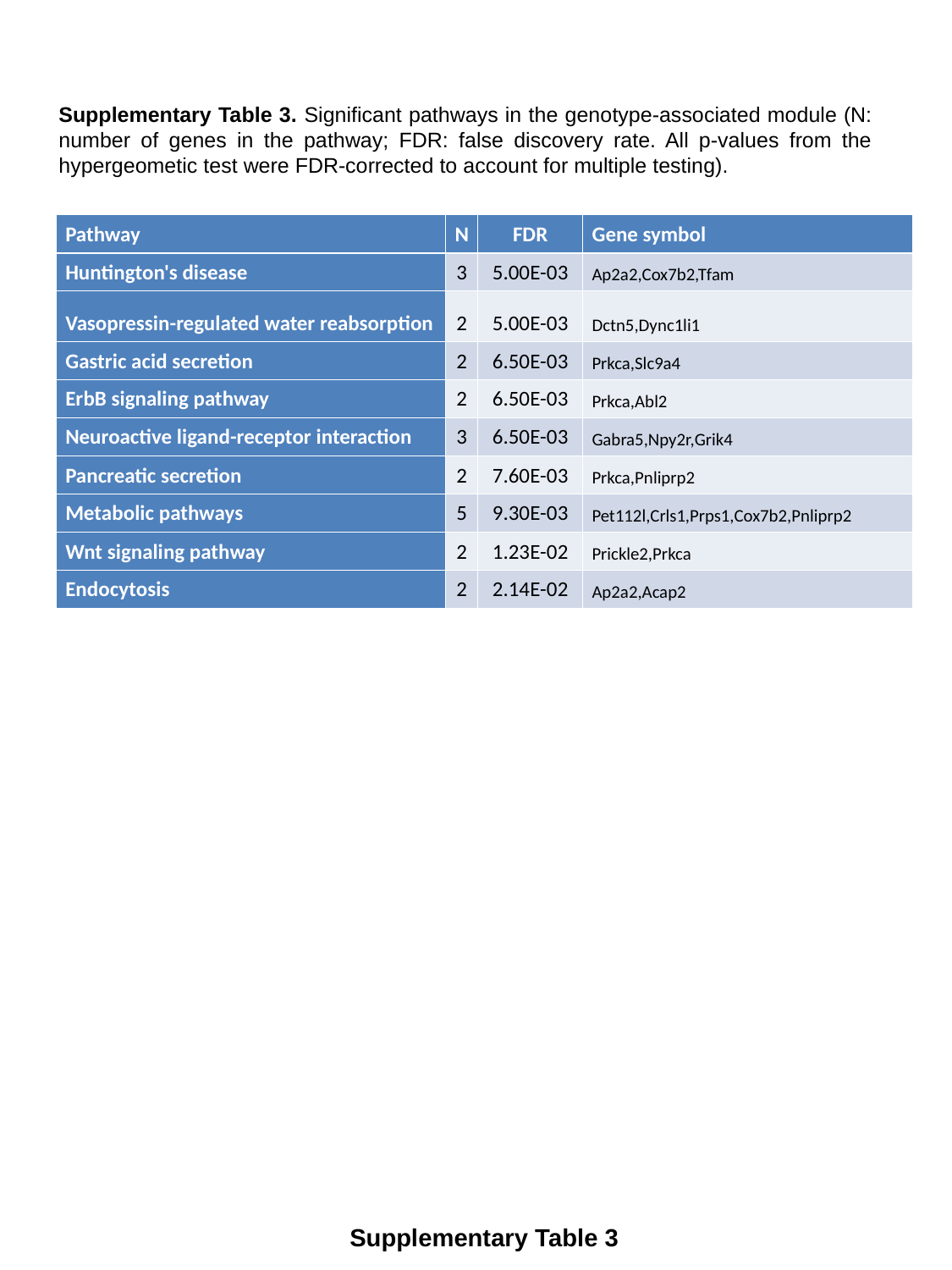

Supplementary Table 3. Significant pathways in the genotype-associated module (N: number of genes in the pathway; FDR: false discovery rate. All p-values from the hypergeometic test were FDR-corrected to account for multiple testing).
| Pathway | N | FDR | Gene symbol |
| --- | --- | --- | --- |
| Huntington's disease | 3 | 5.00E-03 | Ap2a2,Cox7b2,Tfam |
| Vasopressin-regulated water reabsorption | 2 | 5.00E-03 | Dctn5,Dync1li1 |
| Gastric acid secretion | 2 | 6.50E-03 | Prkca,Slc9a4 |
| ErbB signaling pathway | 2 | 6.50E-03 | Prkca,Abl2 |
| Neuroactive ligand-receptor interaction | 3 | 6.50E-03 | Gabra5,Npy2r,Grik4 |
| Pancreatic secretion | 2 | 7.60E-03 | Prkca,Pnliprp2 |
| Metabolic pathways | 5 | 9.30E-03 | Pet112l,Crls1,Prps1,Cox7b2,Pnliprp2 |
| Wnt signaling pathway | 2 | 1.23E-02 | Prickle2,Prkca |
| Endocytosis | 2 | 2.14E-02 | Ap2a2,Acap2 |
Supplementary Table 3
